# Positive and negative feedback regulation of the TGF-β1 explains two equilibrium states in skin aging

**DOI:** 10.1016/j.isci.2024.109708

**Published:** 2024-04-10

**Authors:** Masatoshi Haga, Keita Iida, Mariko Okada

**Affiliations:** 1 Institute for Protein Research, Osaka University, Suita, Osaka 565-0871, Japan; 2 Basic Research Development Division, ROHTO Pharmaceutical Co., Ltd, Osaka 544-8666, Japan; 3 Premium Research Institute for Human Metaverse Medicine (WPI-PRIMe), Osaka University, Suita, Osaka 565-0871, Japan

**Keywords:** Dermatology, Cell biology, Mathematical biosciences, Systems biology, Omics

## Abstract

During aging, skin homeostasis is essential for maintaining appearance, as well as biological defense of the human body. In this study, we identified thrombospondin-1 (THBS1) and fibromodulin (FMOD) as positive and negative regulators, respectively, of the TGF-β1-SMAD4 axis in human skin aging, based on *in vitro* and *in vivo* omics analyses and mathematical modeling. Using transcriptomic and epigenetic analyses of senescent dermal fibroblasts, TGF-β1 was identified as the key upstream regulator. Bifurcation analysis revealed a binary high-/low-TGF-β1 switch, with THBS1 as the main controller. Computational simulation of the TGF-β1 signaling pathway indicated that THBS1 expression was sensitively regulated, whereas FMOD was regulated robustly. Results of sensitivity analysis and validation showed that inhibition of SMAD4 complex formation was a promising method to control THBS1 production and senescence. Therefore, this study demonstrated the potential of combining data-driven target discovery with mathematical approaches to determine the mechanisms underlying skin aging.

## Highlights

• Multi-omics analysis identified TGF-β1-SMAD4 as key regulators in human skin aging
• THBS1 promoted and FMOD suppressed factors in skin aging
• THBS1 controlled both high- and low-TGF-β1 states of the binary switch
• SMAD4 is a potential target for inhibiting THBS1 expression and cell senescence

## Graphical abstract

**Figure:**
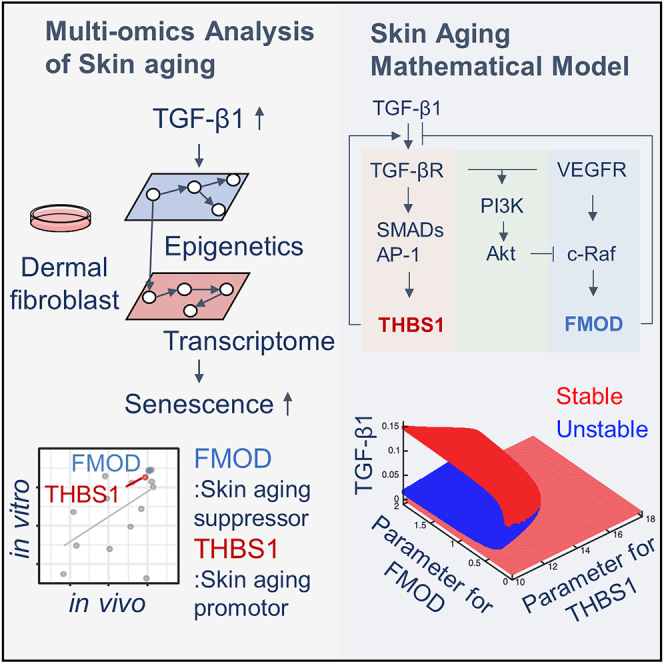

## Introduction

Aging is an inevitable and irreversible physiological phenomenon that disrupts the homeostasis of human body and rapidly increases risk of diseases such as skin cancer.^1^^,^^2^ Skin is the largest organ of the human body, which acts as a barrier to external environments and protects against unwanted external stimuli.^3^ Skin aging leads to decreased collagen production and elasticity, increased inflammation, and less mechanical resistance to skin damage, ultimately resulting in skin sagging and wrinkles.^4^ Maintaining skin homeostasis during aging is important for not only physical appearance but also biological defense; as the skin becomes thinner and more fragile, the development of skin diseases gets accelerated.

Skin aging is caused by both external (such as UV exposure, smoking, and environmental pollution)^5^ and internal factors (such as impaired skin repair and hormonal imbalance).^6^ Cellular senescence is a critical internal factor for skin aging.^7^^,^^8^ Skin tissue mainly consists of the epidermis and dermis. Keratinocytes, which make up the epidermis, have less influence on senescence because they have a higher turnover than fibroblasts in the dermis.^9^ In the dermis, skin aging results in the accumulation of senescent fibroblasts.^10^^,^^11^^,^^12^^,^^13^ Fibroblasts orchestrate the development of a functional skin barrier through crosstalk between the dermis and epidermis.^14^ Incorporation of senescent fibroblasts into a 3D equivalent model of human skin can reproduce typical changes associated with skin aging.^15^^,^^16^ Early or late passage fibroblasts could mimic young and aged skin, respectively, and the late passage fibroblasts thinned the dermis and induced epidermal differentiation.^15^ Another 3D skin model using oxidative stress-induced premature senescent fibroblasts caused progressive thinning of the epidermis with an increase in premature senescent cells in the dermis.^16^ These studies strongly suggest that regulation of fibroblast senescence is fundamental to maintaining the skin barrier and aging.

Senescent cells exhibit irreversible terminal growth arrest with flattened large-cell morphology, shortened telomeres, increased levels of the cyclin-dependent kinase inhibitors p16 or p53/p21, and secretion of the senescence-associated secretory phenotype (SASP) consisting of inflammatory cytokines, such as interleukin (IL)-6 and IL-8.^9^^,^^17^ Senescence-associated β-galactosidase (SA-β-Gal) activity is another well-known biomarker of cellular senescence. Various stressors (e.g., replication stress, DNA double-strand breaks, oncogene activation, and reactive oxygen species) induce cellular senescence.^18^ However, cellular senescence has not yet been fully elucidated at the systems level.

Data-driven multi-omics approaches have been used in various studies to elucidate the complex mechanisms of skin aging.^19^^,^^20^ RNA-seq, DNA methylation, histone methylation (histone H3 lysine 4 tri-methylation [H3K4me3], and histone H3 lysine 27 tri-methylation [H3K27me3]) from healthy skin fibroblasts of donors aged 35 to 75 years revealed an age-dependent decrease in the expression of genes involved in translation and ribosomal function.^19^ An assay for transposase-accessible chromatin with high-throughput sequencing (ATAC-seq) and RNA-seq analyses of healthy and Hutchinson-Gilford progeria syndrome (HGPS, a premature aging syndrome) skin fibroblasts revealed altered chromatin accessibility enriched in lamina-associated domains (LAD) and HGPS-specific gene expression.^20^ Although multi-omics approaches can capture the landscape of gene regulation, it remains difficult to identify key factors and regulatory structures among the thousands of genes involved in skin aging. Mathematical modeling combined with omics analysis provides a potential solution to enhance our understanding of the key mechanisms of skin aging controlled by genetic and environmental factors.^21^ Mathematical models have been used to represent human disease as a gene regulatory network and stratify patients, identify key regulators, and predict drug targets.^22^^,^^23^^,^^24^ As the use of animals in skin cosmetic research is prohibited in the industry,^25^ insights from simulations with mathematical models are important to study skin aging.

In this study, the mechanisms of human skin aging were identified through multi-omics analysis of *in vitro* and *in vivo* data and mathematical modeling. Replication stress was introduced into human dermal fibroblast HFF-1 cells (senescent cell model) and three stages (early: population doubling level (PDL) 24, intermediate: PDL 36, late; PDL 47) were obtained in the PDL, representing the total number of cell population doublings *in vitro*. Analyses of RNA-seq, chromatin immunoprecipitation sequencing (ChIP-seq) for histone H3 lysine 27 (H3K27Ac) modification, and ATAC-seq of different passages of HFF-1 cells and two independent *in vitro* and *in vivo* RNA-seqs of fibroblasts^26^^,^^27^ identified transforming growth factor β1 (TGF-β1) as a key regulator of internal and external aging. The levels of thrombospondin-1 (THBS1) and fibromodulin (FMOD) were highly correlated with PDL or age both *in vitro* and *in vivo*. THBS1 is known to activate the latent form of TGF-β1^28^ while FMOD binds to TGF-β1 and inhibits its binding to the TGF-β receptor.^29^^,^^30^^,^^31^ Inhibitor and knockdown (KD) assays implicated the signaling network for THBS1 and FMOD production. Finally, mathematical modeling and bifurcation analysis revealed a regulatory network mechanism by TGF-β1, THBS1, and FMOD in skin aging. This study provides important insights into the network mechanisms that drive and control skin aging and cellular senescence.

## Results

### Multi-omics analysis reveals TGF-β1 as a potential regulator of skin aging

**Figure 1:**
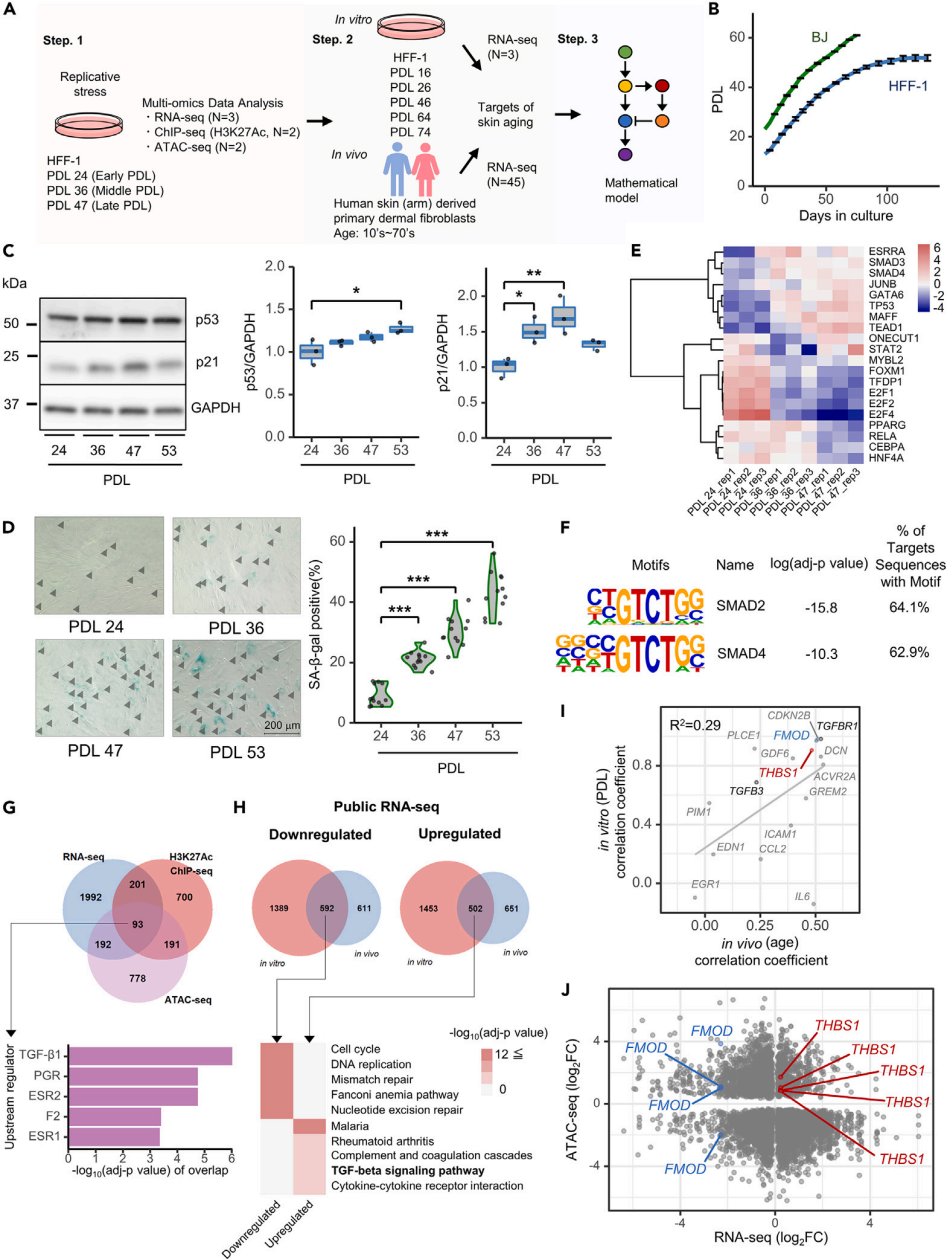
Integrated multi-omics analysis reveals TGF-β1 as a central modulator of skin aging and senescence (A) Workflow of skin aging and senescence analyses. In step one, replication stress was induced by passage culturing using human foreskin fibroblasts, HFF-1, to prepare each PDL (see STAR Methods), and generate data on RNA-seq, ChIP-seq with H3K27Ac antibody, and ATAC-seq. In the second step, two independent public RNA-seq datasets were analyzed, one *in vitro*^26^ and another *in vivo.*^27^ In the third step, we constructed a mathematical model based on the multi-omics analysis and *in vitro* experimental results. (B) Growth curve of HFF-1 (blue line) and BJ cells (green line); *N* = 3, mean ± SD. (C) Western blot (WB) of p53 and p21 in replication-stress-induced HFF-1 cells. Cells were cultured for 48 h, and lysates were analyzed. The horizontal line in the center of the box plot is the median, the lower and upper borders indicate the 25th and 75th percentiles, and the whiskers extend to the minimum and maximum values, respectively. (Left panel) Representative image. (Middle panel) Quantification of p53 expression; *N* = 3, ∗*p* < 0.05 (Dunnett’s test). (Right panel) Quantification of p21 expression; *N* = 3, ∗*p* < 0.05, ∗∗*p* < 0.01 (Dunnett’s test). (D) (Left panel) SA-β-gal staining of replication-stress-induced HFF-1 cells. Representative images. SA-β-gal-positive cells are indicated by black arrowheads; scale bar: 200 μm. See Figure S1A for processed images. (Right panel) Quantification of SA-β-gal: SA-β-gal-positive rate (%) = number of SA-β-gal positive cells/total number of cells × 100. Four images per well were randomly analyzed from three wells (total of 12 images/PDL), ∗∗∗*p* < 0.001 (Dunnett’s test). (E) Transcription factor (TF) enrichment analysis of RNA-seq data derived from replication-stress-induced HFF-1 cells of top 20 TFs (see details in STAR Methods section). Heatmap shows the normalized TF enrichment scores calculated using the DoRothEA analysis. (F) Enriched motifs in the gained ATAC-seq peaks with increase of PDL. log(adj-p value) and proportion of target sequences with motif was calculated using the “findMotifsGenome.pl” function of HOMER. (G) (Upper panel) Venn diagram showing RNA-seq differentially expressed genes (DEGs; blue sphere; |fold change (FC)| > 1.2, adj-p<0.05), genes annotated from ATAC differential peaks (purple sphere; |log_2_FC| > 0, adj-p<0.05), and genes annotated from H3K27Ac differential peaks (red sphere; |log_2_FC| > 0, adj-p<0.05) between PDL 24, PDL 36, and PDL 47. The number of genes in each condition is shown in the Venn diagram. (Lower panel) The top five upstream regulators found using Ingenuity Pathway Analysis (IPA) are shown with epigenetic-linked DEGs (93 genes). A right-tailed Fisher’s exact test was used to calculate –log_10_(adj-p) of overlap. (H) (Upper panel) Venn diagram showing the public *in vitro* DEGs (blue sphere) and *in vivo* DEGs (red sphere). (Lower panel) Heatmap showing top five pathways annotated in the pathway enrichment analysis (Kyoto Encyclopedia of Genes and Genomes [KEGG]). The adj-p was calculated using “compareCluster” of clusterProfiler. (I) Correlation coefficient between the public *in vitro* (PDL), public *in vivo* (age), and gene expression data. The genes associated with KEGG term “TGF-beta signaling pathway” in Figure 1H are plotted. Spearman correlations were calculated using the “cor” function of R software; *THBS1* (red) and *FMOD* (blue). R^2^ was calculated using the “ggpmisc::stat_poly_eq” function. (J) Correlation between the expression FC (control vs. 4 ng/mL TGF-β1; adj-p<0.05) of RNA-seq and peak FC (control vs. 4 ng/mL TGF-β1; adj-p<0.05) of ATAC-seq; *THBS1* (red) and *FMOD* (blue). FC and adj-p were calculated using DESeq2.

To identify global transcriptional signatures and their regulatory mechanisms in dermal fibroblast senescence, we performed RNA-seq, ChIP-seq (for H3K27Ac modification), and ATAC-seq of replicative stressed HFF-1 cells (Figure 1A). First, HFF-1 and BJ, a different cell line of human dermal fibroblasts, were maintained from early (HFF-1: PDL 13, BJ: PDL 23) to late (HFF-1: PDL 53, BJ: PDL 61) PDL to induce replication stress (Figure 1B). We examined the activities of p53, p21, and SA-β-gal for different PDLs of fibroblast cells (HFF-1: Figures 1C, 1D, and S1A; BJ; Figures S1B and S1C). Slower cell proliferation was observed with increased PDL in HFF-1 cells (Figure S1D). Replication stress-induced cellular senescence was promoted by increased PDL in both cell lines. To explain the decline of p21 in PDL 53 in HFF-1 cells (Figure 1C), we focused on USP11—a deubiquitylase that stabilizes p21 protein and prevents its degradation^32^—and observed a significant decrease in USP11 expression with increased PDL in HFF-1 cells (Figure S1E). To identify transcription factors (TFs) that regulate senescence in HFF-1 cells, the TF enrichment score was calculated from the gene expression of each PDL using DoRothEA analysis,^33^ and the top 20 TFs were determined based on the enrichment score (Figure 1E). The downstream TFs of the TGF-β pathway, SMAD3 (PDL 24 vs. PDL 36: *p* < 0.01, PDL 24 vs. PDL 47: *p* < 0.05) and SMAD4 (PDL 24 vs. PDL 36: *p* < 0.05, PDL 24 vs. PDL 47: *p* = 0.057), were enriched with increasing PDL. We also identified known senescence-associated TFs, including JUNB,^34^ GATA6,^35^ TP53,^36^ TEAD1,^34^ and E2F4.^34^ To confirm whether SMAD motifs indeed played an important role with cellular senescence, we performed a TF motif enrichment analysis using HOMER,^37^ by assessing the gained ATAC peaks—the open chromatin regions specifically enriched for late PDL (Figure 1F). As a result, we found that SMAD2 (–log[adj-p]: 15.8) and SMAD4 (–log[adj-p]: 10.3) were associated with the gained ATAC peaks. Next, we analyzed whether TGF-β is the upstream regulator of cellular senescence in dermal fibroblasts from 93 overlapping differentially expressed genes (DEGs) identified in the RNA-seq, differential ATAC peaks, and differential H3K27Ac peaks (Figure 1G) between PDL 24, PDL 36, and PDL 47. TGF-β1 was identified as the top regulator among the overlapping DEGs. From the 215 common genes shared between upregulated DEGs and genes annotated from differentially gained peaks of H3K27Ac ChIP-seq, indicating active enhancer regions, TGF-β1, TGF-β3, and SMAD3 were the top three upstream regulators (Figure S2A). These results indicate that TGF-β1 signaling pathway plays a critical role in the senescence of dermal fibroblasts.

To further confirm the involvement of TGF-β1 in skin aging and senescence, two additional independent public RNA-seq datasets were analyzed: *in vivo* data for primary human dermal fibroblasts^27^ representing a wide age range (11–71 years of age, see donor list in Table S1), to analyze external factors, and *in vitro* data for HFF-1 cells over a long-term passage,^26^ to analyze internal factors (PDL 16–74, five PDL points). For *in vivo* data, we obtained three clusters of donors based on whole gene expression data and identified DEGs among the clusters (Figures S2B and S2C). For *in vitro* data, DEGs between each PDL were identified. The number of overlapping DEGs in both datasets corresponded to 592 downregulated and 502 upregulated genes (Figure 1H). Functional analysis of overlapping gene sets using the Kyoto Encyclopedia of Genes and Genomes (KEGG) database^38^ revealed that the cell cycle and TGF-β signaling pathways were enriched in both datasets. In addition, TGF-β1 was identified as the top regulator for the upregulated gene set by upstream analysis of the overlapping gene sets (Figure S2D). In fact, the RNA-seq expression of *TGFB1* was upregulated with the increase in PDL (Figure S2E). Comparisons between gene expression and *in vivo* age or *in vitro* PDL revealed that *THBS1* and *FMOD* expression were strongly correlated with skin aging (*in vivo*) and senescence (*in vitro*) (R^2^ = 0.29) (Figure 1I). In both data, THBS1 and FMOD show the same time course dynamics with *in vivo* age and *in vitro* PDL (Figures S3A and S3B). Further, the expression of the TGF-β receptor, *TGFBR1*, and *TGFB3* was also correlated with gene expression levels both *in vivo* and *in vitro*, indicating that TGF-β signaling is more likely to be activated in skin aging. As THBS1 has been associated with aging in other organs^39^ and FMOD is responsible for cross-linking of collagen^40^—which decreases with age in skin tissue^4^—we focused on TGF-β1, THBS1, and FMOD for further analysis. To investigate the effects of modulation of TGF-β1, THBS1, and FMOD, we obtained RNA-seq and ATAC-seq of TGF-β1 treated HFF-1 cells (Figure 1J). Altered expression levels and peak changes in THBS1 and FMOD were observed in the datasets. These results indicated that TGF-β1 is an upstream factor in aging and cellular senescence, affecting THBS1 and FMOD expression through epigenetic changes.

**Figure 2:**
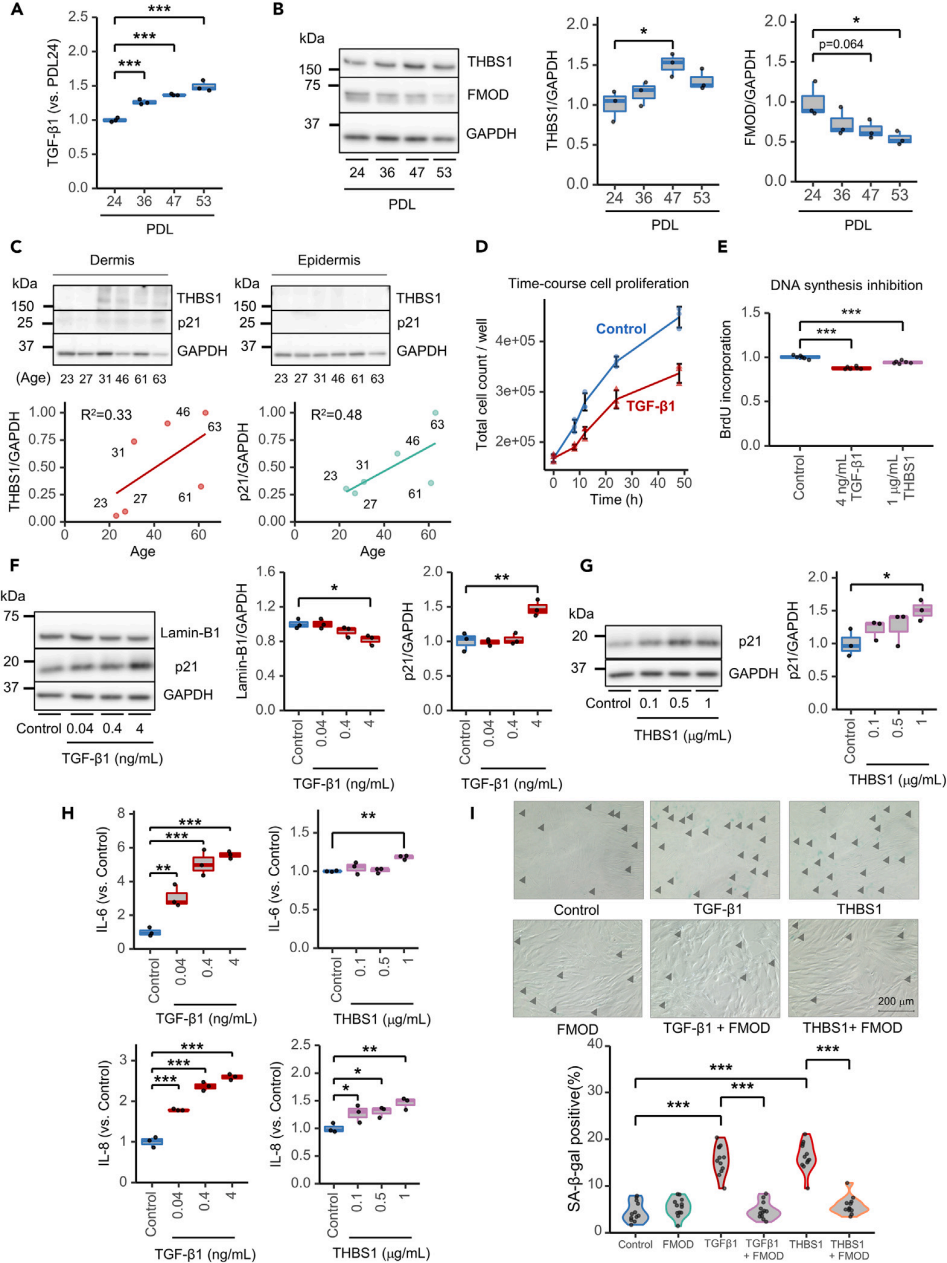
TGF-β1 and THBS1 act as senescence-inducing factors, while FMOD acts as a senescence inhibitor (A) TGF-β1 ELISA in replication-stress-induced HFF-1 cell supernatants. Cells were cultured for 48 h, and supernatants were analyzed. The horizontal line in the center of the box plot is the median, the lower and upper borders indicate the 25th and 75th percentiles, and the whiskers extend to the minimum and maximum values, respectively; *N* = 3, ∗∗∗*p* < 0.001 (Dunnett’s test). (B) WB analysis of THBS1 and FMOD in replication-stress-induced HFF-1 cells. Cells were cultured for 48 h, and lysates were analyzed. The horizontal line in the center of the box plot is the median, the lower and upper borders indicate the 25th and 75th percentiles, and the whiskers extend to the minimum and maximum values, respectively. (Left panel) Representative image. (Middle panel) Quantification of THBS1 expression; *N* = 3, ∗*p* < 0.05 (Dunnett’s test). (Right panel) Quantification of FMOD expression; *N* = 3, ∗*p* < 0.05 (Dunnett’s test). (C) WB analysis of THBS1 and p21 in human dermal and epidermal tissues (*N* = 6). Data were normalized to the maximum (1) value. R^2^ was calculated using the “stat_poly_eq” function of R. (Upper panel) Age of each donor is displayed at the bottom of the image (see donor list in Table S2). (Lower left panel) Quantification of THBS1 expression in dermis. (Lower right panel) Quantification of p21 expression in dermis. (D) Time course cell proliferation analysis of control (blue-circled dots) or 4 ng/mL TGF-β1-treated (red triangular dots) cells stained with trypan blue; *N* = 3, mean ± SD. (E) BrdU incorporation assay: control (blue), 4 ng/mL TGF-β1 (red), or 1 μg/mL THBS1 (purple). The horizontal line in the center of the box plot is the median, the lower and upper borders indicate the 25th and 75th percentiles, and the whiskers extend to the minimum and maximum values, respectively; *N* = 6, ∗∗∗*p* < 0.001 (Dunnett’s test). (F) WB analysis of lamin-B1 and p21 in TGF-β1-stimulated HFF-1 cells. Cell lysates were collected 48 h after control (blue) or TGF-β1 (red) treatment. The horizontal line in the center of the box plot is the median, the lower and upper borders indicate the 25th and 75th percentiles, and the whiskers extend to the minimum and maximum values, respectively. (Left panel) Representative image. (Middle panel) Quantification of lamin-B1; *N* = 3, ∗*p* < 0.05 (vs. control, Dunnett’s test). (Right panel) Quantification of p21; *N* = 3, ∗∗∗*p* < 0.001 (Dunnett’s test). (G) Western blot of p21 in THBS1-stimulated HFF-1 cells. Cell lysates were collected 48 h after control (blue) or THBS1 (purple) treatment. The horizontal line in the center of the box plot is the median, the lower and upper borders indicate the 25th and 75th percentiles, and the whiskers extend to the minimum and maximum values, respectively. (Left panel) Representative image. (Right panel) Quantification of p21; *N* = 3, ∗*p* < 0.05 (Dunnett’s test). (H) IL-6 and IL-8 ELISA in TGF-β1- or THBS1-stimulated HFF-1 cells. Cell supernatants were collected 48 h after TGF-β1 (red) or THBS1 (purple) treatment. The horizontal line in the center of the box plot is the median, the lower and upper borders indicate the 25th and 75th percentiles, and the whiskers extend to the minimum and maximum values, respectively. (Upper left panel) Quantification of IL-6 ELISA with TGF-β1 treatment; *N* = 3, ∗∗*p* < 0.01, ∗∗∗*p* < 0.001 (Dunnett’s test). (Upper right panel) Quantification of IL-6 ELISA with THBS1 treatment; *N* = 3, ∗∗*p* < 0.01 (Dunnett’s test). (Bottom left panel) Quantification of IL-8 ELISA with TGF-β1 treatment; *N* = 3, ∗∗*p* < 0.01, ∗∗∗*p* < 0.001 (Dunnett’s test). (Bottom right panel) Quantification of IL-8 ELISA with THBS1 treatment; *N* = 3, ∗∗*p* < 0.01 (Dunnett’s test). (I) Effect of TGF-β1 or THBS1 treatment and inhibition by FMOD on SA-β-gal activity. HFF-1 cells were treated with control, 4 ng/mL TGF-β1, 0.5 μg/mL THBS1, or 8 ng/mL FMOD. Combinations of 4 ng/mL TGF-β1 and 8 ng/mL FMOD as well as 0.5 μg/mL THBS1 and 8 ng/mL FMOD were performed alongside stand-alone treatments. (Left panel) Representative images. SA-β-gal-positive cells are shown with black arrowhead; scale bars: 200 μm. See Figure S4E for processed images. (Right panel) Quantification of SA-β-gal: SA-β-gal-positive rate (%) = number of SA-β-gal-positive cells/total number of cells × 100. Four images per well were randomly analyzed from three wells (total 12 images/condition), ∗∗∗*p* < 0.001 (Tukey’s multiple comparisons).

The percentage of TGF-β1-positive fibroblasts in the human dermis increases with age.^41^ Higher PDL corresponded with higher TGF-β1 expression in HFF-1 cells (Figures 2A and S2E). Western blot (Figure 2B), RNA-seq (Figure S3C), qPCR (Figure S3D), and enzyme-linked immunosorbent assay (ELISA) analyses (Figure S3E) revealed an increased and decreased expression of THBS1 and FMOD, respectively, in higher cell passages of dermal fibroblasts. The expression of THBS1 was confirmed to increase with age in human dermal tissue isolated from female donors (23–63 years of age; Figure 2C, see Table S2 for donor list). From the same donors, p21 expression was also confirmed to increase with age, suggesting that our model of cellular senescence induced by long-term passaged culturing accurately captured changes in human skin factors associated with aging. It is worth noting that THBS1 was not detected in the epidermis, suggesting that THBS1 functions in the dermis during skin aging. Interestingly, using publicly available transcriptome data^27^ (see Table S3 for donor list), *THBS1* expression was found to be increased in patients with HGPS (2–8 years of age) compared to that in healthy donors (1–9 years of age) of the same age (Figure S3F). Note that, while our RNA-seq data using HFF-1 cells showed a decrease in FMOD with senescence (Figure S3C), public *in vitro* RNA-seq^26^ using the same HFF-1 cell line showed an opposite trend, i.e., increase of FMOD with senescence (Figure S3B). The results from our data-driven analysis identified the TGF-β1–SMAD signaling pathway along with THBS1 and FMOD expression as critical factors regulating skin aging and dermal fibroblast senescence.

### TGF-β1 and THBS1 induce senescence of human dermal fibroblasts

Next, we investigated the biological functions of TGF-β1 and THBS1 in terms of the senescence of human dermal fibroblasts. We first assessed the proliferation of HFF-1 cells (Figure 2D) and found that TGF-β1 treatment suppressed HFF-1 growth over time (8 h, 12 h, 24 h, and 48 h) compared to that of untreated cells. As with TGF-β1, THBS1 treatment resulted in a concentration-dependent decrease in cell viability (Figure S4A). TGF-β1 and THBS1 treatments were confirmed to inhibit DNA synthesis by BrdU incorporation experiments (Figure 2E). The levels of the senescence markers lamin-B1 decreased and p21 increased in a TGF-β1 dose-dependent manner (Figure 2F). Like the TGF-β1 treatment, THBS1 enhanced p21 expression (Figure 2G), confirming that TGF-β1- or THBS1-dependent suppression of cell proliferation is caused by cellular senescence. TGF-β1 or THBS1 treatment of HFF-1 cells induced the production of the pro-inflammatory SASPs IL-6 and IL-8, which are known to be secreted with cellular senescence^42^^,^^43^ (Figure 2H). TGF-β1 treatment also decreased lamin-B1 expression (Figure S4B) and increased IL-6, IL-8 (Figure S4C), and SA-β-gal production (Figure S4D) in BJ cells. In addition, treatment with TGF-β1 or THBS1 significantly increased the number of SA-β-gal-positive cells (Figures 2I and S4E). Interestingly, FMOD alone did not affect SA-β-gal activity; however, in combination treatments, FMOD suppressed the effects of TGF-β1 or THBS1 on SA-β-gal activation. These results strongly suggest that TGF-β1 and THBS1 promote the senescence of skin fibroblasts and this effect is suppressed by FMOD.

### THBS1 and FMOD expression is controlled by the TGF-β signaling pathway

**Figure 3:**
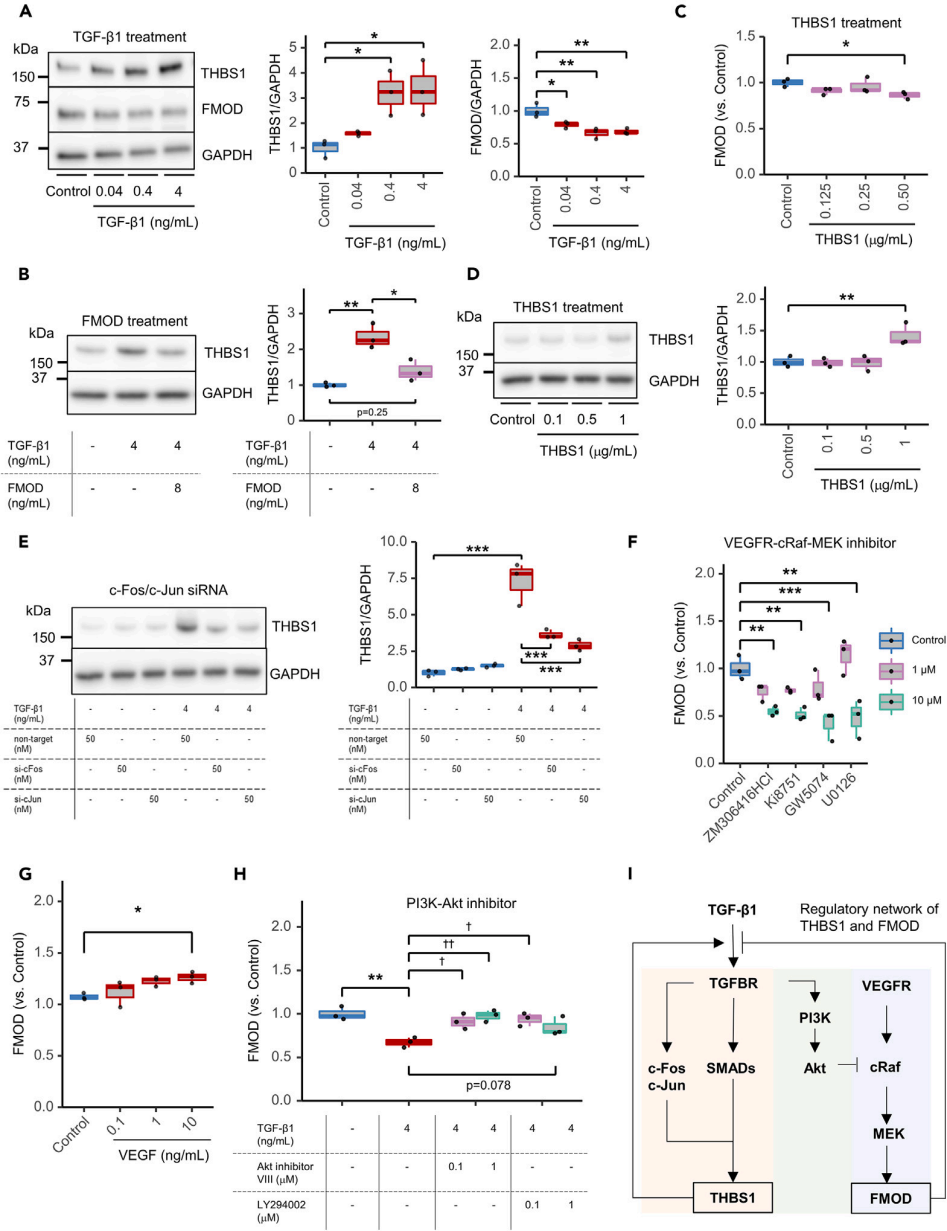
Regulatory network of THBS1 and FMOD in skin aging (A) WB of THBS1 and FMOD in TGF-β1-stimulated HFF-1 cells. Cell lysates were collected 48 h after control (blue) or TGF-β1 (red) treatment. The horizontal line in the center of the box plot is the median, the lower and upper borders indicate the 25th and 75th percentiles, and the whiskers extend to the minimum and maximum values, respectively. (Left panel) Representative image. (Middle panel) Quantification of THBS1; *N* = 3, ∗*p* < 0.05 (vs. control, Dunnett’s test). (Right panel) Quantification of FMOD; *N* = 3, ∗*p* < 0.05, ∗∗*p* < 0.01 (Dunnett’s test). (B) WB analysis of THBS1 in TGF-β1- and FMOD-stimulated HFF-1 cells. Cell lysates were collected 48 h after treatment with control (blue), 4 ng/mL TGF-β1 (red), or a combination of 4 ng/mL TGF-β1 and 8 ng/mL FMOD (purple). The horizontal line in the center of the box plot is the median, the lower and upper borders indicate the 25th and 75th percentiles, and the whiskers extend to the minimum and maximum values, respectively.(Left panel) Representative image. (Right panel) Quantification of THBS1; *N* = 3, ∗*p* < 0.05, ∗∗*p* < 0.01 (Tukey’s multiple comparisons). (C) FMOD ELISA of THBS1-stimulated HFF-1 cells. Cells were treated with THBS1 (purple) for 48 h and their supernatants were analyzed. The horizontal line in the center of the box plot is the median, the lower and upper borders indicate the 25th and 75th percentiles, and the whiskers extend to the minimum and maximum values, respectively; *N* = 3, ∗*p* < 0.01 (Dunnett’s test). (D) WB analysis of THBS1 in THBS1-stimulated BJ cells. Cell lysates were collected 48 h after control (blue) or THBS1 (purple) treatment. The horizontal line in the center of the box plot is the median, the lower and upper borders indicate the 25th and 75th percentiles, and the whiskers extend to the minimum and maximum values, respectively. (Left panel) Representative image. (Right panel) Quantification of THBS1; *N* = 3, ∗∗*p* < 0.01 (Dunnett’s test). (E) WB analysis of THBS1 with c-Fos/c-Jun knockdown (KD) using HFF-1 cells. Cells were pretreated with each siRNA (50 nM) and collected 48 h after control (blue) or 4 ng/mL TGF-β1 (red) treatment. The horizontal line in the center of the box plot is the median, the lower and upper borders indicate the 25th and 75th percentiles, and the whiskers extend to the minimum and maximum values, respectively. (Left panel) Representative image. (Right panel) Quantification of THBS1; *N* = 3, ∗∗*p* < 0.01 (Tukey’s multiple comparisons). (F) FMOD ELISA of kinase inhibitor-treated HFF-1 cells. Cells were treated with ZM306416HCl, Ki8751, GW5074, or U0126 for 48 h and their supernatants were analyzed. The horizontal line in the center of the box plot is the median, the lower and upper borders indicate the 25th and 75th percentiles, and the whiskers extend to the minimum and maximum values, respectively; *N* = 3, ∗∗*p* < 0.01, ∗∗∗*p* < 0.001 (Dunnett’s test). (G) FMOD ELISA of VEGF165 treated HFF-1 cells. Cells were treated with VEGF165 for 48 h and the supernatants were analyzed. The horizontal line in the center of the box plot is the median, the lower and upper borders indicate the 25th and 75th percentiles, and the whiskers extend to the minimum and maximum values, respectively; *N* = 3, ∗*p* < 0.05 (Dunnett’s test). (H) FMOD ELISA in kinase inhibitor- and TGF-β1-treated HFF-1 cells. Cells were treated with Akt inhibitor VIII or LY294002 combined with 4 ng/mL TGF-β1 for 48 h and their supernatants were analyzed. The horizontal line in the center of the box plot is the median, the lower and upper borders indicate the 25th and 75th percentiles, and the whiskers extend to the minimum and maximum values, respectively; *N* = 3, ∗∗*p* < 0.01 (Student’s t test), ^†^*p* < 0.05, ^††^*p* < 0.01 (Dunnett’s test). (I) Regulatory network of THBS1 and FMOD. In human dermal fibroblasts, TGF-β1 induced THBS1 production occurs via TGF-βR–SMAD activation (red shade). FMOD was regulated via activation of the VEGFR-cRaf-MEK pathway (blue shade). Crosstalk between these pathways occurred with the TGF-β pathway inhibiting the VEGF pathway via the PI3K-Akt pathway (green shade).

THBS1 is known to activate latent TGF-β1,^28^ while FMOD reportedly binds to TGF-β1 to inhibit its binding to the TGF-β receptor.^29^^,^^30^^,^^31^ THBS1 is also known to be induced by TGF-β1 stimulation in human dermal fibroblasts.^44^ Consistently, we found that THBS1 expression was induced by TGF-β1 in HFF-1 cells (Figure 3A). However, the same TGF-β1 treatment decreased FMOD expression. These results were also confirmed in BJ cells (Figures S4B and S4F). Next, the effect of FMOD on THBS1 expression was examined in HFF-1 cells (Figure 3B). While TGF-β1 alone increased THBS1 expression, the addition of FMOD completely suppressed THBS1 levels. In contrast, THBS1 treatment downregulated FMOD expression in a dose-dependent manner (Figure 3C). These findings suggest a mutual inhibitory role for THBS1 and FMOD. Interestingly, stimulation of BJ cells with THBS1 enhanced its own expression (Figure 3D), suggesting the positive-feedback regulation of the TGF-β pathway by THBS1 in dermal fibroblasts. We additionally investigated the effects of other TGF-β family members, particularly TGF-β2 and TGF-β3, on the expression of THBS1 and FMOD, and found that THBS1 expression was promoted and FMOD expression was suppressed in all members of the TGF-β family (Figures S4G and S4H). Therefore, TGF-β2 and TGF-β3 may also be involved in skin aging, but they were found to regulate THBS1 and FMOD via a common mechanism as TGF-β1.

We investigated the regulation of THBS1 and FMOD by TGF-β1 and related signaling pathways using small molecule inhibitors and small interfering (si) RNA KD experiments. TGF-β1-dependent induction of THBS1 and phosphorylation of SMAD2 and SMAD3 were inhibited by LY364947 (TGF-βRI/TGF-βRII inhibitor) and SB431542 (TGF-βRI inhibitor; Figure S5A). Because both SMAD3 and SMAD4 cooperate with c-Fos/c-Jun of the activator protein-1 (AP-1) family to mediate TGF-β-induced transcription,^45^^,^^46^^,^^47^ we tested the involvement of c-Fos/c-Jun in the regulation of THBS1 using siRNA. c-Fos/c-Jun KD significantly reduced the TGF-β1-induced expression of THBS1 compared to that with non-targeted KD (Figure 3E). The siRNA of c-Fos/c-Jun showed downregulation of c-Fos/c-Jun expression without affecting the activation of SMAD2, SMAD3, and SMAD4 (Figure S5B). The AP-1 DNA binding inhibitor, T-5224, was also tested for its effect on THBS1 regulation in HFF-1 cells (Figure S6A). While T-5224 alone had no effect on THBS1 expression compared to TGF-β1 alone, the combination of TGF-β1 and T-5224 reduced THBS1 expression. We also confirmed that T-5224 of c-Fos/c-Jun did not affect the activation of SMAD2, SMAD3, and SMAD4 (Figure S6B). These findings suggest that regulation of THBS1 in dermal fibroblasts requires the formation of AND-gated networks through the activation of SMAD and binding of c-Fos/c-Jun to DNA, both of which are activated by TGF-β stimulation.

To investigate the regulatory mechanism of FMOD in dermal fibroblasts, we conducted a compound screening using various kinase inhibitors (Figure S7A). The candidates in the screening included Ki8751 (VEGFRII inhibitor), GW5074 (c-Raf1 inhibitor), and U0126 (MEK1/2 inhibitor). Alongside these compounds, ZM306416 (VEGFRI inhibitor) was also selected to validate the effect on FMOD expression, which was found to be significantly suppressed (Figure 3F). Further supporting this, the expression of FMOD was found to be significantly increased with higher concentrations of VEGF (Figure 3G). Additionally, other growth factors, such as epidermal growth factor (EGF), basic-fibroblast growth factor (b-FGF), and platelet-derived growth factor-BB (PDGF-BB), which also activate c-Raf and MEK, were found to reduce FMOD expression (Figure S7B). These results collectively suggest that FMOD is regulated by the VEGF signaling pathway in dermal fibroblasts.

To determine the mechanism of FMOD suppression by TGF-β1, we tested whether the combination of kinase inhibitor and TGF-β1 would restore FMOD expression (Figure 3H). Akt inhibitor VIII (Akt inhibitor) or LY294002 (PI3K inhibitor) significantly restored FMOD expression in the presence of TGF-β1. These results indicate that THBS1 and FMOD expression is regulated by the TGF-β and VEGF-Raf-ERK signaling pathway, respectively (Figure 3I), and that the TGF-β1-dependent suppression of FMOD is mediated by the PI3K-Akt pathway.

### Bifurcation analysis of dermal senescence

**Figure 4:**
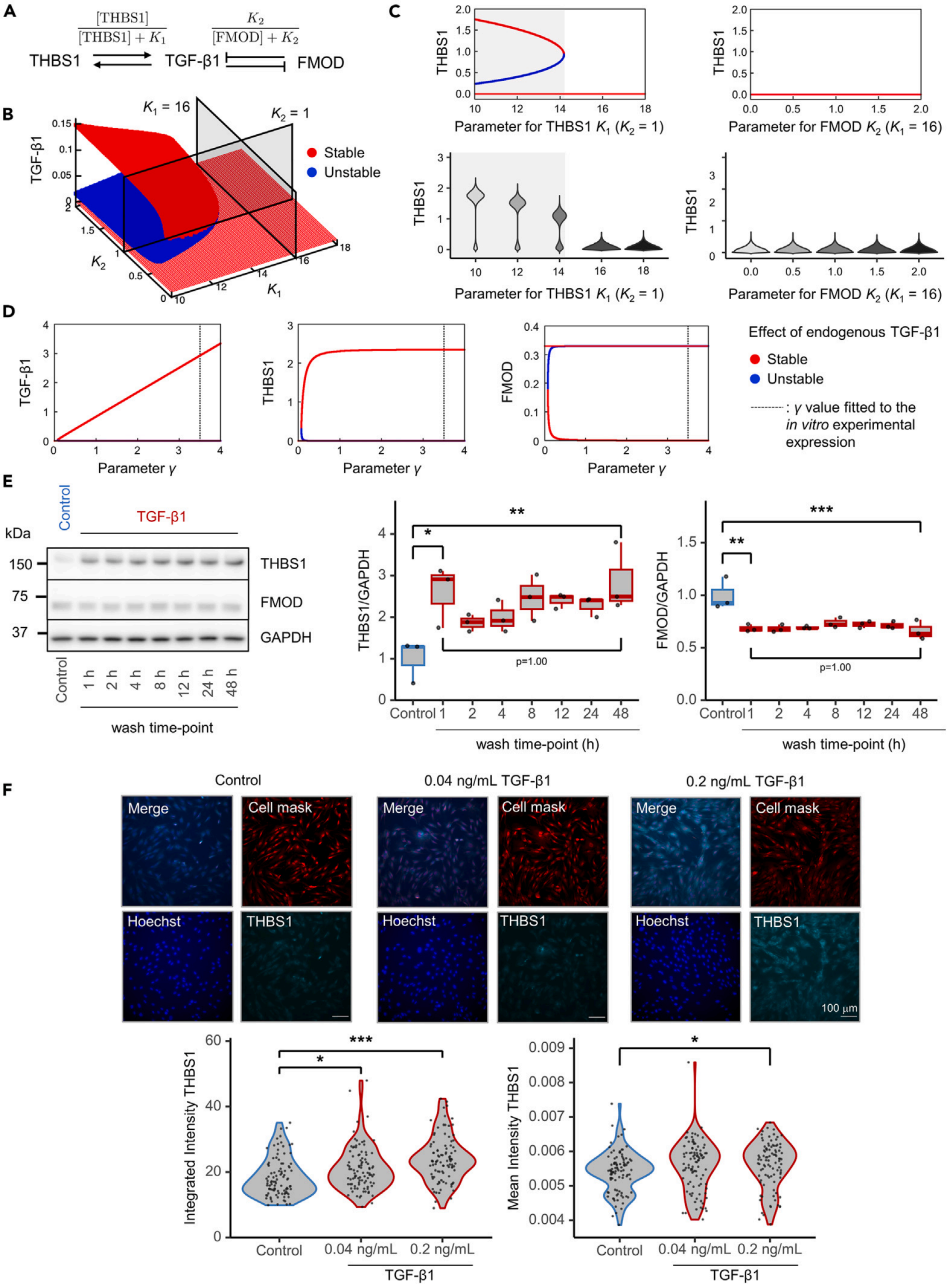
Bifurcation analysis and validation of skin aging (A) Core network consisting of TGF-β1, THBS1, and FMOD. Arrows and barred lines represent positive and negative regulations, respectively. (B) Bifurcation diagram of the steady state expression of TGF-β1 with respect to $K_{1}$ and $K_{2}$. Note that [TGFβ1] ≡0 is a stable solution for $K_{1}>0$ and $K_{2}>0$. (C) Bifurcation diagrams (upper panel) and probability distributions (bottom panel) for the expression level of THBS1 with respect to $K_{1}$ (left panel) and $K_{2}$ (right panel), which correspond to the cut surfaces shown in Figure 4B. Stable (red) and unstable (blue) fixed points are computed using a deterministic model, while distributions are computed using a stochastic model with 10,000 independent simulations. Gray shade indicates the change in $K_{1}$ from 14 (near the bifurcation point) to 10, which corresponds to an increase in TGF-β1. (D) Bifurcation diagrams of the steady state expressions of endogenous TGF-β1, THBS1, and FMOD with respect to γ, computed from the extended model. Red and blue points represent stable and unstable points, respectively, and vertical broken line at γ ≈ 3.5 represent the fitted value to the *in vitro* experimental expression upon replication-stress with different PDL. (E) WB analysis of time course TGF-β1 washout experiment using HFF-1 cells. Cells were treated with 4 ng/mL TGF-β1, washed with PBS at each time point (1 h, 2 h, 4 h, 8 h, 12 h, 24 h, and 48 h), and lysates were collected at 48 h. The horizontal line in the center of the box plot is the median, the lower and upper borders indicate the 25th and 75th percentiles, and the whiskers extend to the minimum and maximum values, respectively. (Left panel) Representative image. (Middle panel) Quantification of THBS1; *N* = 3, ∗*p* < 0.05, ∗∗*p* < 0.01 (Tukey’s multiple comparisons). (Right panel) Quantification of FMOD; *N* = 3, ∗∗*p* < 0.01, ∗∗∗*p* < 0.001 (Tukey’s multiple comparisons). (F) Distribution of THBS1 expression by immunofluorescence imaging using HFF-1 cells. Cells were treated with TGF-β1 for 48 h and fixed for immunofluorescence imaging. (Upper panel) Representative images; scale bars: 100 μm. (Bottom left panel) Quantification of integrated intensity of THBS1 of each image; 16 images per well from six wells were analyzed (total 96 images/condition); ∗*p* < 0.05, ∗∗∗*p* < 0.001 (Dunnett’s test). (Bottom right panel) Quantification of mean intensity of THBS1 of each image; 16 images per well from six wells were analyzed (total 96 images/condition); ∗*p* < 0.05 (Dunnett’s test).

A nonlinear ordinary differential equation (ODE) model of the core network consisting of TGF-β1, THBS1, and FMOD was constructed to qualitatively elucidate the behavior of the system (Figure 4A, see details in the STAR Methods section). Fitting against the datasets of THBS1 and FMOD levels at different TGF-β1 concentrations yielded parameters that reproduced the experimental results (Figure S8A), while the parameters for regulation of TGF-β1 by THBS1 ($K_{1}$) and FMOD ($K_{2}$) are yet to be identified. We examined which parameter is more influential for controlling the steady state, and found that both parameters can induce a bistable switch for TGF-β1 (Figure 4B). Since the expression levels of THBS1 and FMOD were upregulated and downregulated with the increase of TGF-β1 expressions, respectively, changing these parameters simulates the transition from low-to high-TGF-β1 states (Figure 3A). For the purpose of this study, we defined “high-TGF-β1” as the state with high-THBS1 and low-FMOD expressions, and “low-TGF-β1” as the state with low-THBS1 and high-FMOD expressions, respectively. Importantly, the bistable region was quite asymmetric with respect to $K_{1}$ and $K_{2}$ (Figure 4B): changes in $K_{1}$ always induced a transition from the current state, whereas changes in $K_{2}$ induced the transition only for a small parameter range of $K_{1}$ (Figure S8B). Moreover, we extended the core model to a stochastic model to simulate the probability distributions of THBS1 (see details in the STAR Methods section) (Figure 4C). As a result, we found that the bimodal distribution of THBS1 expression becomes relatively unimodal when the value of $K_{1}$ is changed from 14 (near the bifurcation point) to 10, which corresponds to increasing TGF-β1 levels. In contrast, changing the value of $K_{2}$ ($K_{1}=16$) does not produce bifurcation. Taken together, we concluded that $K_{1}$ has more influence on state transitioning.

To better understand the roles of endogenous TGF-β1, THBS1, and FMOD, we extended the aforementioned model as an endogenous core model to incorporate the effects of PDL and endogenous TGF-β1 production for simulating the system behavior in an endogenous condition (Figure 4D). By fitting the steady state values of THBS1, FMOD, and TGF-β1 against the corresponding *in vitro* experimental values for different PDLs (Figures 2A and 2B), we estimated the endogenous TGF-β1 normalized production rate as γ≈3.5 (Figure S8C). We found that the endogenous core model also shows binary high-/low-TGF-β1 states. Moreover, it was observed that THBS1 is sensitive against γ<1 (bifurcation point: γ≈0.07), where endogenous TGF-β1 production rate is low. Consequently, the nonlinear model was shown to provide a core network for assessing the molecular contributions to both skin aging and dermal fibroblast senescence.

### Irreversibility and bimodality of THBS1 in dermal senescence

Here, we demonstrated a binary high-/low-TGF-β1 switch through bifurcation analysis. To validate the conclusions drawn from the mathematical approaches, we conducted additional experiments. Firstly, we performed time course washout experiments of TGF-β1 treatment to confirm the irreversibility of the regulatory system, which was suggested by the existence of stable and unstable states (Figure 4E). TGF-β1 was washed out at various time points (i.e., 1 h, 2 h, 4 h, 8 h, 12 h, 24 h, and 48 h) and replaced with only the medium, which served as the control (Figure S9A). Interestingly, we found that both transient and sustained TGF-β1 treatments regulated THBS1 and FMOD expression in a similar manner. Furthermore, both treatments induced sustained activation of phosphorylated Akt (Ser473) (Figure S9B), implying that sustained stimulation is not essential for regulation of the system by TGF-β1 and that transient stimulation can reproduce the regulation. The regulation of THBS1 and Akt phosphorylation by transient and sustained stimulation was further confirmed with even shorter time point washes (i.e., 15 min, 30 min, 1 h, 4 h, 12 h, 24 h, and 48 h) (Figure S9C). These results indicated an irreversible nature of THBS1 upregulation and FMOD downregulation by TGF-β1 treatment, suggesting the presence of a binary switch.

Secondly, the effects of endogenous TGF-β signaling on THBS1, FMOD, and phosphorylated Akt (Ser473) expressions were confirmed through LY364947 treatment—a TGF-βRI/TGF-βRII inhibitor—performed during progressive passage-induced senescence at each PDL (Figure S10A). Inhibition of endogenous TGF-β signaling downregulated THBS1 but did not affect FMOD expression or the activation of phosphorylated Akt (Ser473). To further validate our *in vitro* findings with model simulations, we simulated the effect of TGF-βR inhibitor using the endogenous core model, by incorporating changes in the signaling pathways as the model parameter. Our stochastic simulations also showed that lowering the parameter γ for endogenous TGF-β1 production results in a decrease in THBS1 expression, while FMOD expression remains largely unchanged (see details in the STAR Methods section) (Figure S10B). These results indicate that when endogenous TGF-β signaling is inhibited, THBS1 is more likely to undergo a change of state, whereas FMOD remains barely sensitive.

Thirdly, to further validate the presence of a binary high-/low-TGF-β1 switch, we examined the bistability of THBS1 expression using immunofluorescence imaging (Figure 4F). Quantification of integral intensity of each image revealed that THBS1 expression was significantly upregulated by TGF-β1 in a concentration-dependent manner (Figure 4F), which was consistent with the results obtained through western blot (Figure 3A). Additionally, a comparison of the mean intensities of each individual immunostained image showed that THBS1 expression was significantly upregulated by TGF-β1 (control vs. 0.2 ng/mL TGF-β1: *p* < 0.05), while bimodal distribution of THBS1 expression was also observed in the control group (Silverman test;^48^ unimodal: *p* = 0.19, bimodal: *p* = 0.028). This result also demonstrated that the population of low-THBS1 cells (low TGF-β1) was reduced, whereas the population of high-THBS1 cells (high TGF-β1) increased by 0.2 ng/mL TGF-β1 (Silverman test; unimodal: *p* = 0.11, bimodal: *p* = 0.92). Overall, a distinct population of low-THBS1 cells remained present, although reduced by the effect of TGF-β1. A bimodal to unimodal shift in THBS1 expression by TGF-β1 was suggested based on the simulations of bifurcation analysis (Figure 4C). These findings support the existence of a binary high-/low-TGF-β1 switch in the network consisting of TGF-β1, THBS1, and FMOD.

### Mechanistic mathematical model of skin aging

**Figure 5:**
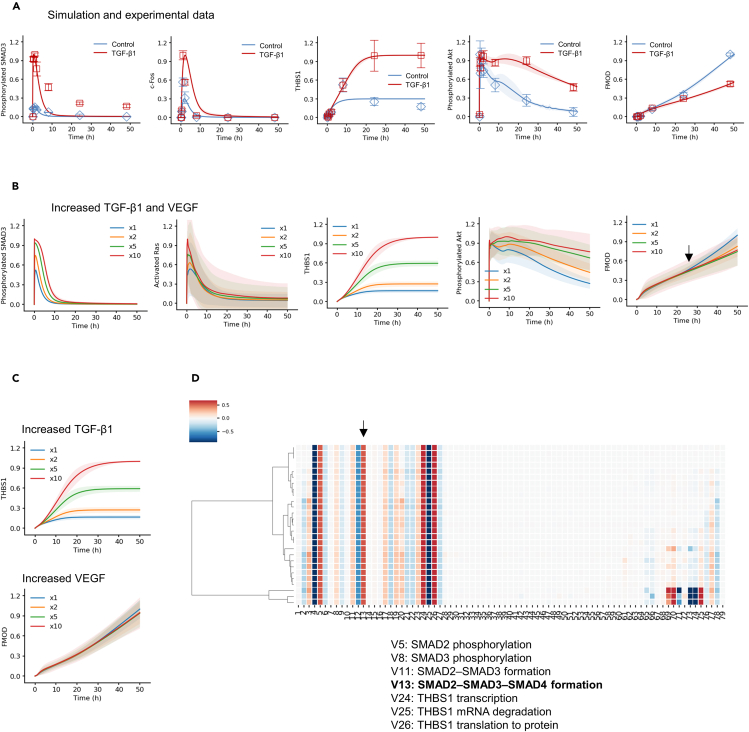
Experimental data-based mathematical modeling and simulation of skin aging (A) Model parameters for the integrated TGF-β–VEGF model were trained on time course phosphorylated SMAD3, c-Fos, THBS1, phosphorylated Akt (Ser473), and FMOD protein levels obtained with/without TGF-β1 treatment. The points (control: blue squares, TGF-β1: red squares) indicate experimental data, solid lines indicate the average simulation of 30 parameter sets, and shade areas indicate SD. Error bar represents SD; *N* = 3, mean ± SD. WB images are shown in Figure S12A. (B) Simulation of phosphorylated SMAD3, activated Ras (Ras-GTP), THBS1, FMOD, and phosphorylated Akt levels using integrated TGF-β–VEGF mathematical model. Initial values of both TGF-β1 and VEGF were increased, as indicated by the color code (from ×1 to ×10). Solid lines indicate the average simulation of 30 parameter sets, and shade areas indicate SD. The black arrow indicates breakpoint of FMOD inhibition. (C) Simulation of THBS1 and FMOD using developed mathematical model. The initial value of TGF-β1 for THBS1 or VEGF for FMOD were increased, as indicated by the color code (From ×1 to ×10). Solid lines indicate the average simulation of 30 parameter sets, and shade areas indicate SD. (D) Sensitivity analysis of TGF-β1-induced THBS1 for 30 parameter sets. Negative coefficients (blue) indicate that the quantity of the response metric decreases as species increase, while positive coefficients (red) indicate that the quantity of the metric increases. The black arrow indicates V13, SMAD2–SMAD3–SMAD4 formation.

Because of the limitations in explaining the molecular mechanisms using the aforementioned model near equilibrium state, we further constructed a comprehensive ODE model of the TGF-β and VEGF receptor signaling network to quantitatively understand the dynamics of skin aging regulated by THBS1 and FMOD (Figure S11). The integrated model included latent TGF-β1 activation by THBS1,^28^ inhibition by FMOD,^29^^,^^30^^,^^31^ SMAD-AP-1 complex formation,^45^^,^^46^^,^^47^ a negative feedback by SMAD7,^49^ positive feedback of THBS1,^28^^,^^50^^,^^51^^,^^52^ VEGFR, and Raf-ERK cascade,^24^^,^^53^ and PI3K-Akt crosstalk between the TGF-β and VEGF pathways. The model was constructed using parameters trained with time course data (i.e., 0 min, 15 min, 30 min, 60 min, 120 min, 8 h, 24 h, and 48 h) of protein phosphorylation or expression (i.e., phosphorylated SMAD3, c-Fos, THBS1, phosphorylated Akt, and FMOD) in HFF-1 cells stimulated with or without TGF-β1 (Figures 5A, image: S12A). We obtained 30 well-fitting parameter sets (Figure S12B) that reproduced the experimental data (see details in the STAR Methods section). The traces of 30 optimization runs and distribution of the objective function, i.e., sum of residual squares between simulation and experimental values, showed a uniform distribution, indicating robust parameter estimation results (Figures S12C and S12D). The resulting model had 79 rate equations, 83 species, and 194 parameters. To validate the model and assess the reproducibility of the model dynamics, we used phosphor-SMAD2 data, which was not used as training data (Figure S12E). Finally, we obtained a model that could successfully reproduce most of the experimental results for HFF-1 cells.

### Late inhibition of PI3K-Akt is crucial for FMOD downregulation by TGF-β1

As TGF-β1 and VEGF expression is known to increase during cellular senescence^54^^,^^55^ and in HFF-1 cells with higher PDL (Figures 2A and S12F), numerical simulations were conducted with the generated comprehensive ODE model to mimic these changes. In these simulations, the initial concentrations of TGF-β1 and VEGF were increased to represent a state of senescence (Figure 5B). As the input increased, the levels of phosphorylated SMAD3 and activated Ras (Ras-GTP) gradually increased. Interestingly, as senescence progressed, THBS1 gradually increased until 24 h, while FMOD started to decrease from the 24 h time point, as compared to the control. We also observed that while the basal condition induced transient activation (blue line) of phosphorylated Akt at early phase, higher initial values resulted in sustained Akt activation (red line). This is likely because as cellular senescence progresses, Akt activation switches from transient to sustained, and sustained Akt activation suppresses FMOD expression in the later stages of the dynamics. Based on the simulation of FMOD expression, the 24 h time point (black arrow) was identified as the breakpoint of FMOD inhibition by transient and sustained Akt activation. This suggests that the Akt activation observed in early phase does not affect FMOD expression and that FMOD recovery may be achieved by selectively inhibiting persistent Akt activity in the late phase.

**Figure 6:**
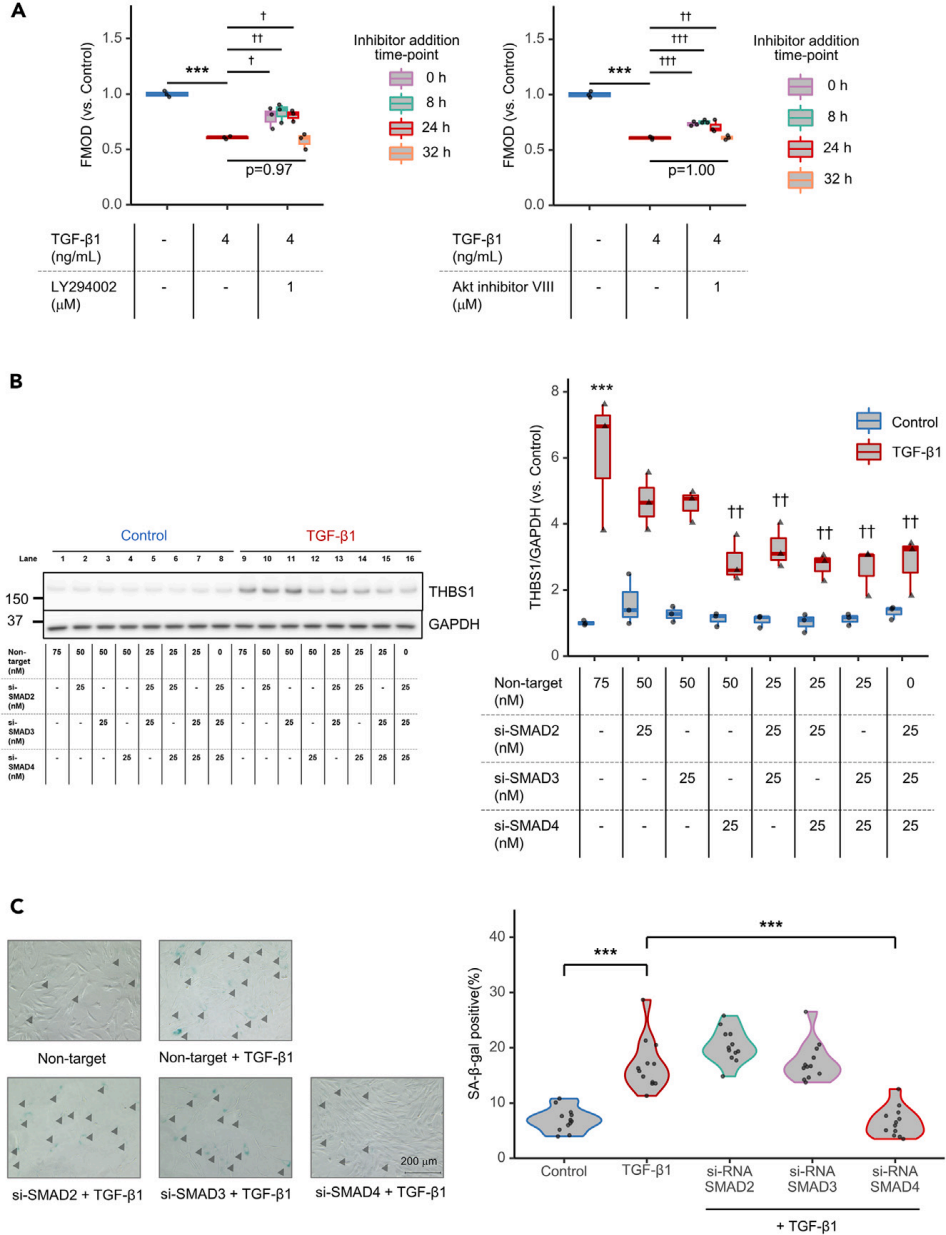
SMAD4 is a potential target for inhibiting THBS1 expression and cell senescence (A) Quantification of FMOD in LY294002- (left panel) or Akt inhibitor viii- (right panel) treated HFF-1 cells by ELISA. Cells were initially treated with 4 ng/mL TGF-β1, 1 μM LY294002, or 1 μM Akt inhibitor viii was added at certain time points (0 h, purple; 8 h, green; 24 h, bright red; 32 h, orange), and supernatants were collected at 48 h. The relative expression to the control (blue) is shown. TGF-β1 conditions that were not treated with inhibitors are indicated in dark red. The horizontal line in the center of the box plot is the median, the lower and upper borders indicate the 25th and 75th percentiles, and the whiskers extend to the minimum and maximum values, respectively. *N* = 3, ∗∗∗*p* < 0.01 (vs. control, Student’s t test), †*p* < 0.05, ††*p* < 0.01, †††*p* < 0.01 (vs. TGF-β1, Dunnett’s test). (B) Validation of the sensitivity analysis by WB analysis of THBS1 following siRNA KD of SMADs in HFF-1 cells. Lysates were collected 48 h after pretreatment with each SMAD siRNA alone or in combination for 24 h prior to stimulation with or without 4 ng/mL TGF-β1. (Left panel) Representative image. (Right panel) Quantification of THBS1 with SMADs KD; *N* = 3, ∗∗∗*p* < 0.001 (vs. non-target control treatment, Student’s t test), ^††^*p* < 0.01 (vs. non-target TGF-β1 treatment, Dunnett’s test). (C) Effect of siRNA KD of SMADs on SA-β-gal activity. HFF-1 cells were pretreated with each 25 nM siRNA and stimulated with or without 4 ng/mL TGF-β1. (Left panel) Representative images. SA-β-gal-positive cells are indicated with the arrowhead (black). Scale bars, 200 μm. See Figure S13A for processed images. (Right panel) Quantification of SA-β-gal: SA-β-gal-positive rate (%) = SA-β-gal-positive cells/total number of cells × 100. Four images per well were randomly analyzed from three wells (total 12 images/condition), ∗∗∗*p* < 0.001 (Tukey’s multiple comparisons).

To verify this difference in Akt activation, we examined FMOD recovery in TGF-β1-treated HFF-1 cells by adding LY294002 (or Akt inhibitor VIII) at specific time points to suppress Akt phosphorylation (Figure 6A). While FMOD expression was significantly reduced by TGF-β1 treatment alone, co-treatment with LY294002 (or Akt inhibitor VIII) from start to end (indicated as 0 h) restored FMOD expression. When the inhibitor was added at 8 h or 24 h, FMOD recovered to the same level as observed at 0 h. However, no recovery was observed after 32 h, which exceeds the 24 h breakpoint where FMOD begins to decrease. Thus, using model simulations and their validation, we have demonstrated that the sustained dynamics of Akt activity are critical for the negative regulation of FMOD expression in the progression of cellular senescence.

### THBS1 is a sensitive factor, FMOD is a robust factor, and SMAD2/3/4 formation is effective in suppressing THBS1 expression

Finally, we aimed to identify the target molecule regulating the rate of cellular senescence. Our findings thus far point to two possible targets: THBS1 can be downregulated or FMOD can be upregulated. To determine which target is more suitable for manipulation, we simulated THBS1 and FMOD expression as model outputs by increasing the input of TGF-β1 and VEGF, respectively (Figure 5C). Interestingly, the simulation showed that THBS1 expression was sensitive to TGF-β1 input, whereas FMOD was minimally affected by VEGF input. This prediction for VEGF was confirmed in an *in vitro* experiment with HFF-1 cells (Figure 3G). Indeed, the expression of FMOD was significantly increased with increasing VEGF concentrations, but the rate of increase was much lower than that for THBS1 induced by TGF-β1 (Figure 3A). These results suggest that THBS1 is a sensitive factor, while FMOD is a more robust factor in cell senescence.

To further identify the molecular factors regulating THBS1 activity, we performed a sensitivity analysis^56^ to determine the bottleneck of the TGF-β signaling network with THBS1 as output (Figure 5D). A reaction involving SMAD2SMAD3-SMAD4 complex formation (V13, black arrow) was shown to be more sensitive than reactions involving SMAD2 and SMAD3 alone. This finding suggests that inhibition of the SMAD4 reaction can effectively suppress THBS1 production. This simulation was validated by monitoring TGF-β1-induced THBS1 expression after siRNA KD of SMAD2, SMAD3, and SMAD4 in HFF-1 cells (Figure 6B). As suggested by the model, SMAD4 KD had the greatest effect on THBS1 expression compared to that of SMAD2 and SMAD3. Combinations of SMAD2-SMAD4, SMAD3-SMAD4, or SMAD2-SMAD3-SMAD4 KDs did not show synergistic or additive effects compared to SMAD4 KD alone. After KD of each SMAD, we found that SMAD4 KD significantly suppressed the induction of TGF-β1-induced SA-β-gal positivity compared to that by SMAD2 and SMAD3 KDs (Figures 6C and S13A). We also checked the effect of each SMAD KD on Lamin-B1—which was reduced by TGF-β1 treatment—to determine if SMAD4 KD is effective against other aging markers (Figure S13B). We found that SMAD4 (*p* < 0.01) KD significantly restored Lamin-B1 expression, whereas SMAD2 (*p* = 0.79) and SMAD3 (*p* = 0.72) did not. These results suggested that SMAD4 is essential for THBS1 induction and is a critical target for controlling cellular senescence.

## Discussion

In this study, we investigate the regulatory network in skin aging using multi-omics and mechanistic modeling approaches. A previous study showed that inflammation and fibrosis define senescence *in vivo.*^57^ Our data-driven analysis of skin aging has specifically highlighted the role of the TGF-β signaling pathway. TGF-β signaling pathway, which regulates fibrosis, in senescent cells can induce senescence in surrounding cells via TGF-β1;^55^^,^^58^ the transcription of p21 can be activated by the TGF-β signaling pathway;^59^ and a transcriptome analysis of aging mouse skin implicated the TGF-β pathway as a regulator.^60^ These results support TGF-β1 as a key upstream regulator of human skin aging. In addition to TGF-β1, our analysis identified an age-related enrichment of estrogen receptor 2 (ESR2). Knockout of the human *ESR2* gene reduced the expression of human *THBS1* mRNA^61^ and its ligand 17β-estradiol induced *THBS1* mRNA expression.^62^ 17β-estradiol decreased *Fmod* mRNA expression in the frontal cortex of rats.^63^ These results suggest that ESR2 may also be involved in the regulation of THBS1 and FMOD in dermal senescence. Furthermore, USP11 has been reported to stabilize p21 levels in a p53-independent manner.^32^ As p53 expression did not decrease with increasing PDL in our study, it is possible that changes in USP11 are responsible for the downregulation of p21 in the late PDL.

In previous reports, the analysis of human dermal fibroblast mRNA suggested that *THBS1* expression increased with age.^64^ Among 998 proteins that showed an age-dependent secretion pattern, THBS1 was upregulated with skin aging.^65^ These reports implicated THBS1 as a biomarker of skin aging but did not directly demonstrate its function. Collectively, our findings and previous findings suggest that THBS1 expression plays a vital role as a universal phenotype in skin aging. In addition, THBS1 was reported to promote senescence in endothelial cells.^66^^,^^67^ Given the strong relationship between THBS1 and age-associated diseases,^39^ our current findings on THBS1 have potential applications beyond skin aging. In contrast, the expression of FMOD seems to be heterogeneous among reports of skin aging. We found that the expression of FMOD decreased with the senescence of dermal fibroblasts. Proteomic analysis of human skin punch biopsies showed that FMOD protein expression decreased with age.^68^ In contrast, an earlier analysis of skin-fibroblast mRNA also suggested that FMOD increases with age.^64^ FMOD expression was upregulated with increasing age and senescence in the public RNA-seq dataset, which contradicts our validation. A possible explanation for this difference in the senescence tendency of FMODs in the same HFF-1 cell line could be the variation in culture conditions, such as the medium glucose level. Inconsistencies in reports on FMOD regulation may also be due to the differences between proteomic and transcriptomic data in aging, suggesting that their expressions are poorly correlated and that mRNA profiling alone does not provide the complete picture.^69^

Our findings suggest that both SMAD activation and c-Fos/c-Jun binding to DNA are required to regulate THBS1 expression in dermal fibroblasts. In earlier studies, THBS1 was regulated by the SMAD3 binding site of the THBS1 promoter;^52^ c-Jun, but not c-Fos, was involved in AP-1 activity at the AP-1 binding site of the THBS1 promoter in human hepatocarcinoma cell lines.^70^ Various TFs (e.g., NF-κB, USF, E2F1, AP-1, EGR1, and SP1) have been reported to bind to the promoter region of THBS1;^71^ therefore, the TFs associated with THBS1 may vary among cell lines. Meanwhile, FMOD expression in dermal fibroblasts was found to be regulated by the VEGF-Raf-ERK pathway. Previous reports have indicated that FMOD was regulated by the Wnt/β-catenin pathway in human breast cancer cell lines,^72^ MAPK/AP-1 pathway in human pancreatic stellate cells,^73^ and TGF-β2 in rat pericytes.^74^ These results suggest that FMOD regulation may vary in different cells, tissues, or environments.

Our bifurcation analysis of TGF-β1, THBS1, and FMOD network indicate a binary high-/low-TGF-β1 switch. Earlier studies identified a bistable switch that regulates TGF-β1 activation in liver fibrosis^75^ and asthmatic airways.^76^ In addition to these diseases, the bifurcation analysis may be useful in qualitatively capturing other phenomena of senescence.

Several mathematical models have been reported for the TGF-β signaling pathway.^77^^,^^78^^,^^79^ Nevertheless, our model is invaluable because it reflects unbiased data-driven insights into the gene regulatory network of skin aging, combined with the previous models. Computational simulation of skin aging showed that THBS1 responded sensitively while FMOD was robustly regulated with any input. These results suggest that THBS1 is a more promising drug target than FMOD. The sensitivity analysis further confirmed that the model-predicted sensitive response, i.e., the complex formation by SMAD4, was conserved across 30 independent parameter sets, suggesting that parameter identifiability does not affect the uncertainty of model outputs. In the siRNA-based SMADs KD experiments, knockdown of the SMAD2-SMAD3 combination (besides SMAD4) significantly reduced THBS1, which may indicate a functional redundancy of SMAD2 and SMAD3. On the contrary, when considering practical applications in drug development, focusing on a single target (SMAD4) is more suitable than developing drugs for two alternative targets, SMAD2 and SMAD3. Experimental evidence has shown that inhibition of SMAD4 complex formation has a restorative effect on the aging phenotype (i.e., SA-β-gal and lamin-B1), and that its effects go beyond THBS1 regulation. This approach of targeting THBS1 by inhibiting SMAD4 complex formation will open new avenues for skin aging research.

There is a growing demand for representative simulations of real-world disorders and diseases, and in that aspect, our study represents a considerable advance in the modeling of skin aging.

### Limitations of the study

The mechanisms regulating FMOD expression in the dermal fibroblasts still remain unclear. Although the involvement of VEGF pathway was demonstrated, this study does not rule out the possibility that FMOD expression may also be regulated by other ligands. In addition, we used an anonymous transcription factor for FMOD in our mathematical model. Furthermore, the role of endogenous TGF-β signaling in senescent transcripts is yet to be elucidated.

Further research is necessary to elucidate the factors regulating FMOD in skin aging, especially because the regulation of FMOD may vary in different cells and tissues, or in different environments.

## STAR★Methods

### Key resources table

**Table:** 

REAGENT or RESOURCE	SOURCE	IDENTIFIER
**Antibodies**		

Anti-THBS1	Cell Signaling Technology	Cat# 37879; RRID: AB_2799123
Anti-THBS1	Abcam	Cat# ab85762, RRID: AB_10674322
Anti-FMOD	ProteinTech	Cat# 60108-1-Ig; RRID: AB_2105538
Anti-Lamin B1	ProteinTech	Cat# 12987-1-AP; RRID: AB_2136290
Anti-p53	Cell Signaling Technology	Cat# 2524; RRID: AB_331743
Anti-p21	Cell Signaling Technology	Cat# 2946; RRID: AB_2260325
Anti-c-Fos	Cell Signaling Technology	Cat# 2250; RRID: AB_2247211
Anti-c-Jun	Cell Signaling Technology	Cat# 9165; RRID: AB_2130165
Anti-SMAD2	Cell Signaling Technology	Cat# 5339; RRID: AB_10626777
Anti-phosphorylated SMAD2 (Ser465/467)	Cell Signaling Technology	Cat# 3108; RRID: AB_490941
Anti-SMAD3	Cell Signaling Technology	Cat# 9523; RRID: AB_2193182
Anti-phosphorylated SMAD3 (Ser423/425)	Cell Signaling Technology	Cat# 9520; RRID: AB_2193207
Anti-SMAD4	Cell Signaling Technology	Cat# 46535; RRID: AB_2736998
Anti-phosphorylated Akt (Ser473)	Cell Signaling Technology	Cat# 9271; RRID: AB_329825
Anti-pan Akt	Cell Signaling Technology	Cat# 2920; RRID: AB_1147620
Anti-USP11	Abcam	Cat# ab109232; RRID: AB_10862711
Anti-GAPDH	Medical & Biological Laboratories	Cat# M171-3; RRID: AB_10597731
Anti-GAPDH	ProteinTech	Cat# 10494-1-Ap; RRID: AB_2263076
Anti-H3K27Ac	Abcam	Cat# ab177178; RRID: AB_2828007
Anti-IgG (H+L chain) (Mouse) pAb-HRP	Medical & Biological Laboratories	Cat# 330; RRID:AB_2650507
Anti-IgG (H+L chain) (Rabbit) pAb-HRP	Medical & Biological Laboratories	Cat# 458; RRID:AB_2827722
Goat Anti-Rabbit IgG H&L (Alexa Fluor® 594)	Abcam	Cat# ab150080, RRID:AB_2650602

**Chemicals, peptides, and recombinant proteins**		

D-PBS (-) (1X)	Nacalai Tesque	Cat# 14249-24
HBSS	Thermo Fisher Scientific	Cat# 14025092
Dulbecco’s modified Eagle’s medium	ATCC	Cat# 30-2002
Fetal bovine serum	Corning	Cat# 35-010-CV
Goat serum	Thermo Fisher Scientific	Cat# 16210–064
Antibiotic–antimycotic	Thermo Fisher Scientific	Cat# 15240062
Trypsin/EDTA	ATCC	Cat# 30-2101
BAMBANKER®	NIPPON Genetics	Cat# CS-04-001
RIPA Buffer	Thermo Fisher Scientific	Cat# 89900
Halt™ Protease and Phosphatase inhibitor	Thermo Fisher Scientific	Cat# 78442
Trypan blue	Thermo Fisher Scientific	Cat# 15250-061
Cell counter	WakenBtech	Cat# WC2-100
Hoechst® 33342	DOJINDO	Cat# 346-07951
Opti-MEM™ I	Thermo Fisher Scientific	Cat# 31985-070
Lipofectamine RNAiMax	Thermo Fisher Scientific	Cat# 13778150
Precast gel 7.5–15%	Nacalai Tesque	Cat# 13066-44
Wide Precast Gel 7.5–15%	Biocraft	Cat# MDG-287
Tris/Glycine/SDS Buffer	Bio-Rad	Cat# 1610732
4× Laemmli sample buffer	Bio-Rad	Cat# 1610747
2-Mercaptoethanol	Bio-Rad	Cat# 1610710
Precision Plus Protein WesternC blotting standard	Bio-Rad	Cat# 1610376
Clarity Western ECL Substrate	Bio-Rad	Cat# 1705060
Clarity Max Western ECL Substrate	Bio-Rad	Cat# 1705062
Blocking buffer	Bio-Rad	Cat# 12010020
iBlot®2 PVDF	Thermo Fisher Scientific	Cat# IB24001
Reconstitution buffer (0.1% BSA in 4 mM HCl PBS)	R&D Systems	Cat# RB04
Recombinant human TGF-β1	R&D Systems	Cat# 7754-BH-005
Recombinant human TGF-β2	ProteinTech	Cat# HZ-1092
Recombinant human TGF-β3	ProteinTech	Cat# HZ-1090
Recombinant human EGF	PeproTech	Cat# AF-100-15
Recombinant human b-FGF	R&D Systems	Cat# 233-FB-025
Recombinant human PDGF-BB	ProteinTech	Cat# HZ-1308
Recombinant human THBS1	R&D Systems	Cat# 3074-TH-050
Recombinant human FMOD	Abcam	Cat# ab152392
Recombinant human VEGF165	ProteinTech	Cat# HZ-1038
Dimethyl sulfoxide	Fujifilm	Cat# 041-29351
LY364947	Fujifilm	Cat# 123-05981
Akt inhibitor VIII	Cayman Chemical	Cat# 14870
LY294002	Calbiochem	Cat# 440202
T-5224	Selleck Chemicals	Cat# S8966
Tocriscreen Kinase Inhibitor Toolbox	Tocris Bioscience	Cat# 3514
ZM306416HCl	Tocris Bioscience	Cat# 3514-2499
Ki8751	Tocris Bioscience	Cat# 3514-2542
GW5074	Tocris Bioscience	Cat# 3514-1381
U0126	Tocris Bioscience	Cat# 3514-1144
SB 431542	Tocris Bioscience	Cat# 3514-1614
Formaldehyde	Thermo Fisher Scientific	Cat# 28908
Paraformaldehyde	Electron Microscopy Science	Cat # 15710
HCS CellMask™ Stains	Thermo Fisher Scientific	Cat# H32721
Triton® X-100	NACALAI TESQUE	Cat# 35501-15
Proteinase K	Thermo Fisher Scientific	Cat# 26160
Dispase® II	Roche	Cat# 04942078001

**Critical commercial assays**		

Pierce™ BCA Protein Assay Kit	Thermo Fisher Scientific	Cat# 23227
NucleoSpin® RNA kit	Macherey-Nagel GmbH & Co.	Cat# 740955
ReverTra Ace® qPCR RT Master Mix	Toyobo Life Science	Cat# FSQ-201
KOD SYBR® qPCR kit	Toyobo Life Science	Cat# QKD-201
SimpleChIP® Enzymatic Chromatin IP kit	Cell Signaling Technology	Cat# 9003
iDeal ChIP-seq kit for Histones	Diagenode	Cat# C01010171
MinElute® PCR Purification Kit	Qiagen	Cat# 28004
Bioanalyzer Agilent High-Sensibility DNA Kit	Agilent	Cat# 5067-4626
NEBNext® Poly(A) mRNA Magnetic Isolation Module	New England Biolabs	Cat# E7490
NEBNext® Ultra™ ll Directional RNA Library Prep Kit	New England Biolabs	Cat# E7760
ATAC-Seq Kit	Active Motif	Cat# 53150
NEBNext® Ultra II DNA Library Prep Kit for Illumina	New England Biolabs	Cat# 7645
Cell Counting Kit-8	DOJINDO	Cat# 343-07623
SA-β-gal Detection Kit	BioVision	Cat# K320-250
CycLex® Cellular BrdU ELISA Kit Ver.2	Medical & Biological Laboratories	Cat# CY-1142V2
THBS1 ELISA Kit	R&D Systems	Cat# DTSP10
FMOD ELISA Kit	Abcam	Cat# ab275895
TGF-β1 ELISA Kit	R&D Systems	Cat# DB100B
VEGF ELISA Kit	R&D Systems	Cat# DVE00
SMAD2 ELISA Kit	Abcam	Cat# ab260065
SMAD3 ELISA Kit	Abcam	Cat# ab264624
SMAD4 ELISA Kit	Abcam	Cat# ab253211
IL-6 ELISA Kit	R&D Systems	Cat# D6050
IL-8 ELISA Kit	Abcam	Cat# ab214030

**Deposited data**		

RNA-seq: HFF-1 (PDL 24, PDL 36, PDL 47) Treatment: Control	This Paper	DDBJ: DRA016119 BioProject: PRJDB15707
RNA-seq: HFF-1 (PDL 24, PDL 36, PDL 47) Treatment: 4 ng/mL TGF-β1	This Paper	DDBJ: DRA017188 BioProject: PRJDB15707
ChIP-seq: H3K27Ac HFF-1 (PDL 24, PDL 36, PDL 47); Input HFF-1 (PDL 24, PDL 36, PDL 47) Treatment: Control	This Paper	DDBJ: DRA016119 BioProject: PRJDB15707
ATAC-seq: HFF-1 (PDL 24, PDL 36, PDL 47) Treatment: Control	This Paper	DDBJ: DRA016119 BioProject: PRJDB15707
ATAC-seq: HFF-1 (PDL 24) Treatment: 4 ng/mL TGF-β1	This Paper	DDBJ: DRA017188 BioProject: PRJDB15707
RNA-seq: HFF-1 (PDL 24) initial condition	This Paper	DDBJ: DRA016119 BioProject: PRJDB15707
The code for bioinformatics analysis and mathematical modeling	This Paper	https://github.com/okadalabipr/Haga2023

**Experimental models: Cell lines**		

Human dermal fibroblast HFF-1	ATCC	Cat# SCRC-1041; RRID: CVCL_3285
Human dermal fibroblast BJ	ATCC	Cat# CRL-2522; RRID: CVCL_3653

**Experimental models: Organisms/Strains**		

Frozen human full thickness skin	Biopredic International	Cat# TRA1FTR0, TRA1FTR2

**Oligonucleotides**		

qRT-PCR forward primer: *THBS1* 5′-TCCCCATCCAAAGCGTCTTC-3′	This paper	N/A
qRT-PCR reverse primer: *THBS1* 5 ′-ACCACGTTGTTGTCAAGGGT-3′	This paper	N/A
qRT-PCR forward primer: *FMOD* 5′-GGACGTGGTCACTCTCTGAA-3′	This paper	N/A
qRT-PCR reverse primer: *FMOD* 5′-GGCTCGTAGGTCTCATACGG-3′	This paper	N/A
qRT-PCR forward primer: *GAPDH* 5′-GTCTCCTCTGACTTCAACAGCG-3′	OriGene	Cat# HP205798
qRT-PCR reverse primer: *GAPDH* 5′-ACCACCCTGTTGCTGTAGCCAA-3′	OriGene	Cat# HP205798
ON-TARGET plus Non-targeting siRNA	Dharmacon	Cat# D-001810-02-20
ON-TARGET plus Human c-Fos siRNA - SMARTpool	Dharmacon	Cat# L-003265-00-0010
ON-TARGET plus Human c-Jun siRNA - SMARTpool	Dharmacon	Cat# L-003268-00-0010
ON-TARGET plus Human SMAD2 siRNA - SMARTpool	Dharmacon	Cat# L-003561-00-0020
ON-TARGET plus Human SMAD3 siRNA - SMARTpool	Dharmacon	Cat# L-020067-00-0020
ON-TARGET plus Human SMAD4 siRNA - SMARTpool	Dharmacon	Cat# L-003902-00-0020

**Software and algorithms**		

CellProfiler ver. 4.2.1	Stirling et al.^84^	https://cellprofiler.org/ RRID: SCR_007358
ImageJ Fiji version 1.52p	Schindelin et al.^85^	http://fiji.sc RRID: SCR_002285
Biomass version 0.5.2	Imoto et al.^24^^,^^53^	https://github.com/biomass-dev/biomass RRID: N/A
Gnuplot vesion 5.4	Williams et al.^86^	http://www.gnuplot.info/ RRID: SCR_008619
R version 4.2.1	The R Foundation	https://r-project.org RRID: SCR_001905
QIAGEN Ingenuity Pathway Analysis version 81348237	Krämer et al.^87^	https://digitalinsights.qiagen.com/products-overview/discovery-insights-portfolio/analysis-and-visualization/qiagen-ipa/ RRID: SCR_008653
Nextflow version 21.10.6	Tommaso et al.^88^	https://github.com/nextflow-io/nextflow RRID: SCR_024135
nfcore/chipseq version 1.2.2	Ewels et al.^89^	https://nf-co.re/chipseq/1.2.2 https://zenodo.org/record/7139814#.Y4lJDXbP1aQ RRID: N/A
nfcore/atacseq version 1.2.1	Ewels et al.^89^	https://nf-co.re/atacseq/1.2.1 https://zenodo.org/record/7384115#.Y4lJv3bP1aQ RRID: N/A
Trim Galore! version 0.6.6	The Babraham Institute	http://www.bioinformatics.babraham.ac.uk/projects/trim_galore/ RRID: SCR_011847
hisat2 version 2.2.1	Kim et al.^90^	http://ccb.jhu.edu/software/hisat2/index.shtml RRID: SCR_015530
Samtools version 1.9	Danecek et al.^91^	https://github.com/samtools/samtools RRID: SCR_002105
Subread version 2.0.1	Liao et al.^92^	https://subread.sourceforge.net/ RRID: SCR_009803
BEDTools version 2.30.0	Quinlan et al.^93^	https://github.com/arq5x/bedtools2 RRID: SCR_006646
HOMER version 4.11	Heinz et al.^37^	http://homer.ucsd.edu/homer/ RRID: SCR_010881
DoRothEA version 1.8.0	Garcia-Alonso et al.^33^	https://saezlab.github.io/dorothea/ RRID: N/A
clusterProfiler version 4.4.4	Wu et al.^94^	https://bioconductor.org/packages/release/bioc/html/clusterProfiler.html RRID: SCR_016884
DESeq2 version 1.36.0	Love et al.^95^	https://bioconductor.org/packages/release/bioc/html/DESeq2.html RRID: SCR_015687
ChIPseeker version 1.32.1	Yu et al.^96^	https://bioconductor.org/packages/release/bioc/html/ChIPseeker.html RRID: SCR_021322
rrcov version 1.5.2	Hubert et al.^97^	https://cran.r-project.org/package=rrcov RRID: N/A

### Resource availability

#### Lead contact

Requests for raw data and code should be directed to and will be fulfilled by the lead contact, Mariko Okada (mokada@protein.osaka-u.ac.jp).

#### Materials availability

This study did not generate new unique reagents.

#### Data and code availability

All sequence data have been deposited in the DNA Data Bank of Japan (DDBJ) and is available under accession number PRJDB15707 (DDBJ: DRA016119, DRA017188). The code for bioinformatics analysis and mathematical modeling is available from GitHub (https://github.com/okadalabipr/Haga2023). Any additional information required to reanalyze the data reported in this paper is available from the lead contact upon request.

### Experimental model and study participant details

#### Cell lines

HFF-1 and BJ cells, human dermal fibroblasts, both derived from normal foreskin tissue of neonatal males, were purchased from the American Type Culture Collection (ATCC). HFF-1 and BJ cells were maintained using Dulbecco’s modified Eagle’s medium (DMEM, ATCC) supplemented with 10% fetal bovine serum (FBS, Corning) and 1% antibiotic–antimycotic (Thermo Fisher Scientific). Unless otherwise noted, the PDL of HFF-1 cells in the following experiments was kept at approximately 24, and that for BJ cells was approximately 36, which correspond with young cells. In the experiments, the cells were serum starved with 0.1% FBS and tested under uniform 2% FBS conditions during stimulation. All cell lines were maintained at 37°C in a humidified atmosphere at 5% CO_2_.

#### Human dermal tissue

Human full-thickness dermal tissues were purchased from Biopredic International via KAC and stored frozen at –20°C. Skin samples were anonymized and informed consent was obtained from all participants (see Table S2 for donor list). This study using human tissue samples was approved by the Ethics Committee of the Institute for Protein Research, Osaka University (clearance no. 2021-2).

### Method details

#### Induction of replication stress in dermal fibroblasts

Replication stress was induced by passage culturing of HFF-1 and BJ cells. Briefly, cells were seeded in a cell culture flask and grown on DMEM supplemented with 10% FBS until reaching a sub-confluent condition. The experiment was performed in three separate flasks. After removing the medium, washing with PBS, and detaching using trypsin/EDTA (ATCC), cells were frozen in BAMBANKER® solution (NIPPON Genetics) at –80°C in a BICELL container (Nihon Freezer) and then stored under liquid nitrogen. The PDL for each collection was calculated as follows: n=3.32 (log A–log B) + X (n: final PDL of cell line, A: yield of harvested cells, B: count of seeded cells, X: initial PDL of the seeded cell population).

#### Sample preparation for RNA-seq and genomic alignment

HFF-1 cells (PDL 24, PDL 36, and PDL 47) were used to prepare RNA-seq libraries. Briefly, each PDL was seeded in 6-well plates at 200,000 cells/well, treated with either control (DMEM with vehicle supplemented with 2% FBS) or 4 ng/mL TGF-β1 (R&D Systems), and total RNA (three wells per sample) was harvested after 48 h using a NucleoSpin RNA kit (Macherey-Nagel GmbH & Co.). The quality of the total RNA was evaluated using a 2100 Bioanalyzer (Agilent) and RNA samples with RNA integrity > 9.0 were used for library preparation. cDNA libraries were prepared using a NEBNext® Poly(A) mRNA Magnetic Isolation Module (New England Biolabs) for PolyA selection and NEBNext® Ultra™ ll Directional RNA Library Prep Kit (New England Biolabs). Samples were prepared according to the manufacturer’s protocol. The RNA-seq data were generated as paired-end 150 base reads on a NovaSeq 6000 (Illumina). The expression of specific genes was validated by qRT-PCR.

All RNA-seq data, including public RNA-seq data, were trimmed using Trim Galore ! version 0.6.6 and aligned to human reference genome GRCh38 using hisat2 version 2.2.1. Mapped reads were extracted using samtools version 1.9 and a read count matrix was created using gene annotation (GRCh38.p13) with Subread version 2.0.1 for downstream analysis.

#### Sample preparation for ChIP-seq and genomic alignment

HFF-1 cells (PDL 24, PDL 36, and PDL 47) were used to prepare ChIP-seq libraries. Briefly, cells for each PDL were seeded in a 145 mm dish at 4,000,000 cells per dish. After 48 h of incubation, the cells were collected using a SimpleChIP Enzymatic Chromatin IP kit (Cell Signaling Technology [CST]) to obtain sheared chromatin. Briefly, two dishes per sample were fixed with fresh 1% formaldehyde (Thermo Fisher Scientific) for 5 min. The cells were subjected to a micrococcal nuclease treatment at 37°C for 20 min and M220 Focused-ultrasonicator (Covaris) for 10 min to obtain chromatin in 150–900 bp DNA/protein fragments. Next, H3K27Ac antibody (Abcam) and an iDeal ChIP-seq kit for Histones (Diagenode) were used to obtain ChIP-DNA. Using an SX-8G (Diagenode) platform, 2 μg anti-H3K27Ac antibody was incubated with 10 μL DiaMag ProteinA-coated magnetic beads for 3 h and an IP reaction was performed for 12 h. Decrosslinking was performed using NaCl and Proteinase K (Thermo Fisher Scientific) at 60°C for 4 h. The purification of ChIP-DNA was conducted using a MinElute® PCR Purification Kit (Qiagen). The ChIP-seq libraries were prepared using a NEBNext® Ultra II DNA Library Prep Kit for Illumina (New England Biolabs). All samples were prepared according to the manufacturer’s protocol. ChIP-seq data were generated as paired-end 150 base reads on the NovaSeq 6000 (Illumina).

All ChIP-seq data were analyzed using the nfcore/chipseq pipeline version 1.2.2 with “nextflow run nf-core/chipseq -r 1.2.2 -profile singularity –input samplesheet_ChIP.csv --genome GRCh38 --save_reference --max_cpus 16 --max_memory 256.GB.” Briefly, reads were trimmed using Trim Galore! and aligned against the GRCh38 reference genome using BWA. MACS2 was used for broadPeak calling. Consensus peak sets across all samples were created using BEDTools version 2.30.0. The counts for consensus peaks were generated using FeatureCounts and differential chromatin accessibility was analyzed using DESeq2 version 1.36.0. For more details, see the nf-core/chipseq pipeline (https://nf-co.re/chipseq/1.2.2).

#### Sample preparation for ATAC-seq and genomic alignment

HFF-1 cells (PDL 24, PDL 36, and PDL 47) were used to prepare ATAC-seq libraries. Cells for each PDL were seeded in 6-well plates at 200,000 cells/well. After treatment with either control (DMEM with vehicle supplemented with 2% FBS) or 4 ng/mL TGF-β1 (R&D Systems), the cells were washed with PBS and detached using trypsin/EDTA after 48 h. Using 200,000 cells of each PDL, ATAC-seq libraries were constructed using an ATAC-Seq Kit (Active Motif) according to the manufacturer’s protocol. The ATAC-seq data were generated as paired-end 150 base reads on the NovaSeq 6000 (Illumina).

All ATAC-seq data were analyzed using the nfcore/atacseq pipeline version 1.2.1 with default parameters using the “nextflow run nf-core/atacseq -r 1.2.1 -profile singularity –input samplesheet_ATAC.csv --genome GRCh38 --save_reference --max_cpus 16 --max_memory 256.GB” arguments. The downstream procedure is similar to that for the ChIP-seq analysis (for more detail, see the nf-core/atacseq pipeline [https://nf-co.re/atacseq/1.2.1]).

#### Transcription factor enrichment

The activity of the top 20 TFs was determined for the DEGs according to the FC value for RNA-seq data between PDLs and normalized transcripts per million (TPM) value for RNA-seq data using DoRothEA version 1.8.0. First, to identify the DEGs in replication-stress induced HFF-1 cells, the FC and adj-p were calculated for PDL 24 vs. PDL 36, PDL 24 vs. PDL 47, and PDL 36 vs. PDL 47 (DEGs: |FC| > 1.2, adj-p<0.05) using DESeq2. DEGs and FC values were used as input to identify the top 20 TFs regulating the DEGs using DoRothEA (confidence level A, B, and C). The top 20 TFs were identified by extracting the top 10 TFs for PDL 24 vs. PDL 36, PDL 24 vs. PDL 47, and PDL 36 vs. PDL 47, excluding duplicate TFs. Finally, a normalized enrichment score (based on DoRothEA) was calculated for the top 20 TFs using normalized TPM values for all genes greater than five.

#### Motif analysis

Motif analysis was performed using HOMER version 4.11. Briefly, we first calculated the gain peak region of ATAC-seq from the counts at the consensus peak using DESeq2. The region with log_2_FC > 0 and adj-p<0.05 was defined as the gain peak that significantly increases with increasing PDL. Next, the gained peaks for PDL 24 vs. PDL 36, PDL 24 vs. PDL 47, and PDL 36 vs. PDL 47 were concatenated using the “cat” command, sorted and merged using “sortBed” and “mergeBed,” respectively, in Bedtools version 2.30.0. The concatenated gained peak bed file was analyzed with “findMotifsGenome.pl hg38 -size given -p 8” in HOMER to output log(adj-p value) and percent of target sequences with motif from knownResults.html.

#### Gene annotation of ATAC-seq and ChIP-seq peaks and intersection with DEGs

Peak annotation for ATAC-seq and ChIP-seq was performed using “annotatePeak” in the R package ChIPseeker version 1.32.1. First, the region with |log2FC| > 0 and adj-p<0.05 was defined as the differential peak that changes significantly for PDL 24 vs. PDL 36, PDL 24 vs. PDL 47, and PDL 36 vs. PDL 47 using DESeq2. Next, differential peaks were concatenated using the “cat” command, sorted and merged using “sortBed” and “mergeBed” in Bedtools. The differential peaks were annotated as the nearest neighboring gene with the closest distance from the peak to the transcription start site (TSS). The TSS region occurred from –3kb to +3kb. The annotation package for hg38 (TxDb.Hsapiens.UCSC.hg38.knownGene) was used as the TxDb object.^80^ Peak annotation was conducted with the “tssRegion=c (-3000, 3000), TxDb=TxDb.Hsapiens.UCSC.hg38.knownGene, annoDb=‘org.Hs.eg.db’” option. DEGs from the RNA-seq data were identified for PDL 24 vs. PDL 36, PDL 24 vs. PDL 47, and PDL 36 vs. PDL 47 using DESeq2 (DEGs: |FC| > 1.2, adj-p<0.05). Venn diagrams were created using DEGs and annotated genes from differential peaks in both the ATAC-seq and ChIP-seq data.

#### Ingenuity Pathway Analysis (IPA)

IPA was performed for upstream analysis using IPA tool version 81348237 software. The gene set was mapped to Ingenuity Knowledge Base using “core analysis.” Molecule type, genes, RNAs, and proteins were used for the upstream analysis. A right-tailed Fisher’s exact test was used to calculate a p-value of overlap determining the probability that the association between the gene set and the upstream regulators is explained by chance alone. The Benjamini–Hochberg (BH) method for multiple testing was used to calculate the adjusted p-value.

#### Analysis of public *in vivo* and *in vitro* RNA-seq data

All public RNA-seq data used the same genomic alignment and read count matrix pipeline as described in the “Sample preparation for RNA-seq and genomic alignment”.

*In vivo* RNA-seq data (GSE113957) of human arm skin was analyzed after filtering outliers and clustering. Since multiple ethnic and tissue sources were included, data for 55 samples were manually selected for skin fibroblasts derived from Caucasian individuals (11–71 years of age, see donor list in Table S1). The samples were manually divided into three clusters according to age (young: 10s to 20s; middle: 30s to 50s; aged: 60s to 70s). For each cluster, a robust principal component analysis (PCA) was performed using “rrcov” version 1.5.2 to detect outliers, resulting in a final dataset with 45 samples. Spearman’s correlation coefficient was calculated to evaluate the distance between each sample using normalized TPM values: distance =1 – Spearman’s correlation coefficient. Using the distance calculated, samples were clustered using the “hclust” function in R with “distance, method=‘ward.D2’” (Figures S2B and S2C). Finally, DEGs were identified by comparing each cluster using DESeq2 (DEGs: |FC| > 1.5, adj-p<0.05). The BH method was used to calculate the adjusted p-value.

*In vitro* RNA-seq data (GSE63577) for the human dermal fibroblast HFF-1 cell line was compared for each PDL (PDL 16, PDL 26, PDL 46, PDL 64, and PDL 74). DEGs were identified for PDL 16 vs. PDL 26, PDL 16 vs. PDL 46, PDL 16 vs. PDL 64, PDL 16 vs. PDL 74, PDL 26 vs. PDL 46, PDL 26 vs. PDL 64, PDL 26 vs. PDL 74, PDL 46 vs. PDL 64, PDL 46 vs. PDL 74, and PDL 64 vs. PDL 74 using DESeq2 (DEGs: |FC| > 2.0, adj-p<0.01) and intersected. The BH method was used to calculate the adjusted p-value.

RNA-seq data for skin fibroblasts from healthy subjects (age: 1–9 years, N=12) and Hutchinson–Gilford progeria syndrome (HGPS) patients (age: 2–8 years, N=10) were downloaded from GSE113957 (see donor list in Table S3).

#### Kyoto Encyclopedia of Genes and Genomes (KEGG) analysis of public RNA-seq

KEGG enrichment was compared using the “comparecluster” function in the R package clusterProfiler version 4.4.4 with “fun”=enrichKEGG, “organism”=hsa, “keyType”=kegg, “pAdjustMethod”=BH, “minGSSize”=10, “maxGSSize”=500, “pvalueCutoff”=0.05, “qvalueCutoff”=0.05, and “use_internal_data”=“FALSE”. The BH method was used to calculate the adjusted p-value.

#### Correlation analysis of TGF-β pathway-enriched genes using public RNA-seq data

Spearman’s correlation between age and gene expression was calculated for the *in vivo* data or PDL and gene expression for the *in vitro* data using “cor” (method=“spearman”) in R. The genes associated with KEGG term “TGF-beta signaling pathway” in Figure 1H are plotted.

#### Integrated analysis of ATAC-seq and RNA-seq of TGF-β1 treatment

Peak annotation for ATAC-seq was performed using “annotatePeak” in the R package ChIPseeker version 1.32.1. Briefly, the region with adj-p<0.05 was defined as the differential peak that changes significantly for control vs. 4 ng/mL TGF-β1 using DESeq2. The differential peaks were annotated as the nearest neighboring gene with the closest distance from the peak to the TSS. The TSS region occurred from –3kb to +3kb. The annotation package for hg38 (TxDb.Hsapiens.UCSC.hg38.knownGene) was used as the TxDb object.^80^ Peak annotation was conducted with the “tssRegion=c (-3000, 3000), TxDb=TxDb.Hsapiens.UCSC.hg38.knownGene, annoDb=‘org.Hs.eg.db’” option. DEGs from the RNA-seq data were identified for control vs. 4 ng/mL TGF-β1 using DESeq2 (adj-p<0.05).

#### Extraction of human skin tissue protein

Skin tissue proteins were extracted after dividing full thickness skin into dermis and epidermis.^81^ In detail, a biopsy punch (tip diameter: 3 mm ø) was used to collect a full-layer skin sample, from which subcutaneous tissue was physically removed. After washing in 70% ethanol and HBSS (Thermo Fisher Scientific), samples were treated with 25 UI/mL Dispase II (Roche) HBSS solution for 15 h at 4°C. Enzymatic digestion was inactivated with HBSS supplemented with 10% FBS and the dermis and epidermis were separated with tweezers. Each dermis and epidermis were separately homogenized in RIPA buffer (Thermo Fisher Scientific) supplemented with Halt™ Protease and Phosphatase Inhibitor (Thermo Fisher Scientific) for 10 min using a Powermasher II (Nippi) and centrifuged (13,000 rpm, 20 min, 4°C) to generate protein extracts for western blot (WB) analysis.

#### Time-course cell proliferation analysis (trypan blue)

HFF-1 cells were seeded in 6-well plates with DMEM at 200,000 cells/well. After serum starvation for 16 h, cells were treated with either control (DMEM with vehicle supplemented with 2% FBS) or 4 ng/mL TGF-β1 (R&D Systems) and collected at 0 h, 8 h, 12 h, 24 h, and 48 h. Reconstitution buffer (0.1% bovine serum albumin [BSA] in 4 mM HCl PBS; R&D Systems) was used as vehicle and to dissolve TGF-β1. At each time-point, cells were washed with PBS and detached using trypsin/EDTA to count cells positive for trypan blue (Thermo Fisher Scientific) using a cell counter (WakenBtech) according to the manufacturer’s protocol.

#### Cell viability analysis (WST-8)

HFF-1 cells were seeded in 96-well plates with DMEM at 10,000 cells/well. After serum starvation for 16 h, cells were treated with each treatment. After 24 h, 10 μL Cell Counting Kit-8 solution (DOJINDO) was added, incubated for 1 h, and cell viability was calculated at an absorbance wavelength of 450 nm using a Multiskan FC system (Thermo Fisher Scientific).

#### 5-Bromo-2-deoxyuridine (BrdU) incorporation assay

The inhibition of DNA synthesis was measured using a CycLex Cellular BrdU ELISA Kit Ver.2 (Medical & Biological Laboratories) according to the manufacturer’s instructions. Briefly, HFF-1 cells were seeded in 96-well plates with DMEM at 10,000 cells/well. After 16 h of serum starvation, cells were treated with either control (DMEM with vehicle supplemented with 2% FBS), 4 ng/mL TGF-β1 (R&D Systems), or 1 μg/mL THBS1 (R&D Systems). After 8 h, the cells were incubated with anti-BrdU antibody and substrate for 16 h, and absorbance was measured at 450 nm with 540 nm as a reference using the Multiskan FC system (Thermo Fisher Scientific).

#### SA-β-gal staining and cell count (Hoechst® 33342)

The rate of positive SA-β-gal staining was calculated using a SA-β-gal Detection Kit (BioVision) according to the manufacturer’s protocol. Briefly, cells were seeded in 12-well plates at 100,000 cells/well, treated with each stimulant (N=3), and fixed for 10 min with fixative solution after 48 h. The cells were stained with Staining Solution Mix at 37°C overnight. After washing with PBS, cells were treated with Hoechst® 33342 (DOJINDO) for 10 min and washed with PBS before observation (BZ-9000, KEYENCE). SA-β-gal-positive rates were calculated as follows: SA-β-gal-positive rate (%) =number of SA-β-gal-positive cells per image / total number of cells per image × 100. For each treatment, four images per well were randomly analyzed from three wells (total 12 images/treatment). The number of SA-β-gal-positive cells was determined using thresholds for each experiment using the ImageJ Fiji package (The National Institutes of Health). All image files were split into red, blue, and green channels. The red channels were subtracted from the green channels and a threshold of (20, 30) was set to identify SA-β-gal positive cells. The total number of cells per image was also counted as Hoechst® 33342-positive cells using the ImageJ Fiji package.

#### siRNA transfection

HFF-1 cells were transfected with siRNA oligomer (Dharmacon/Horizon Discovery) mixed with Lipofectamine RNAiMAX reagent (Thermo Fisher Scientific) in Opti-MEM™ I (Thermo Fisher Scientific) according to the manufacturer’s instructions. Briefly, cells were seeded in 6-well plates with DMEM at 200,000 cells/well. After transfection for 24 h in serum-starved conditions, cells were treated with either control (DMEM with vehicle supplemented with 2% FBS) or 4 ng/mL TGF-β1 (R&D Systems) and the lysates or its supernatants were collected for further quantification. KD efficacy of c-Fos and c-Jun at 50 nM siRNA are shown in Figure S5B. In addition, KD efficacy at 48 h post-stimulation of SMAD2, SMAD3, and SMAD4 at 25 nM siRNA is shown in Figure S13B.

#### Inhibitor screening for FMOD regulatory pathway

HFF-1 cells were seeded in 96-well plates with DMEM at 10,000 cells/well. Cells were treated with control (DMEM with 2% FBS-added vehicle) or 4 ng/mL TGF-β1(R&D Systems) with or without each inhibitor, and supernatants were collected for FMOD ELISA. The following inhibitors were used: Akt inhibitor VIII (14870, Cayman Chemical) and LY294002 (440202, Calbiochem). The other inhibitors used were dispensed from a Tocriscreen Kinase Inhibitor Toolbox (3514, Tocris Bioscience).

#### Inhibitor treatment for THBS1 regulatory pathway

HFF-1 cells were seeded in 6-well plates with DMEM at 200,000 cells/well. Cells were treated with control (DMEM with 2% FBS-added vehicle) or 4 ng/mL TGF-β1 (R&D Systems) with or without each inhibitor, and the lysates were collected after 48 h for quantification by WB. The following inhibitors were used: LY364947 (123-05981, Fujifilm), SB431542 (dispensed from a Tocriscreen Kinase Inhibitor Toolbox [3514, Tocris Bioscience]), and T-5224 (S28966, Selleck).

#### Bifurcation model analysis

Based on the experimental expression of THBS1 and FMOD upon treatment with different concentrations of TGF-β1 (Figure 3A), we constructed a core transcription factor network consisting of TGF-β1, THBS1, and FMOD. A double positive feedback loop (between THBS1 and TGF-β1) and double negative feedback loop (between FMOD and TGF-β1) can be described by the following nonlinear ordinary differential equations (ODEs):

(Equation 1) $$\begin{array}{c} \frac{d\left[\text{THBS}1\right]}{dt}=a\left(\frac{{\left[\text{TGF}\beta 1\right]}^{n_{a}}}{{\left[\text{TGF}\beta 1\right]}^{n_{a}}+K_{a}}\right)-d_{1}\left[\text{THBS}1\right]\text{,} \end{array}$$

(Equation 2) $$\begin{array}{c} \frac{d\left[\text{FMOD}\right]}{dt}=b\left(\frac{K_{b}}{{\left[\text{TGF}\beta 1\right]}^{n_{b}}+K_{b}}\right)-d_{2}\left[\text{FMOD}\right]\text{,} \end{array}$$

(Equation 3) $$\begin{array}{c} \frac{d\left[\text{TGF}\beta 1\right]}{dt}=c\left(\frac{\left[\text{THBS}1\right]\;}{\left[\text{THBS}1\right]+K_{1}}\right)\left(\frac{K_{2}}{\left[\text{FMOD}\right]+K_{2}}\right)-d_{3}\left[\text{TGF}\beta 1\right]\text{,} \end{array}$$

where $a/d_{1}$, $b/d_{2}$, and $c/d_{3}$ are maximal values of [THBS1], [FMOD], and [TGFβ1], respectively; $K_{a}$, $K_{b}$, $K_{1}$, and $K_{2}$ are the half saturation constants; and $n_{a}$ and $n_{b}$ are Hill coefficients. Setting the left-hand sides of Equations 1, 2, and 3 to zero, the steady state solutions can be obtained as follows:

(Equation 4) $$\begin{array}{c} \bar{\left[\text{THBS}1\right]}=\frac{a}{d_{1}}\left(\frac{{\bar{\left[\text{TGF}\beta 1\right]}}^{n_{a}}}{{\bar{\left[\text{TGF}\beta 1\right]}}^{n_{a}}+K_{a}}\right)\text{,} \end{array}$$

(Equation 5) $$\begin{array}{c} \bar{\left[\text{FMOD}\right]}=\frac{b}{d_{2}}\left(\frac{K_{b}}{{\bar{\left[\text{TGF}\beta 1\right]}}^{n_{b}}+K_{b}}\right)\text{,} \end{array}$$

(Equation 6) $$\begin{array}{c} \bar{\left[\text{TGF}\beta 1\right]}=\frac{c}{d_{3}}\left(\frac{\bar{\left[\text{THBS}1\right]}}{\bar{\left[\text{THBS}1\right]}+K_{1}}\right)\left(\frac{K_{2}}{\bar{\left[\text{FMOD}\right]}+K_{2}}\right)\text{.} \end{array}$$

Substituting (4) and (5) into (6), $\bar{\left[\text{TGF}\beta 1\right]}$ can be computed using Newton’s method and stability can be determined by the sign of the derivative. Note that $\bar{\left[\text{THBS}1\right]}=0$, $\bar{\left[\text{FMOD}\right]}\equiv b/d_{2}$, and $\bar{\left[\text{TGF}\beta 1\right]}\equiv 0$ are stable steady state solutions for $K_{1}>0$ and $K_{2}>0$. By fitting (4) and (5) to the experimental data (Figure 3A), the parameters were inferred using a nonlinear least-squares method with Gnuplot (version 5.4); $a/d_{1}=2.36$, $b/d_{2}=0.33$, $K_{a}=0.016$, $K_{b}=0.002$, $n_{a}=1.6$, and $n_{b}=1.7$ (Figure S8A).

To understand the roles of endogenous TGF-β1, THBS1, and FMOD, we extended (6) to incorporate the effects of PDL and endogenous TGF-β1 production as described below:

(Equation 7) $$\begin{array}{c} \bar{\left[\text{THBS}1\cdot \text{PDL}\right]}=\alpha \left(\frac{\text{PDL}}{\text{PDL}+K_{\alpha }}\right)\text{,} \end{array}$$

(Equation 8) $$\begin{array}{c} \bar{\left[\text{FMOD}\cdot \text{PDL}\right]}=\beta \left(\frac{K_{\beta }}{\text{PDL}+K_{\beta }}\right)\text{,} \end{array}$$

(Equation 9) $$\begin{array}{c} \bar{\left[\text{TGF}\beta 1\right]}=\gamma \left(\frac{\bar{\left[\text{THBS}1\cdot \text{PDL}\right]}}{\bar{\left[\text{THBS}1\cdot \text{PDL}\right]}+\tilde{K_{1}}}\right)\left(\frac{\tilde{K_{2}}}{\bar{\left[\text{FMOD}\cdot \text{PDL}\right]}+\tilde{K_{2}}}\right)\text{,} \end{array}$$

where α, β, and γ are the maximal values of [THBS1·PDL], [FMOD·PDL], and [TGFβ1], and $K_{\alpha }$, $K_{\beta }$, $\tilde{K_{1}}$, and $\tilde{K_{2}}$ are the half saturation constants, respectively. In particular, γ represents the endogenous TGF-β1 production rate. In the same manner as before, we fitted (7)-(9) to the experimental data (Figure S8B) to identify all the parameter values: α=3, β=5, γ=3.5, $K_{\alpha }=10.1$, $K_{\beta }=5$, $\tilde{K_{1}}=0.46$, and $\tilde{K_{2}}=0.62$. The bifurcation diagram is depicted using Equations 4, 5, and 6 by replacing $c/d_{3}$, $K_{1}$, and $K_{2}$ with γ, $\tilde{K_{1}}$, and $\tilde{K_{2}}$ (Figure 4D).

Moreover, for simulating the immunostaining experiment (Figure 4F), we extended the model (4)–(6) to a stochastic model by computing probability distributions of [THBS1] and [FMOD] for a given [TGFβ1]. Using an Ornstein–Uhlenbeck process as a model of gene expression,^82^ we formulated the dynamics of [THBS1] and [FMOD] by the following stochastic differential equations:

(Equation 10) $$\begin{array}{c} d\left[\text{THBS}1\right]=\left(f_{\text{THBS}1}\left(\bar{\left[\text{TGF}\beta 1\right]}\right)-\left[\text{THBS}1\right]\right)dt+\sigma_{1}dB_{1}\left(t\right)\text{,} \end{array}$$

(Equation 11) $$\begin{array}{c} d\left[\text{FMOD}\right]=\left(f_{\text{FMOD}}\left(\bar{\left[\text{TGF}\beta 1\right]}\right)-\left[\text{FMOD}\right]\right)dt+\sigma_{2}dB_{2}\left(t\right)\text{,} \end{array}$$

where $dB_{1}\left(t\right)$ and $dB_{2}\left(t\right)$ are standard Gaussian white noises (mean 0 and variance 1), $\sigma_{1}$ and $\sigma_{2}$ are constant parameters, and $f_{\text{THBS}1}$ and $f_{\text{FMOD}}$ are given by the following functions:

$$f_{\text{THBS}1}\left(\bar{\left[\text{TGF}\beta 1\right]}\right)=\frac{a}{d_{1}}\left(\frac{{\bar{\left[\text{TGF}\beta 1\right]}}^{n_{a}}}{{\bar{\left[\text{TGF}\beta 1\right]}}^{n_{a}}+K_{a}}\right)\text{,}$$

$$f_{\text{FMOD}}\left(\bar{\left[\text{TGF}\beta 1\right]}\right)=\frac{b}{d_{2}}\left(\frac{K_{b}}{{\bar{\left[\text{TGF}\beta 1\right]}}^{n_{b}}+K_{b}}\right)\text{.}$$

In our immunostaining experiment, $\bar{\left[\text{TGF}\beta 1\right]}$ is assumed as a control parameter. Hence, we computed $\bar{\left[\text{TGF}\beta 1\right]}$ as a time limit of [TGFβ1] using the following ODE:

(Equation 12) $$\begin{array}{c} \frac{d\left[\text{TGF}\beta 1\right]}{dt}=f_{\text{TGF}\beta 1}\left(\left[\text{TGF}\beta 1\right]\right)-\left[\text{TGF}\beta 1\right]\text{,} \end{array}$$

where $f_{\text{TGF}\beta 1}\left(\left[\text{TGF}\beta 1\right]\right)$ is given by the following equation:

$$f_{\text{TGF}\beta 1}\left(\left[\text{TGF}\beta 1\right]\right)=\frac{c}{d_{3}}\left(\frac{{\left[\text{TGF}\beta 1\right]}^{n_{a}}}{{\left[\text{TGF}\beta 1\right]}^{n_{a}}+\frac{K_{1}d_{1}}{a}\left({\left[\text{TGF}\beta 1\right]}^{n_{a}}+K_{a}\right)}\right)\left(\frac{\frac{K_{2}d_{2}}{b}\left({\left[\text{TGF}\beta 1\right]}^{n_{b}}+K_{b}\right)}{K_{b}+\frac{K_{2}d_{2}}{b}\left({\left[\text{TGF}\beta 1\right]}^{n_{b}}+K_{b}\right)}\right)\text{.}$$

Note that $\bar{\left[\text{TGF}\beta 1\right]}$ corresponds with Equation 6.

We numerically computed (10) and (11) using Euler-Maruyama method and (12) using Euler method with the following initial conditions:

(Equation 13) $$\begin{array}{c} \left[\text{THBS}1\right]\left(0\right)=0\text{,} \end{array}$$

(Equation 14) $$\begin{array}{c} \left[\text{FMOD}\right]\left(0\right)=0\text{,} \end{array}$$

(Equation 15) $$\begin{array}{c} \left[\text{TGF}\beta 1\right]\left(0\right)∼U\left(0,\;{\left[\text{TGF}\beta 1\right]}_{\max}\right)\text{,} \end{array}$$

where (15) means that the initial value of [TGFβ1] is randomly sampled from a uniform distribution $U\left(0,{\left[\text{TGF}\beta 1\right]}_{\max}\right)$. Note that Equations 10 and 11 may yield negative values. In such a case, we addressed it numerically by constraining them to zero. The resulting bias from this adjustment, however, proved to be negligible. We set the parameter values as follows: $\sigma_{1}=\sigma_{2}=0.2$ and ${\left[\text{TGF}\beta 1\right]}_{\max}=0.2$ in Figure 4C, and $\sigma_{1}=\sigma_{2}=0.05$ and ${\left[\text{TGF}\beta 1\right]}_{\max}=1.0$ in Figure S10B. We simulated independent 10,000 sample paths for long time enough and obtained a steady state distribution of [THBS1] and [FMOD].

#### TGF-βR inhibitor treatment for endogenous TGF-β signaling

HFF-1 cells (PDL 24, PDL 36, PDL 47, and PDL 53) were seeded in 6-well plates with DMEM at 200,000 cells/well. Cells were treated with control (DMEM with 2% FBS-added vehicle) or 5 μM LY364947 (123-05981, Fujifilm), and the lysates were collected after 48 h for quantification by WB.

#### Time-course datasets for mathematical model training

We used the time-course data for phosphorylated SMAD3, c-Fos, THBS1, phosphorylated Akt (Ser473), and FMOD activity with or without 4 ng/mL TGF-β1 (eight time-points up to 48 h) treatment in HFF-1 cells for data-fitting of the comprehensive model. Briefly, HFF-1 cells were seeded in 6-well plates at 200,000 cells/well and maintained in DMEM supplemented with 10% FBS. After serum starvation for 16 h, the cells were treated with either control (DMEM with vehicle supplemented with 2% FBS) or 4 ng/mL TGF-β1 (R&D Systems) and the lysate and supernatant were collected at 0 min, 15 min, 30 min, 60 min, 120 min, 8 h, 24 h, and 48 h. Reconstitution buffer (0.1% BSA in 4 mM HCl PBS, R&D Systems) was used as vehicle. The cells were lysed with RIPA buffer (Thermo Fisher Scientific) supplemented with Halt™ Protease and Phosphatase Inhibitor (Thermo Fisher Scientific) and used in the western blot analysis for analyzing anti-phosphorylated SMAD3 (Ser423/425; 9520, CST), anti-c-Fos (2250, CST), anti-THBS1 (37879, CST), and anti-phosphorylated Akt (Ser473; 9271, CST) expression. Supernatants were centrifuged (13,000 rpm, 15 min, 4°C) to remove cell debris and used for the FMOD ELISA (ab275895, Abcam). In addition, anti-phosphorylated SMAD2 data (Ser465/467; 3108, CST) were obtained for model validation and not used in data-fitting. The data were normalized between the minimum (0) and maximum (1) values.

#### Model simulation and parameter estimation

We constructed the comprehensive mathematical model linking the TGF-β and VEGF signaling pathways (Figure S11) by integrating two mathematical models using a Python framework for Modeling and Analysis of Signaling Systems (BioMASS).^24^^,^^53^

Parameter estimation was conducted in two steps. In step one, parameters related to the TGF-β signaling network, modified based on the Lucarelli model,^76^ were trained using normalized phosphorylated SMAD3, c-Fos, and THBS1 time-course expression. The resulting model had 27 rate equations, 22 species, and 42 parameters, of which 34 were to be estimated. By minimizing the objective function, i.e., the sum of residual squares between simulation and experimental values, 30 fitting parameter sets were obtained that reproduce the experimental results with/without TGF-β1 stimulation in HFF-1 cells. In step two, an additional 30 parameter sets for the rest of the model, including the Raf-ERK cascade and PI3K-Akt pathway, were trained using normalized phosphorylated SMAD3, c-Fos, THBS1, phosphorylated Akt, and FMOD time-course expression (Figure 5A). The best fitting parameters in the TGF-β signaling network were adapted from step one. The resulting integrated model had 79 rate equations, 83 species, and 194 parameters. An additional 30 fitting parameter sets were obtained that reproduce the experimental results of TGF-β1 stimulation in HFF-1 cells (parameter range: Figure S12B; objective function trace: Figures S12C and S12D).

For the TGF-β pathway, we developed an original model of TGF-βR activation, SMAD phosphorylation, and SMAD complex formation. This model activates the transcription of c-Fos at the same time and forms a logical AND gate with SMAD complexes to regulate THBS1 expression. THBS1 forms positive feedback and activates latent TGF-β1. We modified the Imoto model^53^ to describe the process from VEGFR receptor activation to ERK phosphorylation. ERK phosphorylation results in the activation of TFs for FMOD transcription. FMOD then forms a complex with activated TGF-β1 and inhibits its binding to TGF-βR. The TGF-β and VEGF signaling pathway models crosstalk via the PI3K-Akt pathway.

All related files to execute the TGF-β and TGF-β VEGF model using BioMASS can be found at https://github.com/okadalabipr/Haga2023. We described each biochemical reaction using ODEs. To train model parameters, we used time-series expression data for HFF-1 cells. For the global parameter estimation, we minimized the sum of squared differences between the experimental observations and simulated values using Differential Evolution.^83^

#### Initial value for mathematical model

Values measured by ELISA and RNA-seq were used to determine the initial values of the model.

The initial value for SMAD2, SMAD3, and SMAD4 were quantified using the respective ELISA kits (SMAD2: 0.060 nM, SMAD3: 0.38 nM, SMAD4: 0.0044 nM). Briefly, HFF-1 cells were seeded in 6-well plates with DMEM at 200,000 cells/well. Cell lysates were collected using the lysate buffer provided with the ELISA kits after serum starvation for 16 h, centrifuged (13,000 rpm, 15 min, 4°C), and used for SMAD2 (ab260065, Abcam), SMAD3 (ab264624, Abcam), and SMAD4 ELISAs (ab253211, Abcam).

For other nonzero species, we used RNA-seq data obtained from non-stimulated HFF-1 cells to determine the initial value. Briefly, HFF-1 cells were seeded in 6-well plates with DMEM at 200,000 cells/well. Total RNA (three wells per sample) was collected after serum starvation for 16 h. RNA purification, sequencing library preparation, and downstream analysis was conducted as described in the “Sample preparation for RNA-seq and genomic alignment” section. Initial protein levels of the model species (total of 37 genes) were inferred from RNA-seq data, where the maximal transcription rate or translated protein level were estimated from the mRNA level.^24^^,^^53^ We estimated protein amounts from one or more genes belonging to the same gene family (isoforms) and employed weighting factors to convert the TPM value of the gene corresponding to the protein to the appropriate initial protein value. The TPM values are shown in Table S4.

#### Sensitivity analysis

The sensitivity coefficient Sy was calculated using the following equation:^24^^,^^53^

$$\text{Sy}=\partial \text{lnM}/\partial \ln\;y_{j}$$

where M is the signaling metric, i.e., the integral expression level of THBS1 with TGF-β1 stimulation, and *y*_*j*_ is each nonzero species in the mechanistic model. The sensitivity coefficients were calculated by finite difference approximations with 1% changes in the biochemical reactions. To calculate the sensitivity coefficients, we used BioMASS with run_analysis = (target=“reaction”, metric=‘‘integral’’, style=‘‘heatmap’’).

#### Time-course PI3K-Akt inhibitor treatment

We validated the model results using HFF-1 cell experiments with additional inhibitors at each time-point (four time-points, up to 48 h). Briefly, HFF-1 cells were seeded in 96-well plates with DMEM at 10,000 cells/well. The cells were treated with either control (DMEM with 0.1% DMSO supplemented with 2% FBS) or 4 ng/mL TGF-β1 (R&D Systems). Inhibitors for TGF-β1-treated samples were added at 0 h, 8 h, 24 h, and 32 h. Supernatants were collected at 48 h for quantification. Reconstitution buffer (0.1% BSA in 4 mM HCl PBS, R&D Systems) was used as vehicle. Supernatants were centrifuged (13,000 rpm, 15 min, 4°C) to remove cell debris and used for FMOD ELISA (Abcam). The following inhibitors were used: LY364947 (123-05981, Fujifilm) and Akt inhibitor VIII (14870, Cayman Chemical).

#### Sample preparation for THBS1 and FMOD quantification with TGF-β family

HFF-1 cells were used to prepare recombinant human TGF-β family treated lysates and supernatants for quantification. Briefly, cells for were seeded in 6-well plates at 200,000 cells/well. After treatment with either control (DMEM with vehicle supplemented with 2% FBS), TGF-β1 (R&D Systems), TGF-β2 (ProteinTech), or TGF-β3 (ProteinTech), cell lysates and supernatants were collected after 48 h for quantification by WB or FMOD ELISA (Abcam).

#### Sample preparation for FMOD quantification with VEGF, EGF, b-FGF, or PDGF-BB

HFF-1 cells were used to prepare recombinant human VEGF, EGF, b-FGF, or PDGF-BB supernatants for FMOD ELISA. Briefly, cells for were seeded in 12-well plates at 100,000 cells/well. After treatment with either control (DMEM with vehicle supplemented with 2% FBS), VEGF (ProteinTech), EGF (PeproTech), b-FGF (R&D Systems), or PDGF-BB (ProteinTech), cell supernatants were collected after 48 h for quantification by FMOD ELISA (Abcam).

#### Time-course TGF-β1 washout experiment

TGF-β1-stimulated HFF-1 cells was washed with PBS at seven time-points (i.e., 1 h, 2 h, 4 h, 8 h, 12 h, 24 h, and 48 h or 15 min, 30 min, 1 h, 4 h, 12 h, 24 h, and 48 h) to confirm the effects of transient and sustained TGF-β1 stimulation on the expression of THBS1, FMOD, and phosphorylated Akt (Ser473). Briefly, HFF-1 cells were seeded in 6-well plates with DMEM at 200,000 cells/well. After serum starvation for 16 h, cells were treated with control (DMEM with 2% FBS-added vehicle) or 4 ng/mL TGF-β1. Cells were washed with PBS and replaced with control medium at each time-point from the TGF-β1 stimulation condition (Figure S9A), and all lysate was recovered at 48 h for western blot analysis.

#### THBS1 immunofluorescence imaging

The distribution of THBS1 expression in HFF-1 was examined by immunofluorescence imaging. Briefly, HFF-1 cells were seeded in 24-well plates with DMEM at 20,000 cells/well. After serum starvation for 16 h, cells were treated with control (DMEM with 2% FBS-added vehicle), 0.04 ng/mL, or 0.2 ng/mL TGF-β1 for 48 h and fixed with fresh 4% paraformaldehyde (Thermo Fisher Scientific) in PBS for 15 min (N=6). After rinsing with PBS, the cells were subjected to a 0.2% TritonX-100 (Nakalai Tesque) in PBS for 10 min and blocked using 1% goat serum (Thermo Fisher Scientific) in PBS overnight. Next, cells were incubated with THBS1 antibodies (ab85762, Abcam) diluted in 1% goat serum in PBS overnight and washed with PBS. Finally, cells were incubated with Alexa Fluor® 594 conjugated secondary antibodies (Abcam), CellMask™ (Thermo Fisher Scientific) for cell body staining, and Hoechst® 33342 (DOJINDO) for nuclear staining for 1 h at 25°C in the dark. Fluorescence images of 96 fields (16 fields per well) under each condition were acquired using IN Cell Analyzer 2500HS (Cytiva). CellProfiler (ver. 4.2.1) was used to segment nuclear regions from Hoechst® 33342 images and cytoplasmic regions by excluding nuclear regions from CellMask™ images. The THBS1 signal intensity of each cell was then quantified in the cytoplasmic region; THBS1 signal intensity was calculated based on the integrated and mean signal density in each image.

#### Western blot analysis

All immunoblots are representative images of three biological replicates. Attached cells were washed with PBS and lysed in RIPA buffer (Thermo Fisher Scientific) supplemented with Halt™ Protease and Phosphatase Inhibitor (Thermo Fisher Scientific). Cell lysates were centrifuged (13,000 rpm, 15 min, 4°C) to unify protein concentrations between samples, and a BCA protein assay kit (Thermo Fisher Scientific) was used according to the manufacturer’s protocol. Proteins were separated by SDS-PAGE and transferred to nitrocellulose membranes using iBlot2 (Thermo Fisher Scientific). After blocking with EveryBlot blocking buffer (Bio-Rad), the following antibodies were used for blotting: anti-THBS1 (37879, CST), anti-FMOD (60108-1-Ig, ProteinTech), anti-LAMIN-B1 (12987-1-AP, ProteinTech), anti-p53 (2524, CST), anti-p21 (2946, CST), anti-c-Fos (2250, CST), anti-c-Jun (9165, CST), anti-SMAD2 (5339, CST), anti-phosphorylated SMAD2 (Ser465/467; 3108, CST), anti-SMAD3 (9523, CST), anti-phosphorylated SMAD3 (Ser423/425; 9520, CST), anti-SMAD4 (46535, CST), anti-phosphorylated Akt (Ser473; 9271, CST), anti-pan Akt (2920, CST), anti-USP11 (ab109232, Abcam), anti-GAPDH (M171-3, Medical & Biological Laboratories), and anti-GAPDH (10494-1-Ap, ProteinTech). Primary antibodies were reacted at 4°C overnight, while secondary antibodies were reacted at room temperature for 1 h. For protein detection, Clarity Western ECL Substrate (Bio-Rad) or Clarity Max Western ECL Substrate (Bio-Rad) was used with an Amersham Imager 680 (GE Healthcare). Relative protein quantification was performed with ImageJ Fiji. Expression values (N=3) were normalized using GAPDH as loading control and the ratio against the control was calculated. Molecular weights are indicated on the left of each image.

#### Enzyme-linked immunosorbent assay (ELISA)

All ELISAs were conducted according to the manufacturer’s protocol. Cell supernatants were used as samples for ELISAs for THBS1, FMOD, TGF-β1, VEGF, EGF, b-FGF, PDGF, IL-6, and IL-8. Briefly, supernatant samples were centrifuged (1,000 rpm, 4 min, 4°C) to remove cell debris and used for each measurement. Cell lysates were used as samples for the ELISA for SMAD2, SMAD3, and SMAD4. Lysate samples were collected using lysis buffer included in the kits, cell lysates were centrifuged (13,000 rpm, 15 min, 4°C) and used for each ELISA. Relative expression levels are displayed with the control (1) value. The following ELISA kits were used for quantification: THBS1 (DTSP10-1, R&D Systems), FMOD (ab275895, Abcam), TGF-β1 (DB100B, R&D Systems), VEGF (DVE00, R&D Systems), IL-6 (D6050, R&D Systems), IL-8 (ab214030, Abcam), SMAD2 (ab260065, Abcam), SMAD3 (ab264624, Abcam), and SMAD4 (ab253211, Abcam).

#### Quantitative RT-PCR (qRT-PCR) analysis

Total RNA from HFF-1 cells was prepared using the NucleoSpin RNA kit (Macherey-Nagel GmbH & Co.) and subjected to complementary DNA (cDNA) synthesis using ReverTra Ace® qPCR RT Master Mix (Toyobo Life Science) as follows: 15 min at 37°C, 5 min at 50°C, and 5 min at 98°C. Quantitative PCR using cDNA was conducted using a KOD SYBR qPCR kit (Toyobo Life Science) with a CFX96 Real-Time PCR System (Bio-Rad) according to the manufacturer’s protocol. PCR cycling conditions were as follows: 40 cycles of 10 s at 98°C, 10 s at 60°C, and 30 s at 68°C. The primers used for qRT-PCR were as follows: THBS1 (5′-TCCCCATCCAAAGCGTCTTC-3′ and 5′-ACCACGTTGTTGTCAAGGGT-3′); FMOD (5′-GGACGTGGTCACTCTCTGAA-3′ and 5′-GGCTCGTAGGTCTCATACGG-3′); GAPDH (5′-GTCTCCTCTGACTTCAACAGCG-3′ and 5′-ACCACCCTGTTGCTGTAGCCAA-3′). Gene expression was quantified using the ΔΔCq method. Expression values (N=3) were normalized using GAPDH and the ratio against the PDL 24 value was calculated.

### Quantification and statistical analysis

Statistical data are presented as the mean with standard deviation (SD) calculated using the “sd” function of R. The horizontal line in the center of the box plot is the median, and the lower and upper borders indicate the 25th and 75th percentiles, respectively, and all measurements are shown as point plots. For detection of upstream regulators, the right-tailed Fisher’s exact test was used in the IPA. Comparisons of more than two groups were made using a one-way Dunnett’s or Tukey’s multiple comparisons test. Comparisons of two groups were evaluated by Student’s *t*-test, Welch’s *t*-test, or Wilcoxon rank sum test. The Silverman test was used to test for multimodality. We considered p<0.05 to be statistically significant. Unless otherwise noted, p-values were calculated using the multcomp package in R. The statical details of each experiment can be found in the figure legends.
