# Supplementary material for: Positive and negative feedback regulation of the TGF-β1 explains two equilibrium states in skin aging
Source: iScience. 2024 Apr 10;27(5):109708. doi: 10.1016/j.isci.2024.109708 (PMC11066433; doi:10.1016/j.isci.2024.109708)
Supplement: Document S1. Figures S1–S13 and Tables S1–S4 [file mmc1.pdf]

iScience, Volume *27*

Supplemental information

Positive and negative feedback regulation of the TGF-β1 explains two equilibrium states in skin aging

Masatoshi Haga, Keita Iida, and Mariko Okada

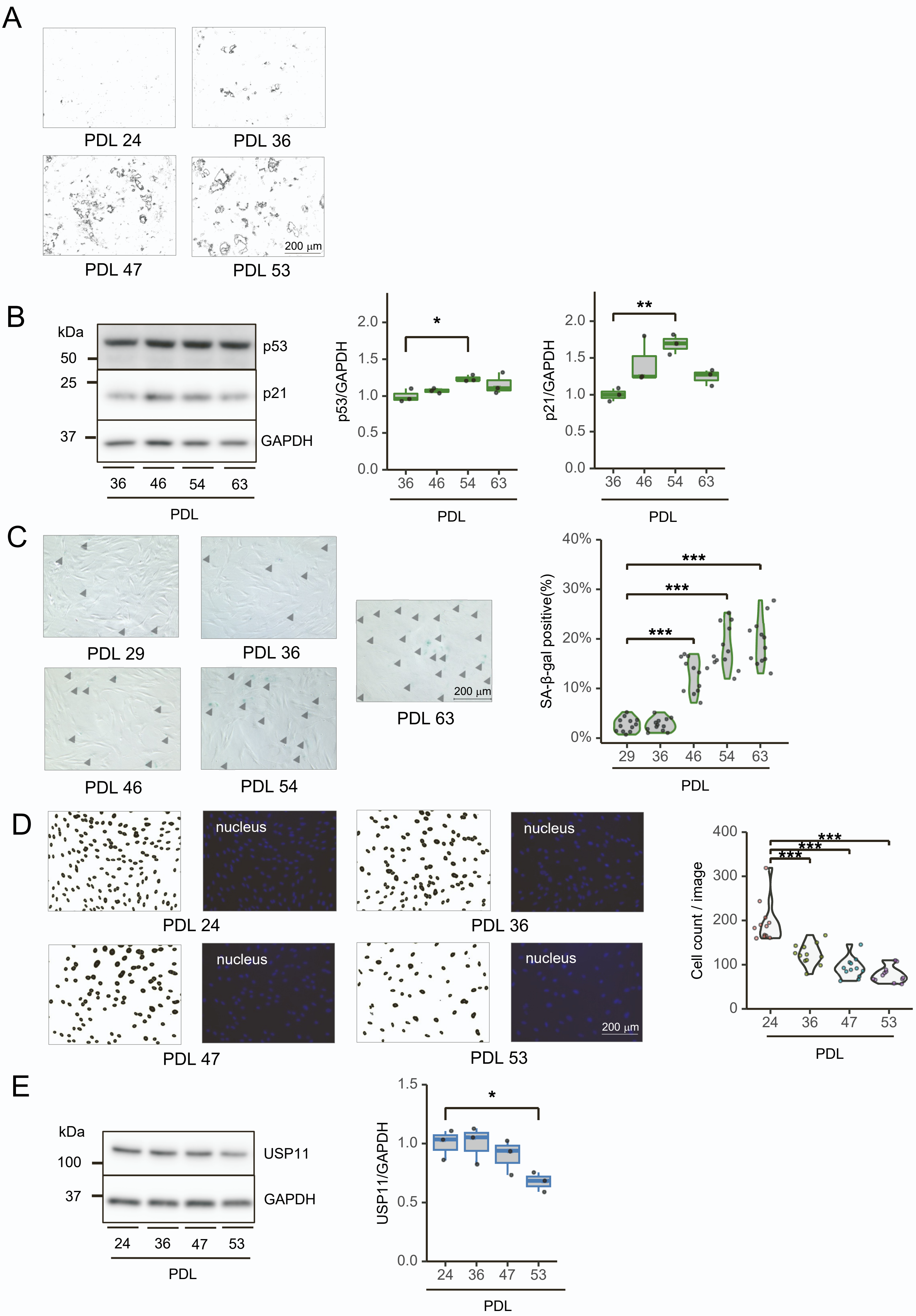
A
PDL 24
PDL 36
PDL 47
PDL 53
200 µm
B
kDa
50
25
37
p53
p21
GAPDH
36
46
54
63
PDL
p53/GAPDH
p21/GAPDH
2.0
1.5
1.0
0.5
0.0
*
**
C
PDL 29
PDL 36
PDL 46
PDL 54
PDL 63
200 µm
SA-β-gal positive(%)
40%
30%
20%
10%
0%
***
29
36
46
54
63
PDL
D
nucleus
PDL 24
PDL 36
PDL 47
PDL 53
200 µm
Cell count / image
400
300
200
100
0
***
24
36
47
53
PDL
E
kDa
100
37
USP11
GAPDH
24
36
47
53
PDL
USP11/GAPDH
1.5
1.0
0.5
0.0
*

Figure S1: Analysis of replication-stress-induced dermal fibroblasts, related to Figure 1.

A) SA-β-gal staining of replication-stress-induced HFF-1 cells. SA-β-gal-positive cells for each PDL (PDL 24, PDL 36, PDL 47, PDL 53) are shown. Scale bar: 200 μm. Images were processed using Image J software (see details in STAR Methods section); raw images are shown in Figure 1D.

B) Western blot (WB) of p53 and p21 in replication-stress-induced BJ cells. Cells of each PDL (PDL 36, PDL 46, PDL 54, PDL 63) were cultured for 48 h and the lysates were analyzed. (Left panel) Representative image. (Middle panel) Quantification of p53 expression; N=3, *$p<0.05$. (Dunnett's test). (Right panel) Quantification of p21 expression; N=3, **$p<0.01$ (Dunnett's test).

C) (Left panel) SA-β-gal staining of replication-stress-induced BJ cells. Representative image. SA-β-gal-positive cells for each PDL (PDL 29, PDL 36, PDL 46, PDL 54, PDL 63) are indicated with black arrowheads; scale bars: 200 μm. (Right panel) Quantification of SA-β-gal: SA-β-gal-positive rate (%) =number of SA-β-gal-positive cells / total number of cells × 100. Four images from each of the three wells were analyzed (total 12 images/condition), ***$p<0.001$ (Dunnett's test).

D) Cell counting with Hoechst® 33342 in replication-stress-induced HFF-1 cells. Cells from each PDL (PDL 24, PDL 36, PDL 47, PDL 53) were cultured for 48 h and the number of nuclei were counted. (Left panel) Representative image. Each image was processed using Image J. (Right panel) Quantification of cell count per image of each PDL. Four images from each of the three wells were analyzed (total of 12 images/PDL), ***$p<0.001$ (Dunnett's test).

E) WB of USP11 in replication-stress-induced HFF-1 cells. Cells of each PDL (PDL 24, PDL 36, PDL 47, PDL 53) were cultured for 48 h, and lysates were analyzed. (Left panel) Representative image. (Right panel) Quantification of USP11 expression; N=3, *$p<0.05$ (Dunnett's test).

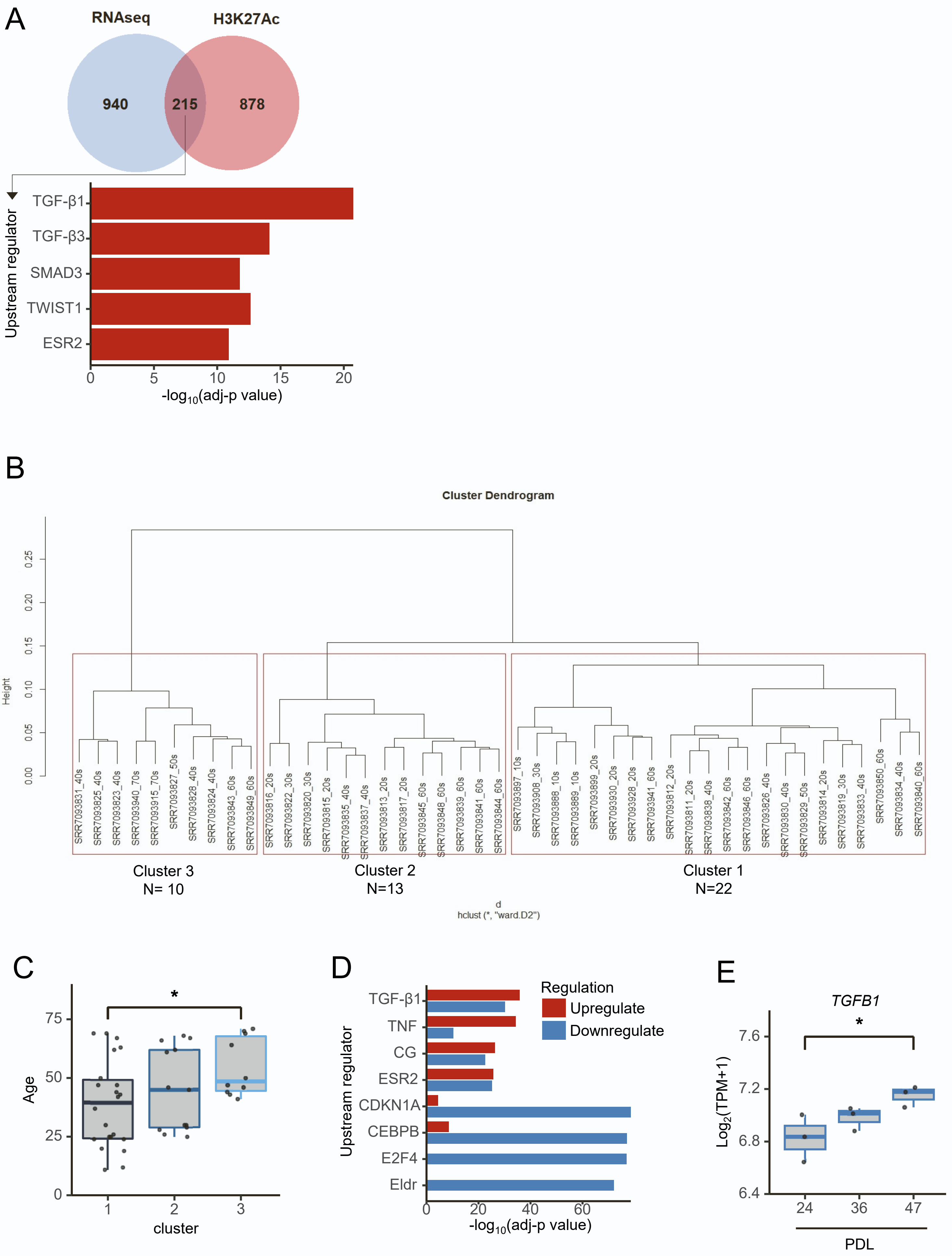
A
RNAseq
H3K27Ac
940
215
878
Upstream regulator
TGF-β1
TGF-β3
SMAD3
TWIST1
ESR2
0
5
10
15
20
-log10(adj-p value)
B
Cluster Dendrogram
Height
0.00
0.05
0.10
0.15
0.20
0.25
SRR7093831_40s
SRR7093825_40s
SRR7093823_40s
SRR7093940_70s
SRR7093915_70s
SRR7093827_50s
SRR7093828_40s
SRR7093824_40s
SRR7093843_60s
SRR7093849_60s
SRR7093816_20s
SRR7093822_30s
SRR7093820_30s
SRR7093815_20s
SRR7093835_40s
SRR7093837_40s
SRR7093813_20s
SRR7093817_20s
SRR7093845_60s
SRR7093848_60s
SRR7093839_60s
SRR7093841_60s
SRR7093844_60s
SRR7093897_10s
SRR7093908_30s
SRR7093888_10s
SRR7093889_10s
SRR7093899_20s
SRR7093930_20s
SRR7093928_20s
SRR7093941_60s
SRR7093812_20s
SRR7093811_20s
SRR7093838_40s
SRR7093842_60s
SRR7093846_60s
SRR7093826_40s
SRR7093830_40s
SRR7093829_50s
SRR7093814_20s
SRR7093819_30s
SRR7093833_40s
SRR7093850_60s
SRR7093834_40s
SRR7093840_60s
Cluster 3
N= 10
Cluster 2
N=13
Cluster 1
N=22
d
hclust (*, "ward.D2")
C
*
Age
75
50
25
0
1
2
3
cluster
D
Upstream regulator
TGF-β1
TNF
CG
ESR2
CDKN1A
CEBPB
E2F4
Eldr
Regulation
Upregulate
Downregulate
0
20
40
60
-log10(adj-p value)
E
TGFB1
*
Log2(TPM+1)
7.6
7.2
6.8
6.4
24
36
47
PDL

Figure S2: Analysis of in-house and public omics data on senescence and aging, related to Figure 1.

A) (Upper panel) Venn diagram showing RNA-seq upregulated DEGs (blue sphere; FC>1.2, adj-p<0.05) and genes annotated from H3K27Ac differentially gained peaks (red sphere; log_2FC>0, adj-p<0.05); numbers of genes in each sphere are shown. (Bottom panel) The top five upstream regulators determined using Ingenuity Pathway Analysis. A right-tailed Fisher's exact test was used to calculate a p-value of overlap.

B) Clustering of public data on human skin fibroblast RNA-seq samples. Samples from human arm skin fibroblast (age: 11–71 years, N=45) were clustered into cluster 1 (N=22), cluster 2 (N=13), and cluster 3 (N=10) using the "hclust" function of R with "method = ward.D2".

C) Age distribution of the clustered human skin fibroblast samples. Median (cluster 1: 39.5 yrs; cluster 2: 45.0 yrs; cluster 3: 48.5 yrs) and average (cluster 1: 38.9 yrs; cluster 2: 44.8 yrs; cluster 3: 54.5 yrs) of each cluster increased with the cluster number; N=3, *p<0.05 (Wilcoxon rank sum test).

D) Upstream regulators in common genes between the public *in vitro* and *in vivo* datasets. The top four upstream regulators of 592 downregulated (blue) and 502 upregulated (red) genes are shown with colored bars. A right-tailed Fisher's exact test was used to calculate the adj-p value of overlap.

E) Quantification of RNA-seq expression data from replication-stress-induced HFF-1 cells (blue) for *TGFB1*. Gene expression was normalized to log_2(transcripts per million [TPM] + 1); N=3, ***p<0.001 (Dunnett's test).

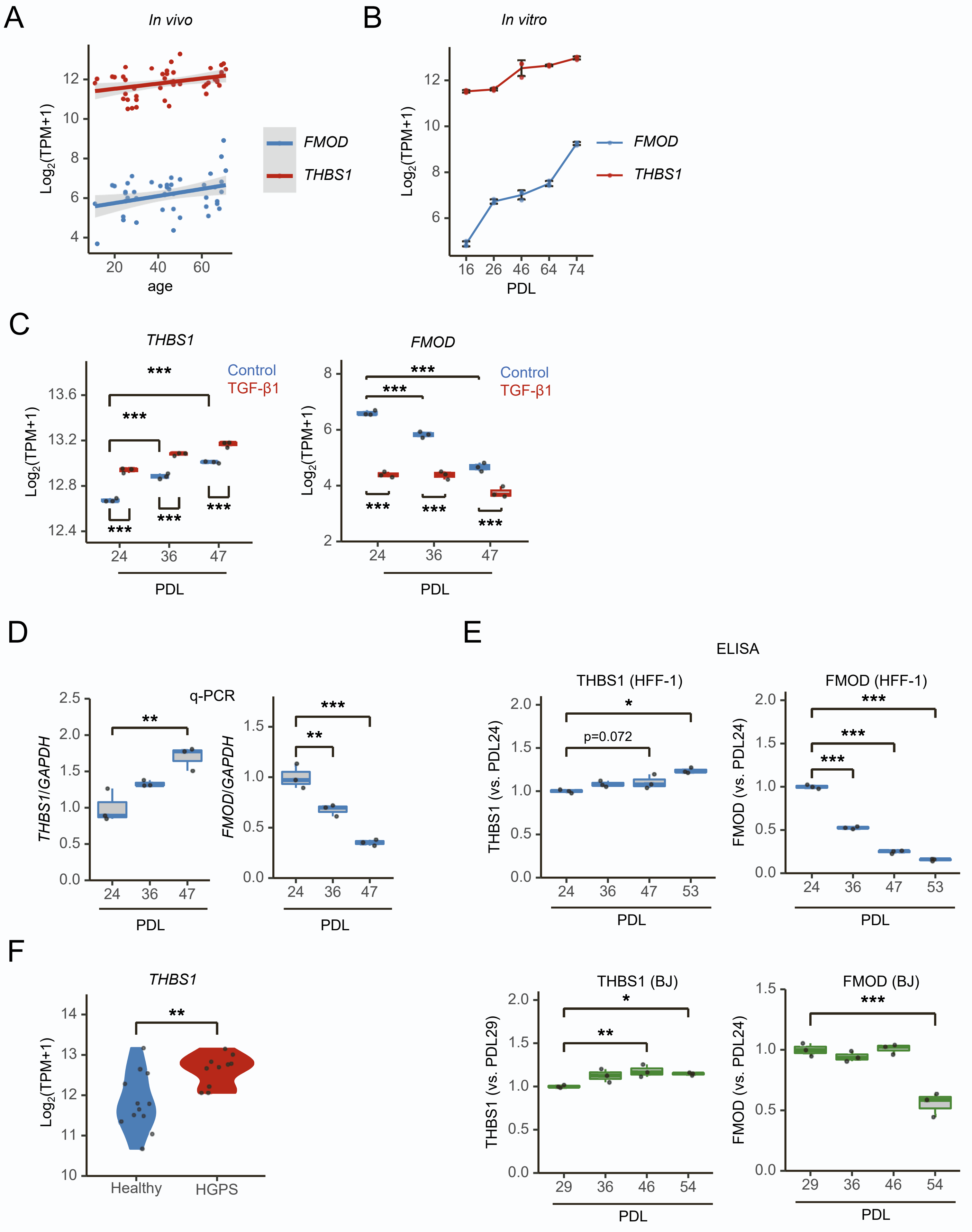
A
In vivo
Log2(TPM+1)
age
FMOD
THBS1
B
In vitro
Log2(TPM+1)
PDL
FMOD
THBS1
C
THBS1
Control
TGF-β1
Log2(TPM+1)
PDL
FMOD
Control
TGF-β1
Log2(TPM+1)
PDL
D
q-PCR
THBS1/GAPDH
PDL
FMOD/GAPDH
PDL
E
ELISA
THBS1 (HFF-1)
p=0.072
THBS1 (vs. PDL24)
PDL
FMOD (HFF-1)
FMOD (vs. PDL24)
PDL
THBS1 (BJ)
THBS1 (vs. PDL29)
PDL
FMOD (BJ)
FMOD (vs. PDL24)
PDL
F
THBS1
Log2(TPM+1)
Healthy
HGPS

Figure S3: Expression changes in THBS1 and FMOD during dermal senescence and aging, related to Figure 1 and 2.

A) Public *in vivo* time-course expression data[27] for *THBS1* and *FMOD*. Gene expression was normalized to $\log_2$(TPM + 1); N=45 (age: 11–71 years; see donor list in Table S1). Linear regression lines were added using 'geom_smooth' (method = "lm").

B) Public *in vitro* time-course expression data[26] for *THBS1* and *FMOD*. Gene expression was normalized to $\log_2$(TPM + 1); N=3, mean ± SD.

C) RNA-seq expression data for *THBS1* and *FMOD* in replication-stress-induced and TGF-β1-stimulated HFF-1 cells (control: blue; 4 ng/mL TGF-β1: red). Gene expression was normalized to $\log_2$(TPM + 1). (Left panel) Quantification of THBS1; N=3, ***p<0.001 (Tukey's multiple comparisons). (Right panel) Quantification of FMOD; N=3, ***p<0.001 (Tukey's multiple comparisons).

D) q-PCR analysis of replication-stress-induced HFF-1 cells. (Left panel) Quantification of *THBS1*; N=3, **p<0.01 (Dunnett's test). (Right panel) Quantification of *FMOD*; N=3, **p<0.01, ***p<0.001 (Dunnett's test).

E) THBS1 and FMOD ELISA of replication-stress-induced HFF-1 (blue) and BJ (green) cell supernatants. Cells were cultured for 48 h and supernatants were analyzed. (Upper left) Quantification of THBS1 in HFF-1 cells; N=3, *p<0.05 (Dunnett's test). (Upper right) Quantification of FMOD in HFF-1 cells; N=3, ***p<0.001 (Dunnett's test). (Bottom left) Quantification of THBS1 in BJ cells; N=3, *p<0.05, **p<0.01 (Dunnett's test). (Bottom right) Quantification of FMOD in BJ cells; N=3, ***p<0.001 (Dunnett's test).

F) *THBS1* expression between healthy participants and patients with Hutchinson–Gilford progeria syndrome (HGPS). Public RNA-seq data[27] for healthy participants (age: 1–9 years, N=12) and patients with HGPS (age: 2–8 years, N=10) derived from dermal fibroblasts. Gene expression was normalized to $\log_2$(TPM + 1). **p<0.01 (Welch's *t*-test).

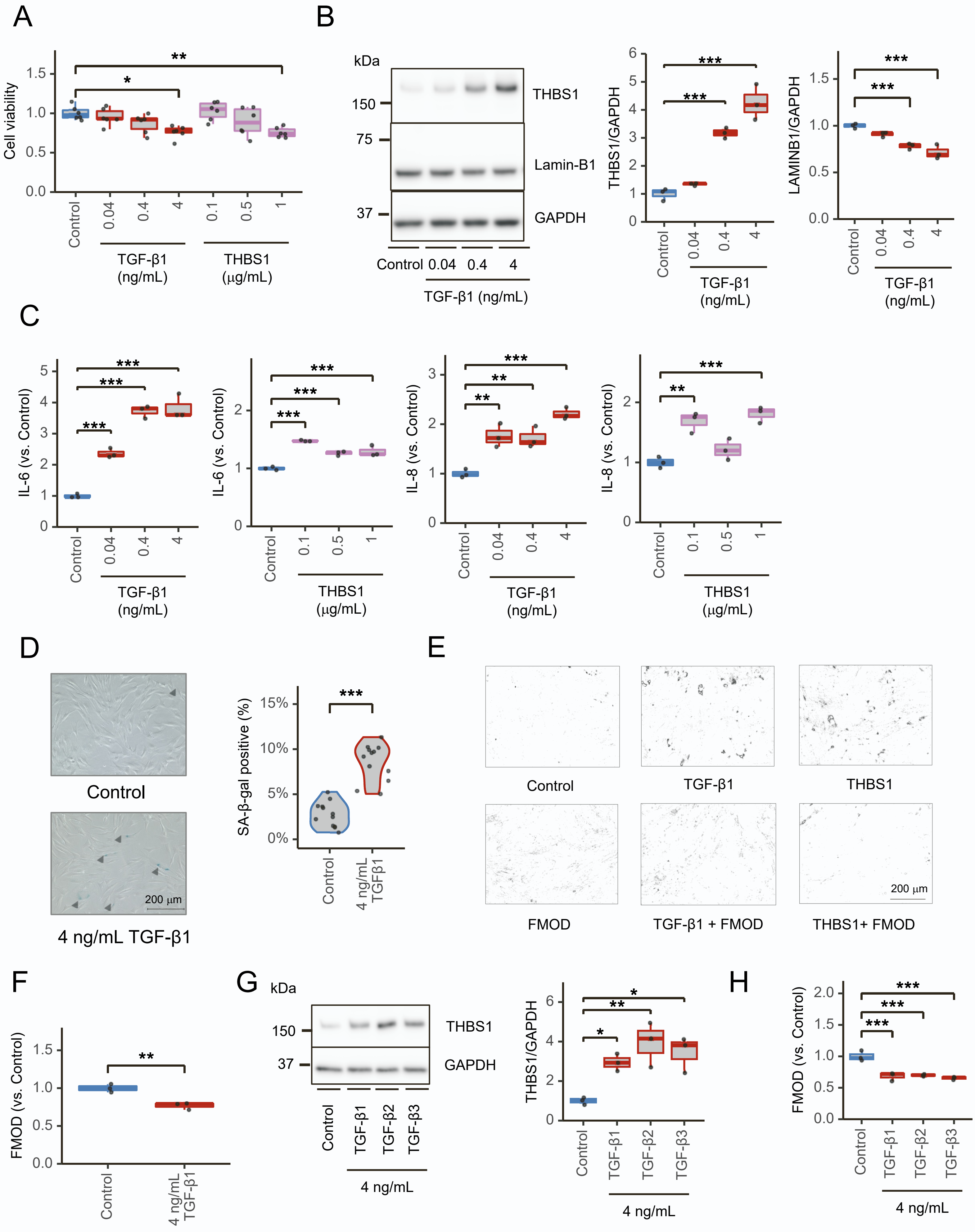
A
B
C
D
E
F
G
H
Cell viability
TGF-β1 (ng/mL)
THBS1 (µg/mL)
kDa
THBS1
Lamin-B1
GAPDH
Control 0.04 0.4 4
TGF-β1 (ng/mL)
THBS1/GAPDH
LAMINB1/GAPDH
IL-6 (vs. Control)
IL-8 (vs. Control)
Control
4 ng/mL TGF-β1
200 µm
SA-β-gal positive (%)
TGF-β1
THBS1
FMOD
TGF-β1 + FMOD
THBS1+ FMOD
FMOD (vs. Control)
TGF-β2
TGF-β3
4 ng/mL

Figure S4: Senescence induced by TGF-β family and THBS1 treatment, related to Figure 2 and 3.

A) Cell viability analysis of TGF-β1- and THBS1-stimulated HFF-1 cells. After 24 h of treatment (control: blue, TGF-β1: red, THBS1: purple), cell viability was measured using the tetrazolium salt WST-8; N=6, *p<0.05, **p<0.01 (Tukey's multiple comparisons).

B) WB of THBS1 and Lamin-B1 in TGF-β1-stimulated BJ cells. Cell lysates were collected 48 h after control (blue) or TGF-β1 (red) treatment. (Left panel) Representative image. (Middle panel) Quantification of THBS1; N=3, ***p<0.001 (Dunnett's test). (Right panel) Quantification of Lamin-B1; N=3, ***p<0.001 (Dunnett's test).

C) IL-6 and IL-8 ELISA in TGF-β1- or THBS1-stimulated BJ cells. Cell supernatants were collected at 48 h after control (blue), TGF-β1 (red), or THBS1 (purple) treatment. (First panel) Quantification of IL-6 ELISA with TGF-β1 treatment; N=3, ***p<0.001 (Dunnett's test). (Second panel) Quantification of IL-6 ELISA with THBS1 treatment; N=3, ***p<0.01 (Dunnett's test). (Third panel) Quantification of IL-8 ELISA with TGF-β1 treatment; N=3, **p<0.01, ***p<0.001 (Dunnett's test). (Fourth panel) Quantification of IL-8 ELISA with THBS1 treatment; N=3, **p<0.01, ***p<0.001 (Dunnett's test).

D) Effect of TGF-β1 simulation on SA-β-gal activity in BJ cells. (Left panel) Representative images. SA-β-gal-positive cells are shown with black arrowheads; scale bar: 200 μm. (Right panel) Quantification of SA-β-gal: SA-β-gal-positive rate (%) =number of SA-β-gal-positive cells / total number of cells × 100. Four images from each of the three wells were analyzed (total 12 images/condition), ***p<0.001 (vs. control, Student's t-test).

E) SA-β-gal staining of TGF-β1 or THBS1 treatment and inhibition by FMOD using HFF-1 cells. Scale bar: 200 μm. Each image was processed using Image J software (see details in STAR Methods section). Raw images are shown in Figure 2I.

F) FMOD ELISA in TGF-β1-stimulated BJ cells. Cells were treated with control (blue) or 4 ng/mL TGF-β1 (red) for 48 h and their supernatants were analyzed; N=3, **p<0.01 (Student's *t*-test).

G) WB analysis of TGF-β family-stimulated HFF-1 cells. Cell lysates were collected 48 h after control (blue) or 4ng/mL TGF-β family (red; with TGF-β1, TGF-β2, TGF-β3) treatment and the lysates were analyzed. (Left panel) Representative image. (Right panel) Quantification of THBS1 expression; N=3, *p<0.05, **p<0.01 (Dunnett's test).

H) FMOD ELISA in TGF-β family-stimulated HFF-1 cells. Cells were treated with control (blue) or 4 ng/mL TGF-β family (red; with TGF-β1, TGF-β2, TGF-β3) for 48 h and their supernatants were analyzed; N=3, ***p<0.001 (Dunnett's test).

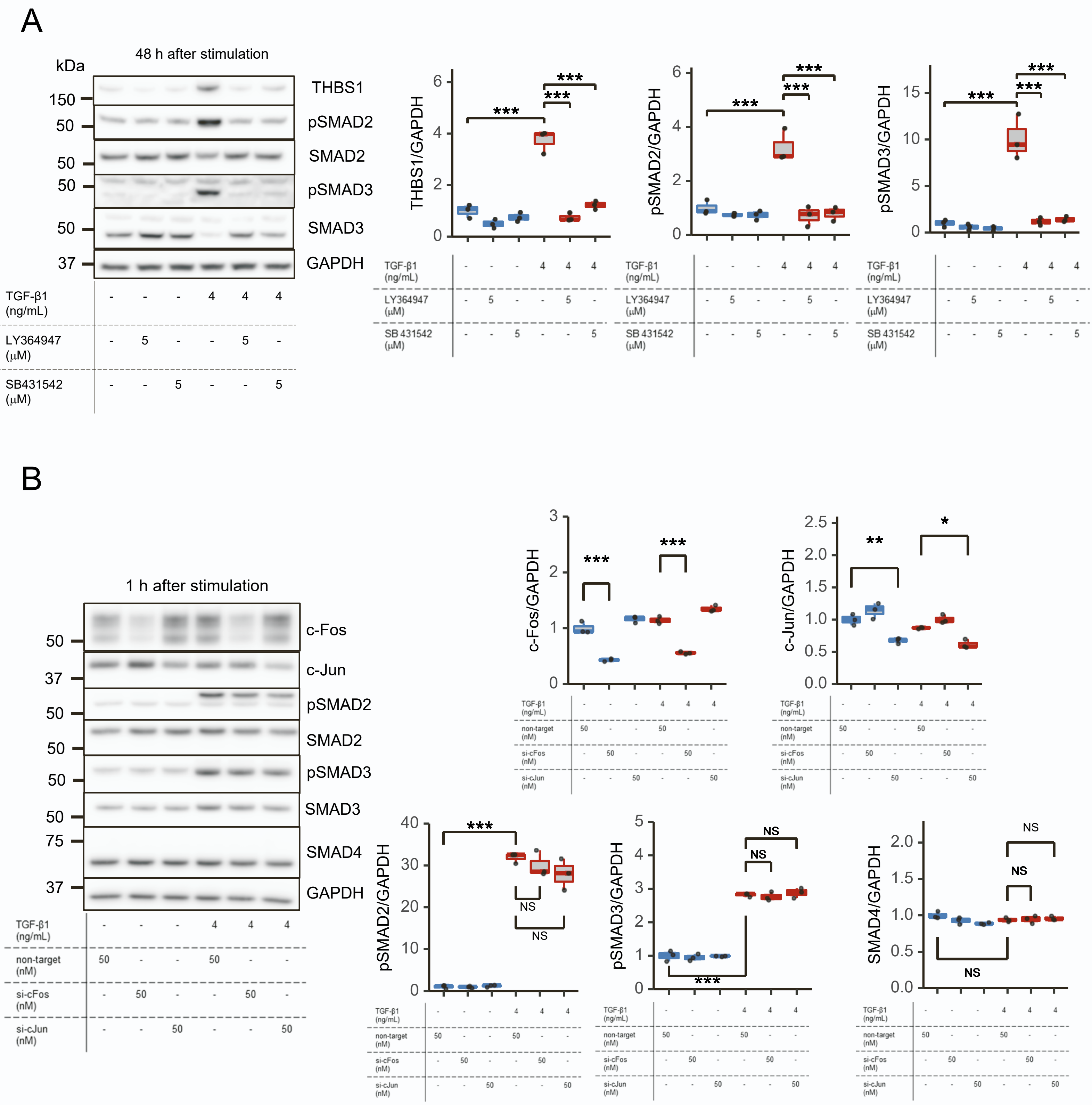
A
48 h after stimulation
kDa
150
50
50
50
50
37
THBS1
pSMAD2
SMAD2
pSMAD3
SMAD3
GAPDH
TGF-β1 (ng/mL)
LY364947 (μM)
SB431542 (μM)
THBS1/GAPDH
pSMAD2/GAPDH
pSMAD3/GAPDH
***
B
1 h after stimulation
c-Fos
c-Jun
pSMAD2
SMAD2
pSMAD3
SMAD3
SMAD4
GAPDH
TGF-β1 (ng/mL)
non-target (nM)
si-cFos (nM)
si-cJun (nM)
c-Fos/GAPDH
c-Jun/GAPDH
pSMAD2/GAPDH
pSMAD3/GAPDH
SMAD4/GAPDH
***
**
*
NS

Figure S5: Effect of TGF-βR inhibitors and c-Fos/c-Jun knockdown (KD) on SMAD activation, related to Figure 3.

A) WB analysis of TGF-β1-stimulated HFF-1 cells treated with TGF-βR inhibitors. Cells were treated with LY364947 and SB431542 with/without TGF-β1 for 48 h and the lysates were analyzed. (Left panel) Representative image. (Right panel) Quantification of THBS1, pSMAD2, and pSMAD3 expression following TGF-βR inhibitor treatment; N=3, ***p<0.001 (Tukey's multiple comparisons).

B) WB analysis of c-Fos/c-Jun KD in HFF-1 cells. c-Fos/c-Jun KD cell lysates were collected at 1 h after control (blue) or TGF-β1 (red) treatment. (Left panel) Representative image. (Right panel) Quantification of c-Fos, c-Jun, pSMAD2, pSMAD3, and SMAD4; N=3, *p<0.05, **p<0.01, ***p<0.001, NS: not significant (Tukey's multiple comparisons).

A

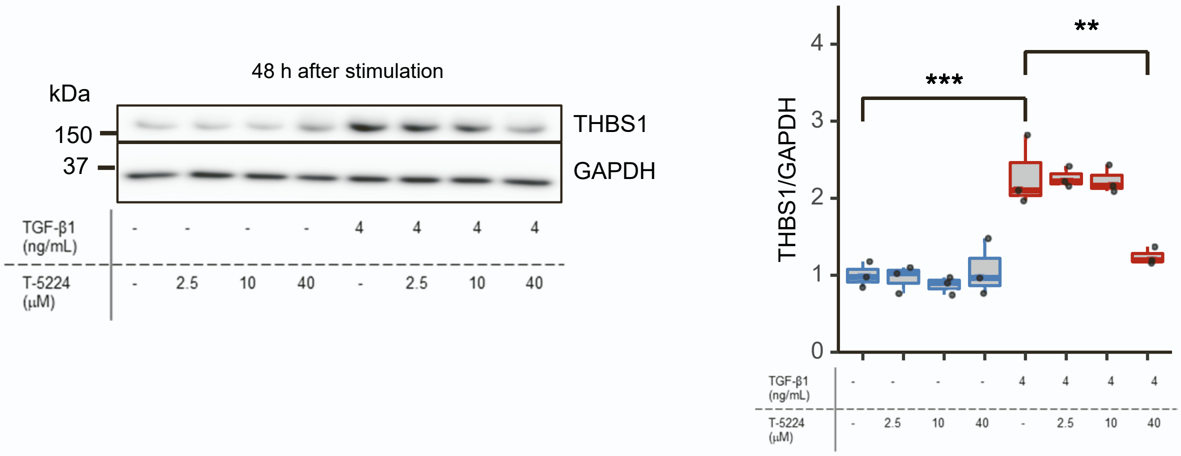
48 h after stimulation
kDa
150
37
THBS1
GAPDH
TGF-β1 (ng/mL)
T-5224 (μM)
- - - - 4 4 4 4
- 2.5 10 40 - 2.5 10 40
THBS1/GAPDH
0 1 2 3 4
***
**

B

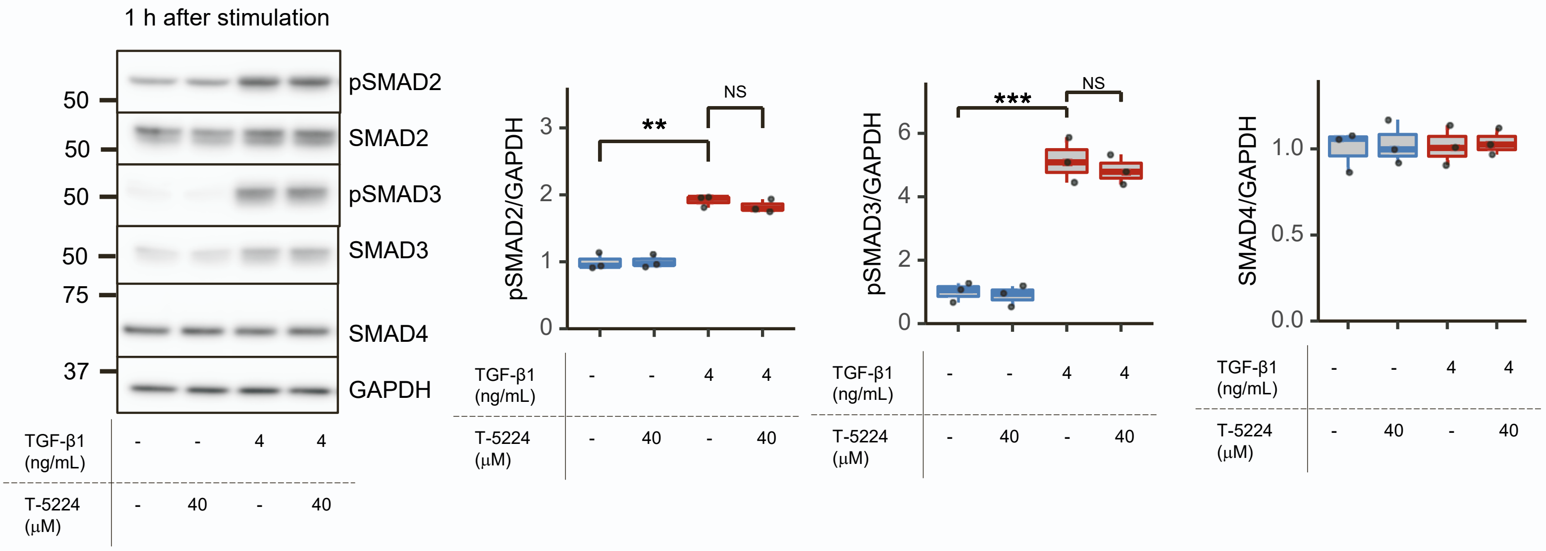
1 h after stimulation
50
50
50
50
75
37
pSMAD2
SMAD2
pSMAD3
SMAD3
SMAD4
GAPDH
TGF-β1 (ng/mL)
T-5224 (μM)
- - 4 4
- 40 - 40
pSMAD2/GAPDH
0 1 2 3
**
NS
pSMAD3/GAPDH
0 2 4 6
***
NS
SMAD4/GAPDH
0.0 0.5 1.0

Figure S6: Effect of c-Fos/c-Jun inhibitor T-5224 on THBS1 and SMAD activation, related to Figure 3.

A) WB analysis of TGF-β1-stimulated HFF-1 cells treated with T-5224. Cells were treated with/without T-5224, and TGF-β1 for 48 h and the lysates were analyzed. (Left panel) Representative image. (Right panel) Quantification of THBS1; N=3, $^{**}p<0.01$, $^{***}p<0.001$ (Tukey's multiple comparisons).

B) WB analysis of TGF-β1-stimulated HFF-1 cells treated with T-5224. Cells were treated with/without T-5224, and TGF-β1 for 1 h and lysates were analyzed. (Left panel) Representative image. (Right panel) Quantification of pSMAD2, pSMAD3, and SMAD4; N=3, $^{**}p<0.01$, $^{***}p<0.001$, NS: not significant (Tukey's multiple comparisons).

A

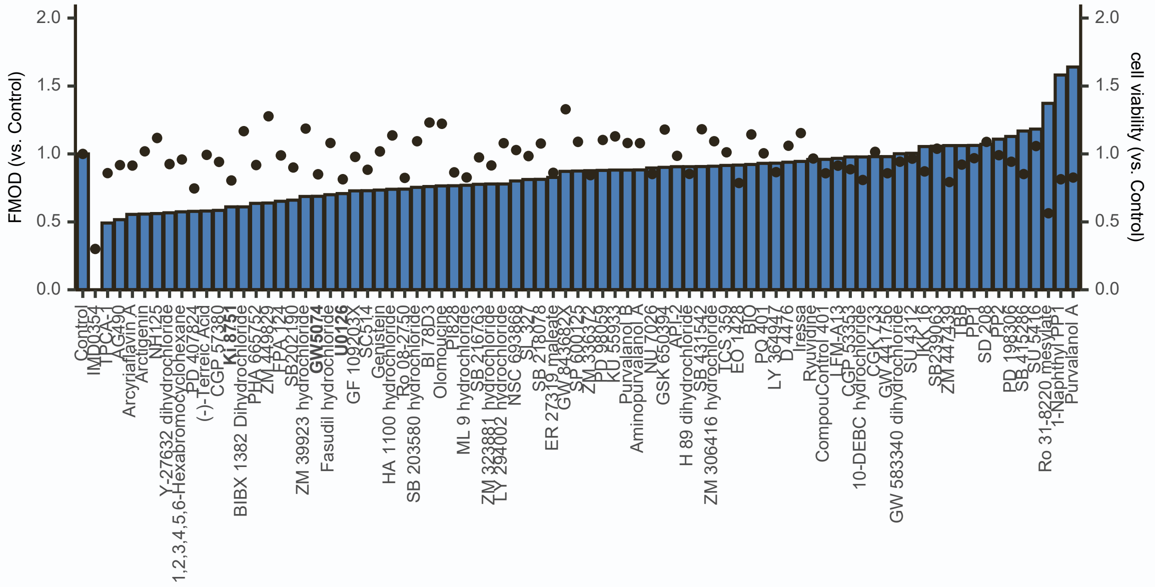

FMOD (vs. Control)
cell viability (vs. Control)
0.0
0.5
1.0
1.5
2.0
Control
IMD0354
TPCA-1
AG490
Arcyriaflavin A
Arctigenin
NH125
Y-27632 dihydrochloride
1,2,3,4,5,6-Hexabromocyclohexane
PD 407824
(-)-Terreic Acid
CGP 57380
Ki 8751
BIBX 1382 Dihydrochloride
PHA 665752
ZM 449829
FPA 124
SB202190
ZM 39923 hydrochloride
GW5074
Fasudil hydrochloride
U0126
GF 109203X
SC514
Genistein
HA 1100 hydrochloride
Ro 08-2750
SB 203580 hydrochloride
BI 78D3
Olomoucine
PI828
ML 9 hydrochloride
SB 216763
ZM 323881 hydrochloride
LY 294002 hydrochloride
NSC 693868
SL 327
SB 218078
ER 27319 maleate
GW 843682X
SP 600125
ZM 336372
PD 98059
KU 55933
Purvalanol B
Aminopurvalanol A
NU 7026
GSK 650394
API-2
H 89 dihydrochloride
SB 431542
ZM 306416 hydrochloride
TCS 359
EO 1428
BIO
PQ 401
LY 364947
D 4476
Iressa
Ryuvidine
CompouControl 401
LFM-A13
CGP 53353
10-DEBC hydrochloride
CGK 733
GW 441756
GW 583340 dihydrochloride
SU 4312
IKK 16
SB239063
ZM 447439
TBB
PP1
SD 208
PP2
PD 198306
SB 415286
SU 5416
Ro 31-8220 mesylate
1-Naphthyl PP1
Purvalanol A

B

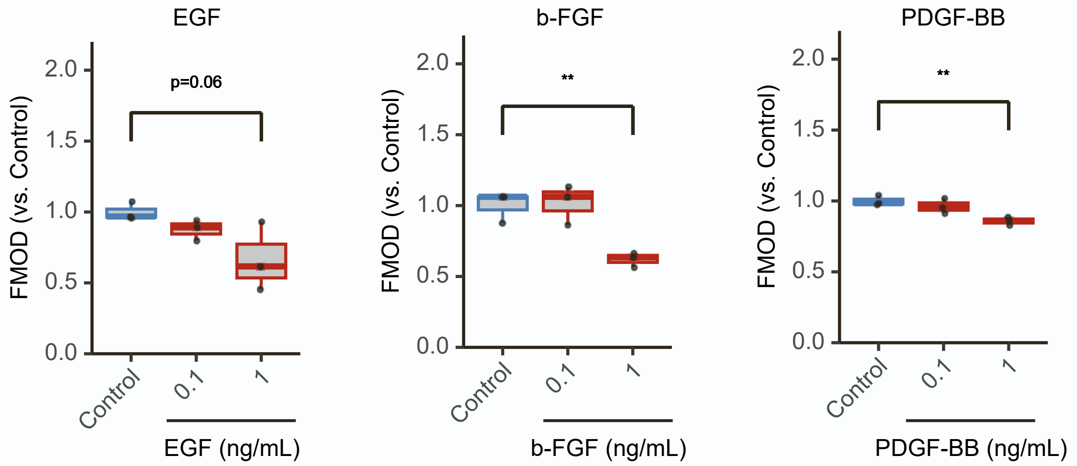

EGF
p=0.06
FMOD (vs. Control)
0.0
0.5
1.0
1.5
2.0
Control
0.1
1
EGF (ng/mL)
b-FGF
**
FMOD (vs. Control)
0.0
0.5
1.0
1.5
2.0
Control
0.1
1
b-FGF (ng/mL)
PDGF-BB
**
FMOD (vs. Control)
0.0
0.5
1.0
1.5
2.0
Control
0.1
1
PDGF-BB (ng/mL)

Figure S7: Quantification of FMOD on kinase inhibitors screening and EGF, b-FGF, or PDGF-BB treatment, related to Figure 3.

A) FMOD ELISA in kinase inhibitors-screening of HFF-1 cells. Cells were treated with various kinase inhibitors for 48 h and their supernatants were analyzed. (Left axis) Quantification of FMOD (vs. control). (Right axis) Cell viability (vs. control) measured using the tetrazolium salt WST-8; N=1.

B) FMOD epidermal growth factor (EGF), basic-fibroblast growth factor (b-FGF), or platelet derived growth factor-BB (PDGF-BB) treatment of HFF-1 cells. Cells were treated with each ligand for 48 h and their supernatants were analyzed; N=3, **$p<0.01$ (Dunnett's test).

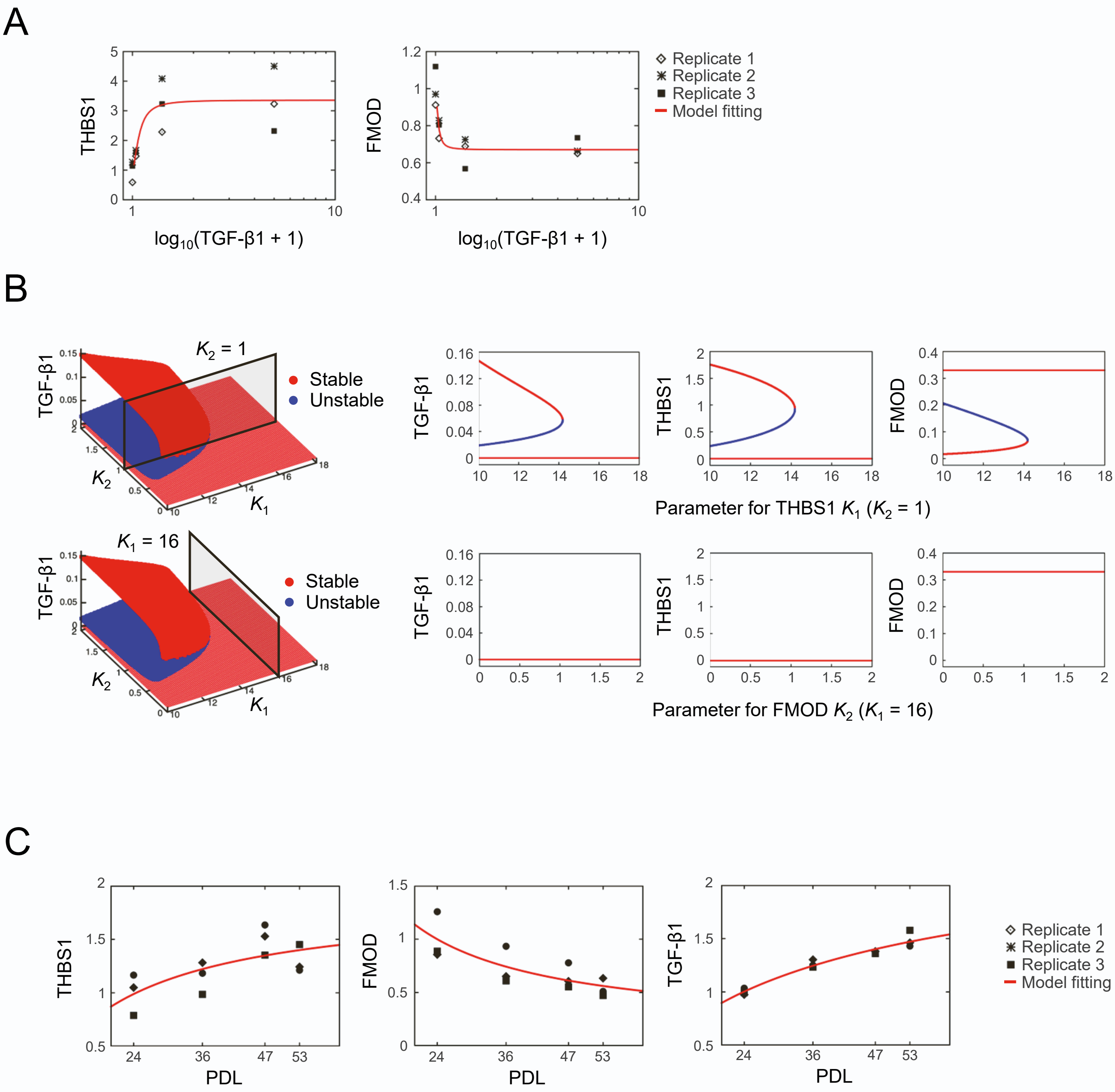
A
THBS1
log10(TGF-β1 + 1)
FMOD
log10(TGF-β1 + 1)
Replicate 1
Replicate 2
Replicate 3
Model fitting
B
TGF-β1
K2 = 1
Stable
Unstable
K2
K1
Parameter for THBS1 K1 (K2 = 1)
K1 = 16
Parameter for FMOD K2 (K1 = 16)
THBS1
FMOD
C
THBS1
PDL
FMOD
PDL
TGF-β1
PDL
Replicate 1
Replicate 2
Replicate 3
Model fitting

Figure S8: Data fitting of bifurcation analysis, related to Figure 4.

A) Model parameters for the core network consisting of TGF-β1, THBS1, and FMOD were trained on the experimental expressions of THBS1 and FMOD upon treatment with different concentrations (0.04, 0.4, or 4 ng/mL) of TGF-β1 (Figure 3A). The x- and y-axes represent steady state expressions of TGF-β1 and THBS1 (left panel) or FMOD (right panel). The points (◇: Replicate 1, ⁎: Replicate 2, ■: Replicate 3) indicate experimental data, and red solid lines indicate model fitting results.

B) Bifurcation diagrams of steady state expressions of TGF-β1, THBS1, and FMOD, with respect to K_1 (upper panel) and K_2 (bottom panel), which correspond to the cut surfaces shown in left panels, respectively. Red and blue colors represent stable and unstable points on the surface, respectively. Solutions with high TGF-β1 expression levels indicate a high-THBS1 (low-FMOD) state, while those with low TGF-β1 expression levels indicate a low-THBS1 (high-FMOD) state.

C) Model parameters for the core network consisting of endogenous effects of TGF-β1, THBS1, and FMOD were trained on the experimental expression of THBS1, FMOD, and TGF-β1 upon replication-stress with different PDL (PDL 24, PDL 36, PDL 47, PDL 53) (Figure 2A, 2B). The x- and y-axes represent steady state expressions of PDL and THBS1 (upper panel), FMOD (right panel), or TGF-β1 (bottom panel). The points (◇: Replicate 1, ⁎: Replicate 2, ■: Replicate 3) indicate experimental data, and red solid lines indicate model fitting results.

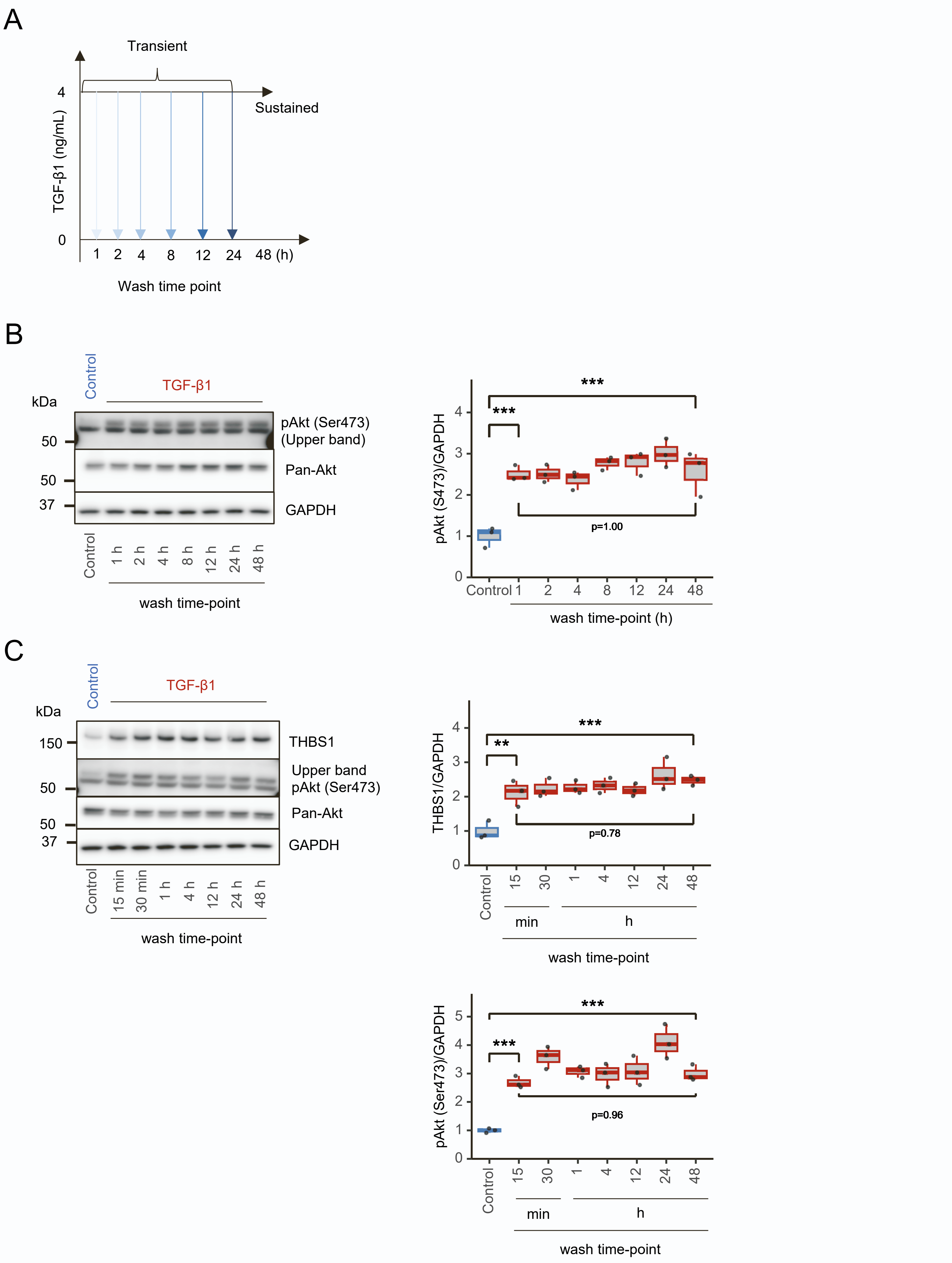
A
Transient
Sustained
TGF-β1 (ng/mL)
4
0
1 2 4 8 12 24 48 (h)
Wash time point
B
Control
TGF-β1
kDa
50
50
37
pAkt (Ser473)
(Upper band)
Pan-Akt
GAPDH
Control 1 h 2 h 4 h 8 h 12 h 24 h 48 h
wash time-point
pAkt (S473)/GAPDH
***
***
p=1.00
Control 1 2 4 8 12 24 48
wash time-point (h)
C
Control
TGF-β1
kDa
150
50
50
37
THBS1
Upper band
pAkt (Ser473)
Pan-Akt
GAPDH
Control 15 min 30 min 1 h 4 h 12 h 24 h 48 h
wash time-point
THBS1/GAPDH
***
**
p=0.78
Control 15 30 1 4 12 24 48
min
h
wash time-point
pAkt (Ser473)/GAPDH
***
***
p=0.96
Control 15 30 1 4 12 24 48
min
h
wash time-point

Figure S9: Validation with washout experiment of bifurcation analysis, related to Figure 4.

A) Schematic representation of TGF-β1 washout experiment. The x- and y-axes represent the washout time-points and concentration dynamics of TGF-β1, respectively.

B) WB analysis of TGF-β1 washout experiment using HFF-1 cells. Cells were treated with TGF-β1, washed with PBS at each time-point (1 h, 2 h, 4 h, 8 h, 12 h, 24 h, and 48 h), and lysates were collected at 48 h. (Left panel) Representative image. (Right panel) Quantification of phosphorylated Akt (Ser473); N=3, ***p<0.001 (Tukey's multiple comparisons).

C) WB analysis of TGF-β1 washout experiment using HFF-1 cells. Cells were treated with TGF-β1, washed with PBS at each time-point (15 min, 30 min, 1 h, 4 h, 12 h, 24 h, and 48 h), and lysates were collected at 48 h. (Left panel) Representative image. (Upper right panel) Quantification of THBS1; N=3, **p<0.01, ***p<0.001 (Tukey's multiple comparisons). (Bottom right panel) Quantification of phosphorylated Akt (Ser473); N=3, ***p<0.001 (Tukey's multiple comparisons).

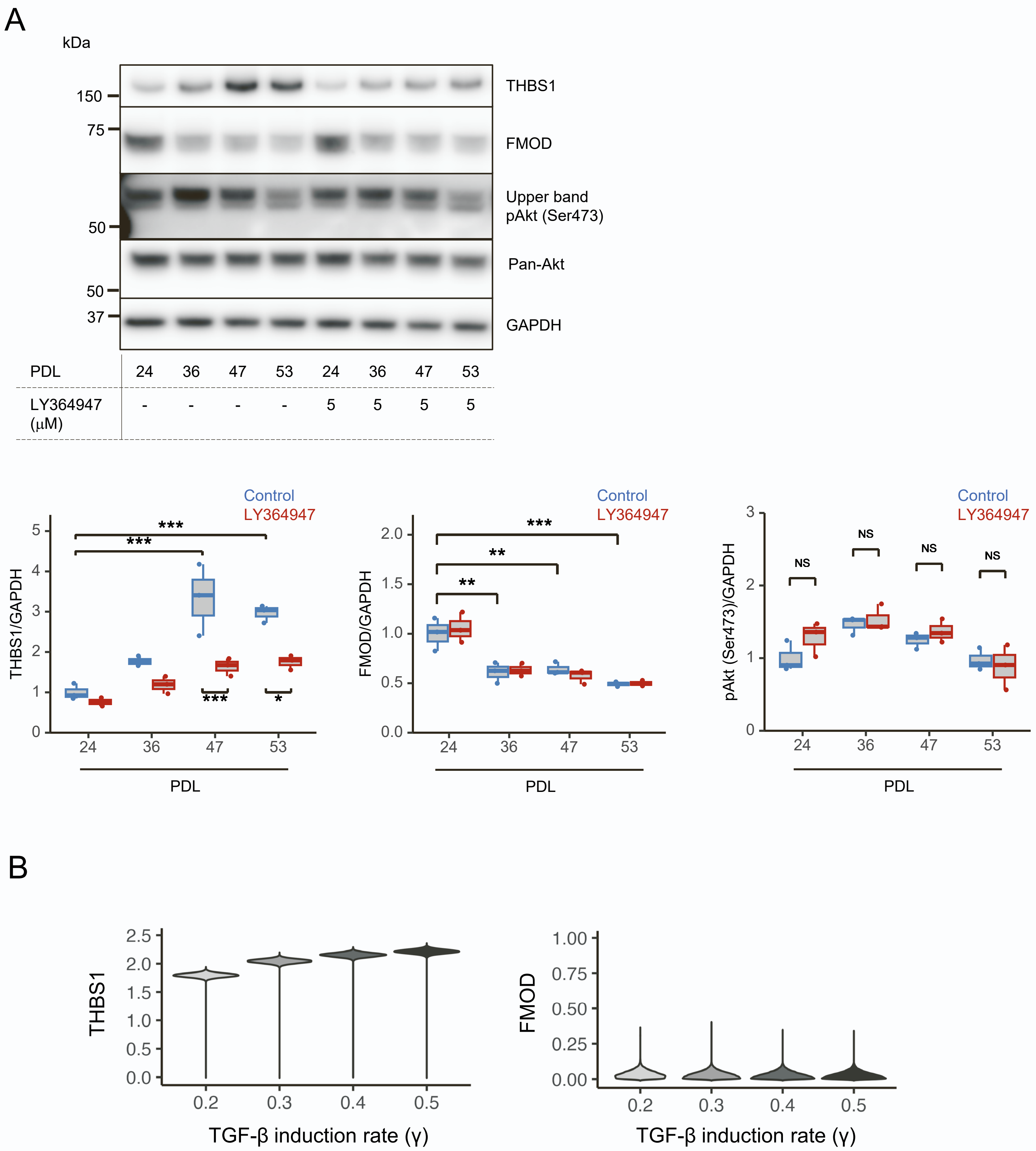

A
kDa
150
75
50
50
37
THBS1
FMOD
Upper band
pAkt (Ser473)
Pan-Akt
GAPDH
PDL
24 36 47 53 24 36 47 53
LY364947
(μM)
- - - - 5 5 5 5
Control
LY364947
THBS1/GAPDH
FMOD/GAPDH
pAkt (Ser473)/GAPDH
***
**
*
NS
PDL
B
THBS1
FMOD
TGF-β induction rate (γ)

Figure S10: Roles of endogenous TGF-β signaling on THBS1 and FMOD, related to Figure 4.

A) WB analysis of replication-stress-induced HFF-1 cells treated with the TGF-βR inhibitor. Cells of each PDL (PDL 24, PDL 36, PDL 47, PDL 53) were treated with LY364947 for 48 h and the lysates were analyzed. (Upper panel) Representative image. (Bottom left panel) Quantification of THBS1; N=3, *p<0.05, ***p<0.001 (Tukey's multiple comparisons). (Bottom middle panel) Quantification of FMOD; N=3, **p<0.01, ***p<0.001 (Tukey's multiple comparisons). (Bottom right panel) Quantification of phosphorylated Akt (Ser473); N=3, NS: not significant (Tukey's multiple comparisons).

B) Probability distributions for the expression levels of THBS1 (left panel) and FMOD (right panel) with respect to endogenous TGF-β1 production rate γ, as computed using a stochastic model with endogenous parameters and 10,000 independent simulations.

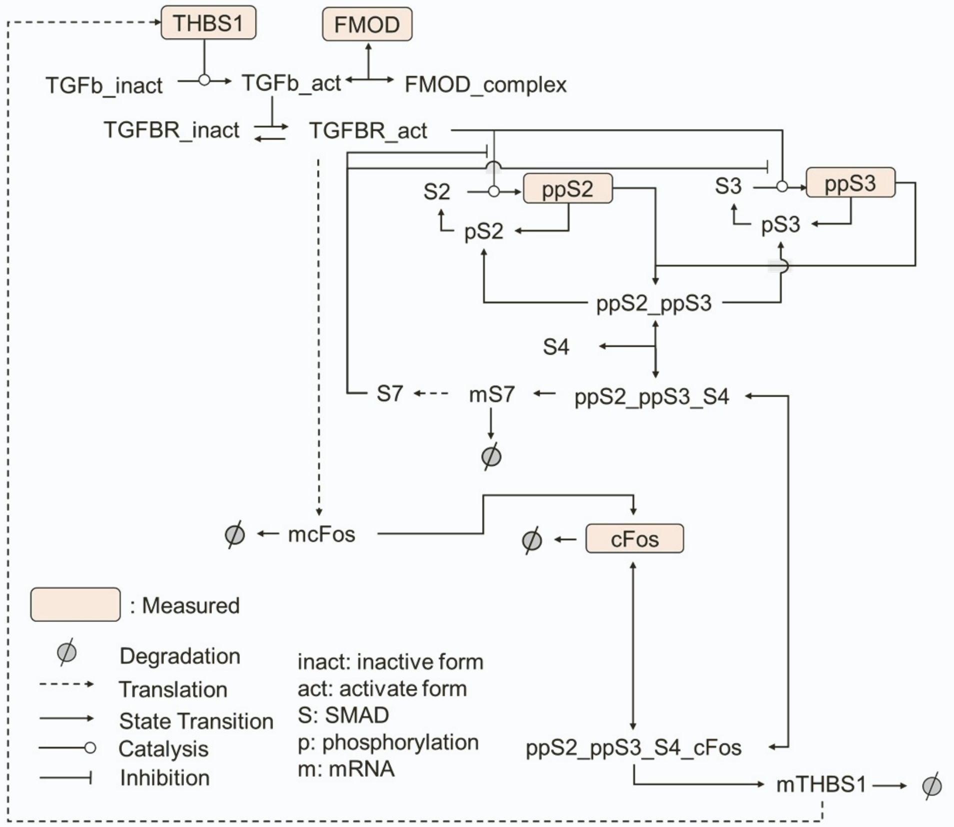
THBS1
FMOD
TGFb_inact
TGFb_act
FMOD_complex
TGFBR_inact
TGFBR_act
S2
ppS2
pS2
S3
ppS3
pS3
ppS2_ppS3
S4
S7
mS7
ppS2_ppS3_S4
mcFos
cFos
: Measured
Degradation
Translation
State Transition
Catalysis
Inhibition
inact: inactive form
act: activate form
S: SMAD
p: phosphorylation
m: mRNA
ppS2_ppS3_S4_cFos
mTHBS1

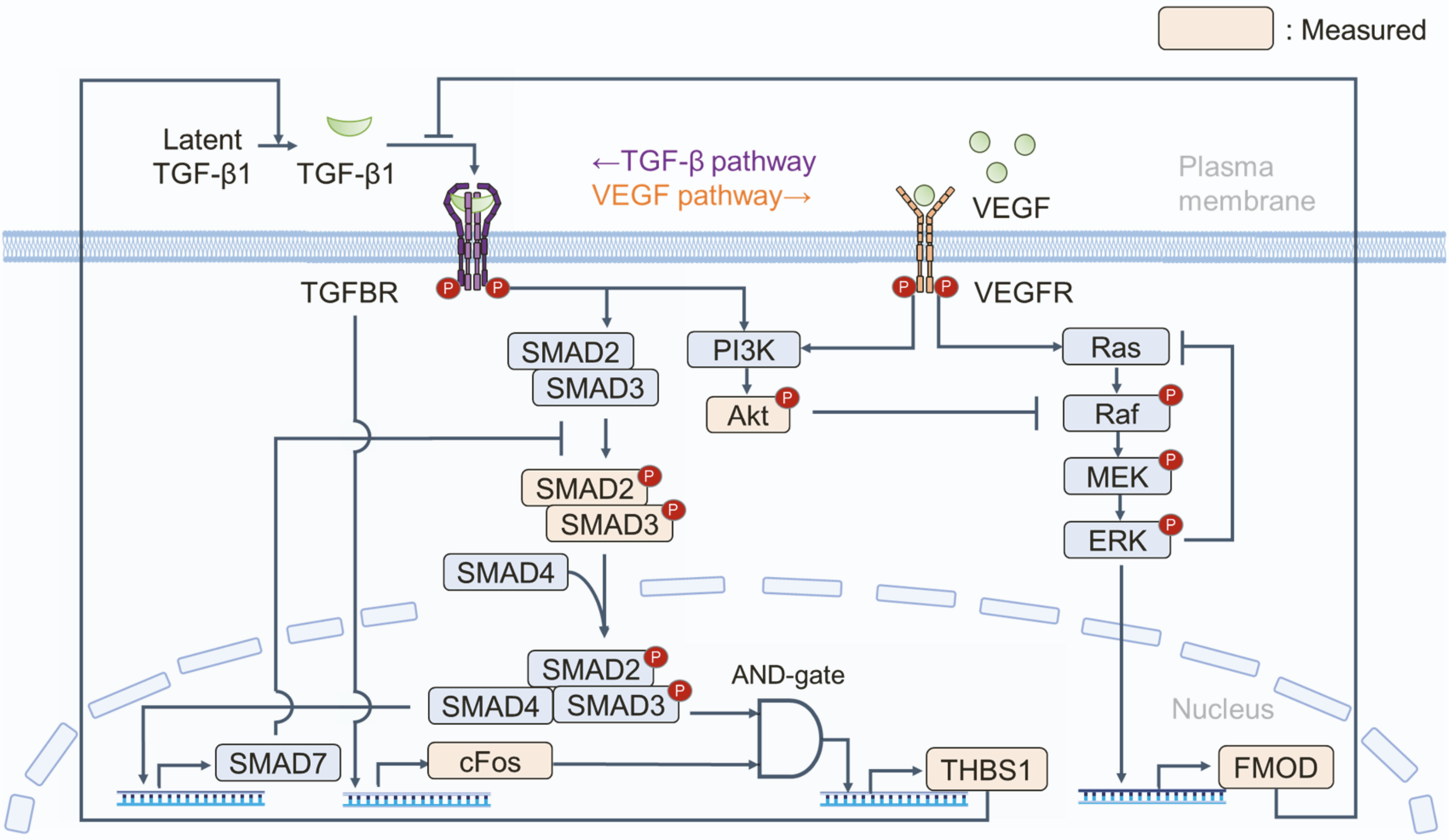
: Measured
Latent TGF-β1
TGF-β1
←TGF-β pathway
VEGF pathway→
VEGF
Plasma membrane
TGFBR
VEGFR
SMAD2
SMAD3
PI3K
Ras
Akt
Raf
MEK
ERK
SMAD4
AND-gate
Nucleus
SMAD7
cFos
THBS1
FMOD

Figure S11: Mathematical model diagram of the TGF-β–VEGF integrated signaling pathway, related to Figure 5 and STAR Methods.

TGF-β and VEGF signaling pathways in an ordinary differential equation model. (Upper panel) Diagram of molecular interactions in the TGF-β signaling model. A model of TGF-βR activation, SMAD phosphorylation, and SMAD complex formation was developed for the TGF-β pathway. (Lower panel) In addition to the TGF-β signaling model, the process of VEGFR activation to ERK phosphorylation was described using the Imoto model[53] (see details in STAR Methods section).

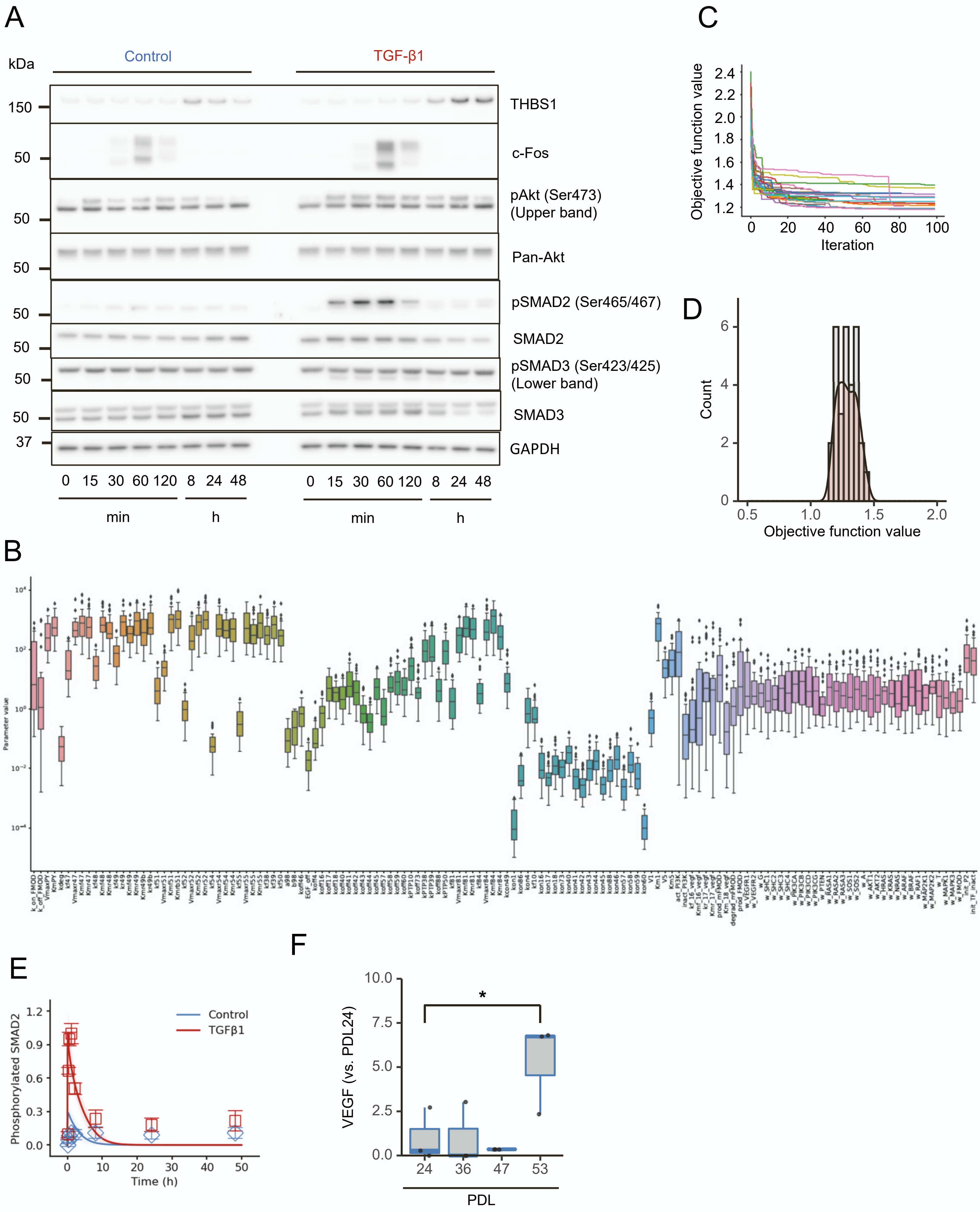
A
kDa
Control
TGF-β1
150
50
50
50
50
50
50
50
37
THBS1
c-Fos
pAkt (Ser473)
(Upper band)
Pan-Akt
pSMAD2 (Ser465/467)
SMAD2
pSMAD3 (Ser423/425)
(Lower band)
SMAD3
GAPDH
0 15 30 60 120 8 24 48
0 15 30 60 120 8 24 48
min
h
min
h
C
Objective function value
2.4
2.2
2.0
1.8
1.6
1.4
1.2
0 20 40 60 80 100
Iteration
D
Count
6
4
2
0
0.5 1.0 1.5 2.0
Objective function value
B
Parameter value
E
Phosphorylated SMAD2
1.2
0.9
0.6
0.3
0.0
Control
TGFβ1
0 10 20 30 40 50
Time (h)
F
VEGF (vs. PDL24)
10.0
7.5
5.0
2.5
0.0
*
24 36 47 53
PDL

Figure S12: Mathematical modeling and validation of integrated TGF-β–VEGF signaling pathway, related to Figure 5.

A) Experimental time-course WB image with/without TGF-β1 treatment in HFF-1 cells. HFF-1 was stimulated with/without TGF-β1 and the lysates were collected at 0 min, 15 min, 30 min, 60 min, 120 min, 8 h, 24 h, and 48 h. Representative image, N=3.

B) Estimated parameter values of 30 parameter sets for integrated TGF-β–VEGF signaling model.

C) Objective function traces from 30 optimization runs for the integrated TGF-β–VEGF signaling model.

D) Distribution of objective function value of 30 parameter sets. Bar graph: histogram of the count. Shade: density of the count.

E) Validation of mechanistic model using phosphorylated SMAD2. The model reproduced phosphorylated SMAD2 dynamics. The points (control: blue squares; 4 ng/mL TGF-β1: red squares) indicate experimental data, solid lines indicate the average simulation of 30 parameter sets, and shaded areas indicate SD; N=3, mean ± SD.

F) VEGF ELISA in replication-stress-induced HFF-1 cells. Cells of each PDL (PDL 24, PDL 36, PDL 47, PDL 53) were cultured for 48 h, and supernatants were analyzed by VEGF ELISA; N=3, *p<0.05 (Dunnett's test).

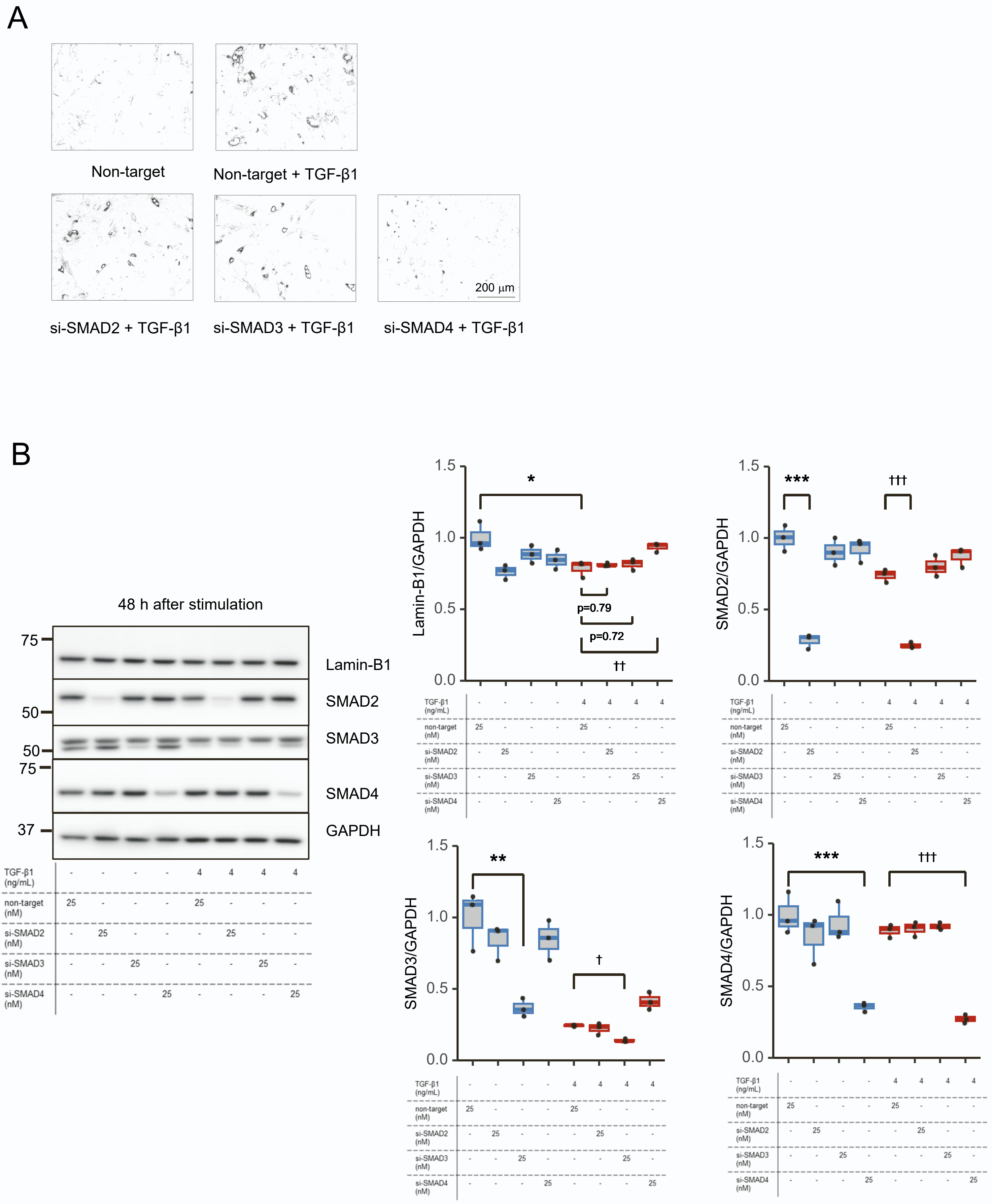
A
Non-target
Non-target + TGF-β1
si-SMAD2 + TGF-β1
si-SMAD3 + TGF-β1
si-SMAD4 + TGF-β1
200 μm
B
48 h after stimulation
75
50
50
75
37
Lamin-B1
SMAD2
SMAD3
SMAD4
GAPDH
TGF-β1 (ng/mL)
non-target (nM)
si-SMAD2 (nM)
si-SMAD3 (nM)
si-SMAD4 (nM)
Lamin-B1/GAPDH
p=0.79
p=0.72
SMAD2/GAPDH
SMAD3/GAPDH
SMAD4/GAPDH

Figure S13: Effect of siRNA KD of SMADs on SA-β-gal staining, Lamin-B1, and KD efficacy (48 h), related to Figure 6.

A) SA-β-gal staining of siRNA KD of SMADs using HFF-1 cells; scale bars: 200 μm. Each image was processed using Image J (see details in STAR Methods section). Raw images are shown in Figure 6C.

B) WB analysis of Lamin-B1 and SMADs following siRNA KD of SMADs in HFF-1 cells. Cells were pretreated with each siRNA (25 nM) and collected 48 h after control (blue) or 4 ng/mL TGF-β1 (red) treatment. (Left panel) Representative image. (Upper middle panel) Quantification of Lamin-B1; N=3, *p<0.05 (Student's *t*-test), ††p<0.01 (Dunnett's test). (Upper right panel) Quantification of SMAD2; N=3, ***p<0.001 (Dunnett's test), †††p<0.001 (Dunnett's test). (Bottom middle panel) Quantification of SMAD3; N=3, **p<0.01 (Dunnett's test), †p<0.05 (Dunnett's test). (Bottom right panel) Quantification of SMAD4; N=3, ***p<0.001 (Dunnett's test), †††p<0.001 (Dunnett's test).

Table S1: Donor list used for public RNA-seq analysis, related to Figure 1 and S2.

Detailed information on donors in public *in vivo* expression data[27].

Run	Age (years)	Disease	Gender	Source	Ethnicity	Sample filtering
SRR7093887	11	Healthy	Female	Skin; Arm	Caucasian	Omitted
SRR7093888	11	Healthy	Female	Skin; Arm	Caucasian	Used
SRR7093889	12	Healthy	Male	Skin; Arm	Caucasian	Used
SRR7093897	19	Healthy	Male	Skin; Arm	Caucasian	Used
SRR7093899	20	Healthy	Female	Skin; Arm	Caucasian	Used
SRR7093811	24	Healthy	Female	Skin; Arm	Caucasian	Used
SRR7093930	24	Healthy	Male	Skin; Arm	Caucasian	Used
SRR7093812	25	Healthy	Male	Skin; Arm	Caucasian	Used
SRR7093813	25	Healthy	Female	Skin; Arm	Caucasian	Used
SRR7093928	25	Healthy	Female	Skin; Arm	Caucasian	Used
SRR7093814	26	Healthy	Male	Skin; Arm	Caucasian	Used
SRR7093815	26	Healthy	Female	Skin; Arm	Caucasian	Used
SRR7093816	28	Healthy	Male	Skin; Arm	Caucasian	Used
SRR7093817	29	Healthy	Male	Skin; Arm	Caucasian	Used
SRR7093818	29	Healthy	Male	Skin; Arm	Caucasian	Omitted
SRR7093819	30	Healthy	Male	Skin; Arm	Caucasian	Used
SRR7093820	30	Healthy	Female	Skin; Arm	Caucasian	Used
SRR7093821	30	Healthy	Male	Skin; Arm	Caucasian	Omitted
SRR7093822	30	Healthy	Male	Skin; Arm	Caucasian	Used
SRR7093908	37	Healthy	Male	Skin; Arm	Caucasian	Used
SRR7093909	39	Healthy	Male	Skin; Arm	Caucasian	Omitted
SRR7093825	41	Healthy	Male	Skin; Arm	Caucasian	Used
SRR7093832	41	Healthy	Female	Skin; Arm	Caucasian	Omitted
SRR7093830	42	Healthy	Female	Skin; Arm	Caucasian	Used
SRR7093823	43	Healthy	Female	Skin; Arm	Caucasian	Used
SRR7093833	43	Healthy	Male	Skin; Arm	Caucasian	Used
SRR7093824	44	Healthy	Male	Skin; Arm	Caucasian	Used
SRR7093834	44	Healthy	Male	Skin; Arm	Caucasian	Used
SRR7093835	45	Healthy	Male	Skin; Arm	Caucasian	Used
SRR7093828	46	Healthy	Male	Skin; Arm	Caucasian	Used
SRR7093836	46	Healthy	Male	Skin; Arm	Caucasian	Omitted
SRR7093837	46	Healthy	Male	Skin; Arm	Caucasian	Used
SRR7093826	47	Healthy	Male	Skin; Arm	Caucasian	Used
SRR7093831	47	Healthy	Male	Skin; Arm	Caucasian	Used
SRR7093838	47	Healthy	Male	Skin; Arm	Caucasian	Used
SRR7093827	50	Healthy	Female	Skin; Arm	Caucasian	Used
SRR7093829	50	Healthy	Male	Skin; Arm	Caucasian	Used
SRR7093910	51	Healthy	Male	Skin; Arm	Caucasian	Omitted
SRR7093912	55	Healthy	Male	Skin; Arm	Caucasian	Omitted
SRR7093839	61	Healthy	Male	Skin; Arm	Caucasian	Used
SRR7093840	62	Healthy	Female	Skin; Arm	Caucasian	Used
SRR7093841	62	Healthy	Female	Skin; Arm	Caucasian	Used
SRR7093842	63	Healthy	Male	Skin; Arm	Caucasian	Used
SRR7093843	64	Healthy	Male	Skin; Arm	Caucasian	Used
SRR7093844	66	Healthy	Male	Skin; Arm	Caucasian	Used
SRR7093845	67	Healthy	Male	Skin; Arm	Caucasian	Used
SRR7093846	67	Healthy	Male	Skin; Arm	Caucasian	Used
SRR7093847	68	Healthy	Male	Skin; Arm	Caucasian	Omitted
SRR7093848	68	Healthy	Male	Skin; Arm	Caucasian	Used
SRR7093939	68	Healthy	Male	Skin; Arm	Caucasian	Omitted
SRR7093849	69	Healthy	Male	Skin; Arm	Caucasian	Used
SRR7093850	69	Healthy	Female	Skin; Arm	Caucasian	Used
SRR7093941	69	Healthy	Male	Skin; Arm	Caucasian	Used
SRR7093940	70	Healthy	Male	Skin; Arm	Caucasian	Used
SRR7093915	71	Healthy	Female	Skin; Arm	Caucasian	Used

Table S2: Donor list of dermal tissue, related to Figure 2.

Detailed information on donors used to confirm protein expression in human dermal tissues.

Donor ID	Age (years)	Part	Gender	Race	Frozen time (Count)	Body mass index
#1	23	Breast	Female	Caucasian	1	23
#2	27	Abdominal	Female	Caucasian	1	24
#3	31	Breast	Female	Caucasian	1	31
#4	46	Abdominal	Female	Caucasian	1	21
#5	61	Breast	Female	Caucasian	1	29
#6	63	Abdominal	Female	Caucasian	1	28

Table S3: Donor list used for public RNA-seq analysis (healthy and Hutchinson–Gilford progeria syndrome [HGPS] samples), related to Figure S3.

Detailed information on healthy and HGPS donors in public *in vivo* expression data[27].

Run	Age (years)	Disease	Gender	Source	Ethnicity
SRR7093809	1	Healthy	Male	Skin; Foreskin	Asian
SRR7093874	1	Healthy	Male	Skin; Unspecified	Caucasian
SRR7093875	2	Healthy	Female	Skin; Unspecified	Caucasian
SRR7093876	3	Healthy	Male	Skin; Inguinal area	Latino/Hispanic
SRR7093877	3	Healthy	Male	Skin; Unspecified	NA
SRR7093878	5	Healthy	Male	Skin; Umbilical cord area	Black
SRR7093879	6	Healthy	Male	Skin; Inguinal area	Black
SRR7093880	7	Healthy	Male	Skin; Inguinal area	Black
SRR7093881	7	Healthy	Male	Skin; Unspecified	Caucasian
SRR7093882	8	Healthy	Male	Skin; Unspecified	Caucasian
SRR7093883	8	Healthy	Male	Skin; Inguinal area	Caucasian
SRR7093884	9	Healthy	Female	Skin; Unspecified	Black
SRR7093942	8	HGPS	Female	Skin; Leg	Caucasian
SRR7093943	8	HGPS	Male	NA	NA
SRR7093944	2 + 3 months	HGPS	Female	NA	NA
SRR7093945	3 + 9 months	HGPS	Female	NA	NA
SRR7093946	4 + 8 months	HGPS	Female	NA	NA
SRR7093947	8 + 6 months	HGPS	Male	NA	NA
SRR7093948	6 + 11 months	HGPS	Female	NA	NA
SRR7093949	5	HGPS	Female	NA	NA
SRR7093950	8 + 10 months	HGPS	Male	NA	NA
SRR7093951	3	HGPS	Female	NA	NA

Table S4: List of genes, related to STAR Methods.

The list of genes used to estimate the initial protein value in the mathematical model and the corresponding transcripts per million (TPM) values.

Gene symbol	TPM (mean)	TPM (SD)
TGFBR1	64.76	2.24
TGFBR2	95.81	4.21
SMAD7	20.62	3.33
FOS	1.17	0.62
THBS1	4522.39	40.42
FMOD	20.88	0.66
FLT1	101.53	1.82
KDR	0.02	0.02
GRB2	128.45	0.34
SHC1	401.13	8.79
SHC2	1.16	0.49
SHC3	4.28	0.38
SHC4	1.26	0.30
PIK3CA	16.36	0.80
PIK3CB	15.10	0.89
PIK3CD	32.27	1.57
PIK3CG	0.00	0.00
PTEN	51.20	1.38
RASA1	85.47	4.19
RASA2	26.93	0.49
RASA3	92.99	3.57
GAB1	7.09	0.54
SOS1	25.34	1.14
SOS2	20.01	0.81
AKT1	84.62	2.25
AKT2	41.27	0.82
HRAS	101.55	4.41
KRAS	42.43	1.21
NRAS	113.71	3.80
ARAF	85.60	1.46
BRAF	4.73	0.20
RAF1	79.97	1.25
MAP2K1	73.15	1.57
MAP2K2	114.17	3.90
PTPN1	84.13	3.12
MAPK1	78.20	1.11
MAPK3	75.96	5.75
